# Nanoarchitectured *N*-Heterocyclic Carbene-Pt Nanoparticles on Carbon Nanotubes: Toward Advanced Electrocatalysis in the Hydrogen Evolution Reaction

**DOI:** 10.1021/acsami.5c02182

**Published:** 2025-03-13

**Authors:** Amalia Rapakousiou, Michail P. Minadakis, Savvas G. Chalkidis, María Luisa Ruiz-González, Cristina Navio, Georgios C. Vougioukalakis, Nikos Tagmatarchis

**Affiliations:** † Theoretical and Physical Chemistry Institute, National Hellenic Research Foundation, 48 Vassileos Constantinou Avenue, Athens 11635, Greece; ‡ Laboratory of Organic Chemistry, Department of Chemistry, National and Kapodistrian University of Athens, 15771, Athens, Greece; § Departamento de Química Inorgánica, Universidad Complutense de Madrid, 28040 Madrid, Spain; ∥ IMDEA Nanoscience, C/Faraday 9, Ciudad Universitaria de Cantoblanco, 28049 Madrid, Spain

**Keywords:** hydrogen evolution reaction (HER), electrocatalysis, platinum nanoparticles (PtNPs), N-heterocyclic carbenes (NHCs), carbon nanotubes, redox behavior, specific activity, mass activity

## Abstract

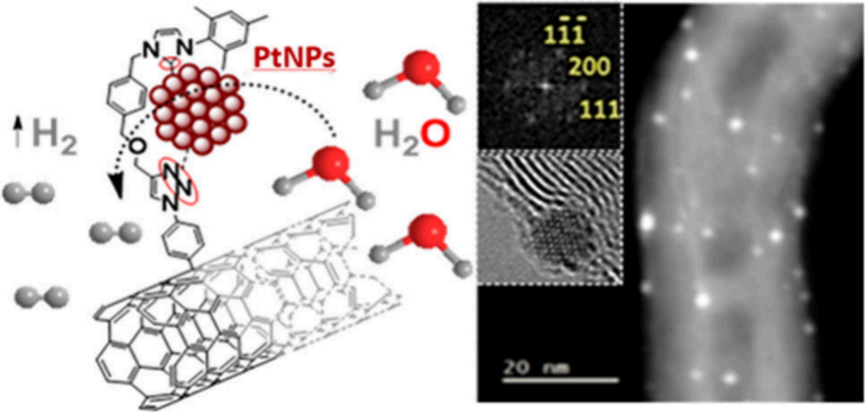

In response to the need for sustainable energy, this study focuses on advancing the electrocatalytic Hydrogen Evolution Reaction (HER). Considering platinum-based catalysts’ efficacy, but acknowledging their cost and scarcity implications, our work pursues Pt content minimization, simultaneously upholding catalytic efficiency. Our approach introduces a precisely engineered nanoarchitecture, leveraging multiwalled carbon nanotubes (MWCNTs) bearing anchored *N*-heterocyclic carbenes (NHCs). These carbenes form robust covalent bonds with ultrastable, highly crystalline, platinum nanoparticles (PtNPs), establishing MWCNTs-NHC-PtNPs as a highly efficient electrocatalyst. The synergistic effect of NHCs and triazole moieties facilitates controlled nanoparticle growth and stabilization, yielding 2.0 ± 0.3 nm, uniformly distributed {1 1 1}-faceted PtNPs. The as-obtained MWCNTs-NHC-PtNPs nanomaterial exhibits exceptional HER efficiency in 0.5 M H_2_SO_4_ with an overpotential of 77 mV at −10 mA cm^–2^ and a 50 mV dec^–1^ Tafel slope, despite containing a merely 0.4% Pt/C atomic ratio content, as determined by XPS. Notably, at 200 mV overpotential, the mass activity reaches 8.6 A/mg_Pt_ and the specific activity is 53 mA/cm^2^_Pt_, highlighting the efficiency of each Pt site within this nanostructure. Cyclic voltammetry reveals a distinctive, reversible PtO/Pt redox process, demonstrating surface-controlled and diffusion-assisted kinetics with charge storage properties that stabilize the electrocatalyst’s electron-surface and facilitate proton reduction. Equally important, the nanoarchitecture prevents aggregation and mitigates Pt irreversible oxidation, showcasing enhanced stability after extensive cycling and exposure to air. Comparative analyses with a control electrocatalyst lacking NHC-PtNPs ligation emphasize the unique role of NHC-Pt (0) bonding in enhancing electrocatalytic efficiency. Comprehensive surface and electronic property analyses validate the potential of the MWCNTs-NHC-PtNPs platform.

## Introduction

Addressing the challenge of sustainable energy production requires advanced electrocatalysts for various energy conversion processes.^1,2^ In this context, the hydrogen evolution reaction (HER) stands out as a critical cathodic process for generating “green hydrogen,” a highly versatile fuel source.^3,4^ While substantial research has focused on non-noble metal electrocatalysts for HER, platinum-based nanomaterials remain the most effective and widely used catalysts.^5,6^ However, their cost, Pt limited availability, and challenges related to performance, durability, and aggregation, require innovative solutions to enhance electrocatalytic efficiency.^7,8^ To this end, introducing nanocarriers like multiwalled carbon nanotubes (MWCNTs), effectively enhances Pt-based electrocatalytic systems due to MWCNTs’ high surface area, electrical conductivity and mechanical strength.^9^ MWCNTs interfacing nanosized Pt catalysts for applications in fuel cell devices have mostly targeted the methanol oxidation reaction (MOR) or the oxygen reduction reaction (ORR) through electrocatalysis.^10,11^

Controlling the size and shape of Pt nanoparticles (PtNPs) is vital for optimal catalytic performance in various applications.^12,13^ However, the use of shaping agents poses challenges, while the high cost and scarcity of platinum drives the need for efficient and durable electrocatalysts.^14,15^ Small PtNPs on carbon support are common, but face issues like corrosion and degradation.^16^ Furthermore, electrochemical processes vary depending on the crystal planes involved, thus influencing performance.^17,18^ As PtNPs decrease in size, the reduced proportion of {1 1 1} and {1 0 0} facets results in an abundance of low coordination sites on the surface, imposing challenges in designing efficient Pt nanocatalysts, which are crucial for clean energy technologies.^19^ Pt {1 1 1} surfaces demonstrate superior resistance to surface changes, including surface rearrangement and dissolution, confirmed by ex-situ electrochemical tests.^20,21^ Moreover, the interaction of PtNPs with Nafion, a common polymer in water electrolyzers and fuel cells, varies depending on the platinum facets, with {1 1 1} exhibiting a notable affinity, influencing reaction kinetics.^22,23^

Over the past two decades, there has been a growing interest in utilizing *N*-heterocyclic carbenes (NHCs) as versatile ligands for metal nanoparticles (MNPs).^24^ These ligands, characterized by their electron-rich nature as well as σ-donor and π-acceptor properties, have redefined many catalytic processes.^25−27^ Among others, they have the unique ability to form highly stable and robust carbon–metal bonds, offering exceptional surface tunability, stability, and reactivity to the resulting hybrid nanomaterials.^28−30^ The NHC-MNPs’ preparation commonly involves three methods: decarboxylation of NHCs carbon dioxide adducts, chemical reduction of NHC-metal compounds, or ligand exchange with preformed MNPs using various capping ligands.^31^ Currently, NHC-ligated MNPs have found various catalytic applications.^32^ Despite their great potential, the study of NHC-ligated MNPs in the field of electrocatalysis remains relatively limited, with a primary focus on the CO_2_ reduction reaction.^33−35^ To date, only a handful of studies have ventured into NHC-ligated PtNPs.^36−^^39^ However, to the best of our knowledge, the electrocatalytic potential of either supported or nonsupported NHC-ligated PtNPs remains entirely unexplored.

Here, we introduce an innovative, tailor-designed nanoarchitecture improving traditional electrocatalytic systems, by combining advanced materials with careful submolecular design. In the core of our approach, toward advanced electrocatalysts, is the strategic utilization of MWCNTs tailored with NHC ligands which in turn form, through transmetalation, covalent bonds with ultrastable, highly crystalline, {111}-faceted, ∼ 2 nm diameter, platinum nanoparticles (PtNPs) of only 0.4% Pt/C atomic loading. Our methodology involves the initial binding of tailor-made NHC-Cu (I) complexes to MWCNTs, with copper acting as a sacrificial metal. The formation of NHC-Pt (0) takes place in the presence of Pt (II) salts and sodium borohydride (NaBH_4_), driving the growth of small {1 1 1}-faceted PtNPs. Their robust covalent immobilization on the MWCNTs framework defies the limitations of noncovalent interactions, significantly enhancing the stability of our electrocatalytic system. Remarkably, this novel hybrid nanomaterial exhibits exceptional electrocatalytic activity for HER, achieving a minimal overpotential of 77 mV at −10 mA·cm^–2^ and 217 mV at −100 mA·cm^–2^, with a Tafel slope of 50 mV/dec in 0.5 M H_2_SO_4_. It also exhibits a mass activity of 8.6 A/mg_Pt_ and a specific activity of 53 mA/cm^2^_Pt_ at 200 mV, underscoring the efficiency of each Pt site within the nanostructure. Mechanistic insights indicate the direct involvement of a surface-controlled and diffusion-assisted reversible PtO/Pt redox process with pseudocapacitive characteristics, distinguishing it from traditional HER electrocatalysts. Furthermore, this electrocatalyst retains its stability and efficiency after enduring an extensive 10^4^ scan cycling, as well as its subsequent exposure to ambient air. This study highlights the great potential of our innovative electrocatalytic scaffold, using nanocarrier-immobilized NHC-bound metal nanoparticles, to address key field challenges and develop efficient, durable electrocatalysts for energy conversion.

## Results and Discussion

Our strategy for developing advanced electrocatalysts involves a ‘clickable’ NHC-precursor ligand **4** (Supporting Information (SI), Scheme S1; Figures S1–S8), specially designed to be covalently anchored to MWCNTs (**1**). Prior to their utilization, thorough cleaning of pristine MWCNTs was conducted to ensure the elimination of any residual metals in **1** (SI, pp S11, Figure S9).^40^ The modification methodology begins with the *in situ* generation of aryl diazonium salts in an inert atmosphere using 4-azidoaniline (**2**) (SI, Scheme S2; Figures S10–S12), ultimately yielding the azido-modified MWCNTs-N_3_ (**3**) (SI, Scheme S3; Figures S13–S15).^41^ This is followed by a copper(I)-catalyzed azide–alkyne cycloaddition (CuAAC) with ligand **4**, resulting in the formation of MWCNTs-imidazolium (**5**) (Scheme 1 and SI, Scheme S4; Figures S16–S19).^42,43^ Subsequently, metalation with Cu(I) using KO^t^Bu as a base generated MWCNTs-NHC-Cu (I) (**6**) (Scheme 1 and SI, Scheme S5; Figures S20–S23).

**Scheme 1:**
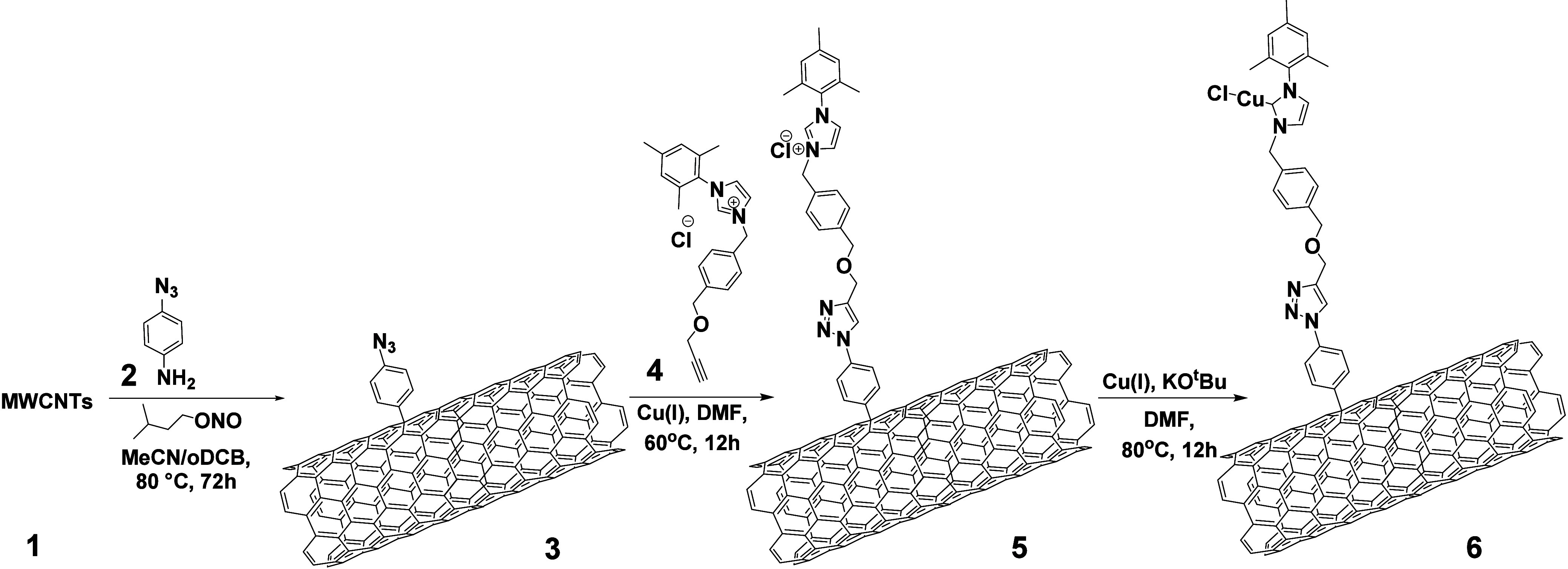
Preparation of MWCNTs-Imidazolium **5** and MWCNTs-NHC-Cu(I), **6**

**Scheme 2:**
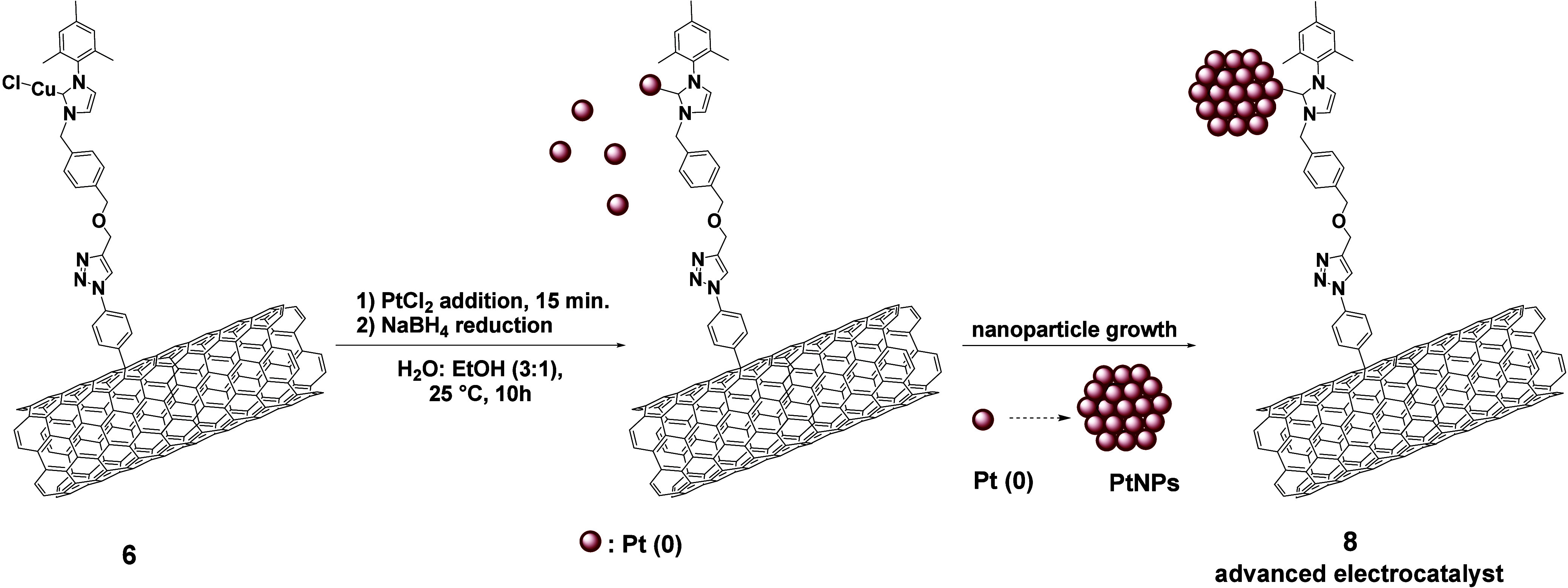
Preparation of Advanced Electrocatalyst **8**

Nanomaterial **6** incorporates 1,2,3-triazole groups along with NHC-Cu (I) complexes, with copper serving as the sacrificial metal. The triazole moieties play a dual role, facilitating the coordination of Pt (II) cations, strategically positioning them in proximity to the NHC-Cu (I) complexes, and providing mild stabilization of the resultant electroactive nanoparticles. The subsequent reaction with PtCl_2_ and NaBH_4_ in a 3/1 H_2_O: EtOH mixture leads to the reduction of Pt (II) and Cu (I) into their elemental states,^39^ followed by the transmetalation of Cu (0) with Pt (0), affording the NHC-Pt (0) species.^44−46^ This initiates the nucleation, growth and stabilization of PtNPs yielding the targeted nanoelectrocatalyst **8** (Scheme 2 and SI, Scheme S6; Figures S24–S28). The innovative design, incorporating both NHC-Cu (I) and triazole moieties, serves as the cornerstone of our approach, synergistically driving the nucleation and growth of PtNPs, enhancing the activity and stability of the electrocatalytic architecture through robust bonding formation between NHC and PtNPs.

**Scheme 3:**
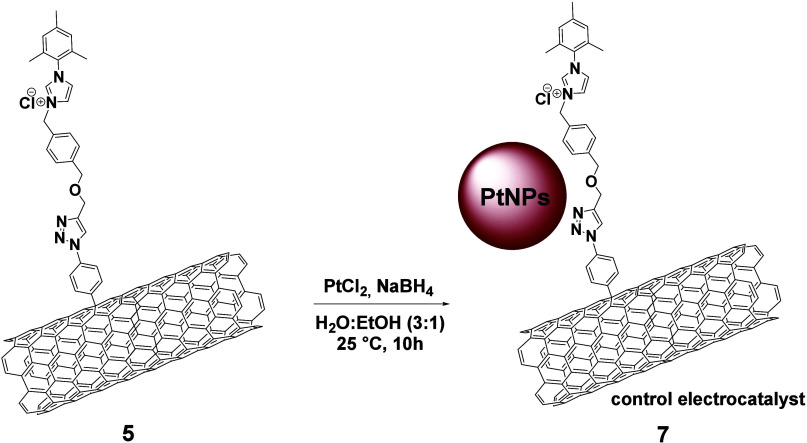
Preparation of Control Electrocatalyst **7**

To elucidate the role of NHC-PtNPs bonding in the formation of highly electroactive PtNPs covalently attached to the MWCNTs nanocarrier, a comprehensive comparative analysis was conducted, by preparing and studying a control electrocatalyst. The control electrocatalyst **7** (Scheme 3 and SI, Scheme S7; Figures S29–S32) originates from MWCNTs-imidazolium salt **5**, which is the NHC precursor to the NHC-coordinated Cu (I) species, reacting under identical experimental conditions with Pt (II) and NaBH_4_, thus leading to MWCNTs-imidazolium-PtNPs, (**7**).

Thermogravimetric analysis (TGA) revealed distinctly different thermal behavior among nanomaterials **1**, **5**, and **6** (SI, Figure S23). Purified MWCNTs (**1**) exhibit thermal stability up to 900 °C under an inert nitrogen atmosphere. In contrast, functionalized MWCNTs **5** and **6** display consistent mass losses of 18% and 19%, respectively, within the temperature range of 100–600 °C.^47^ These losses are attributed to the thermal decomposition of organic components and the introduction of structural defects within the carbon framework. The degree of functionalization on MWCNTs was estimated by normalizing the measured mass loss to the molecular weight of the organic ligand in **5**, assuming complete decomposition of the organic moieties up to 600 °C. This analysis corresponds to one ligand per 201 carbon atoms in **5**, which equally represents nanomaterials **6**, **7** and **8**, considering that no further modifications occur in the organic ligand.

**Figure 1:**
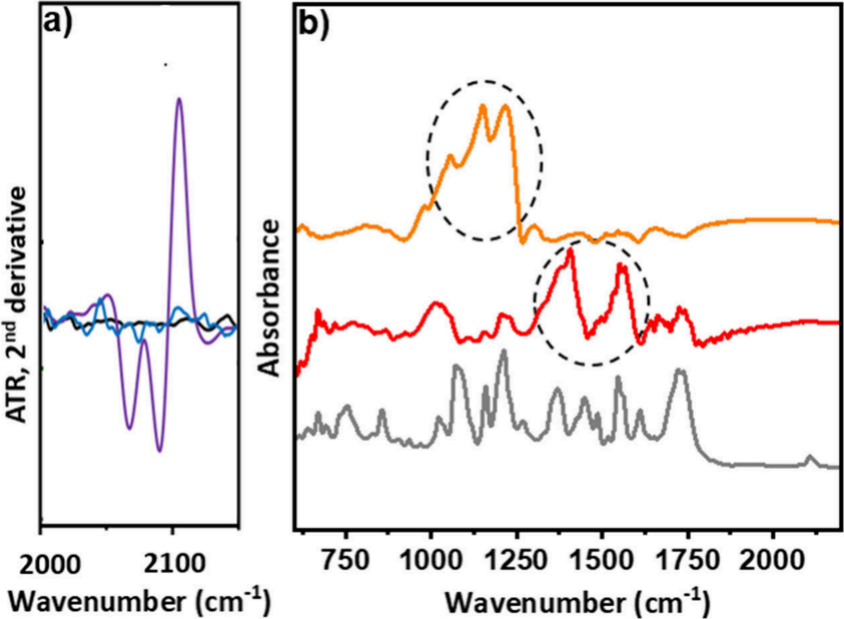
a) Second derivative of ATR-FTIR spectra focusing on the −N_3_ region: **1** (black), **3** (purple) and **5** (blue) and b) ATR-FTIR spectra of **4** (gray), **7** (orange) and **8** (red).

ATR-FTIR spectroscopy is a valuable tool for monitoring the progress of CuAAC click reactions.^48,49^ In the ATR-FTIR spectrum of **3** a distinct peak at 2096 cm^–1^ corresponding to the characteristic absorption band of -N_3_ groups is observed, while it is clearly absent in **1** (SI, Figure S15). Furthermore, the ATR-FTIR spectrum of the “clicked” derivative **5** (SI, Figures S16 and S17, Table S1) notably lacks both the characteristic −N_3_ bands of **3** and the −C≡CH bands of **4**, providing evidence for the formation of 1,2,3-triazoles in nanomaterial **5**. Moreover, the second derivative of the ATR-FTIR spectra, when comparing nanomaterials **1**, **3** and **5**, provides strong evidence for the completion of the CuAAC “click” reaction, showing the absence of the azide signature in the characteristic region 2000–2150 cm^–1^ (Figure 1a). Additionally, the ATR-FTIR spectra of both **7** and **8** exhibit characteristic fingerprint bands associated with ligand **4** (SI, Figure S16, Table S1), introduced onto MWCNTs (Figure 1b). The pronounced ‘in-ring’ aromatic stretch band at 1565 cm^–1^ (C–C) and of triazole (N = N) band at 1406 cm^–1^ are particularly noteworthy, suggesting a strong interaction between the PtNPs with both carbon framework and their proximal triazole moieties in **8**, with a simultaneous enhancement of the bands at 1374 cm^–1^ and 1549 cm^–1^ (SI, Table S2). In contrast, only the bands at 1066, 1147 and 1215 cm^–1^ are enhanced in **7**. These differences in the vibrational modes of **7** and **8** suggest substantial variations in their surface and electronic properties (SI, pp S37–S38).

**Figure 2:**
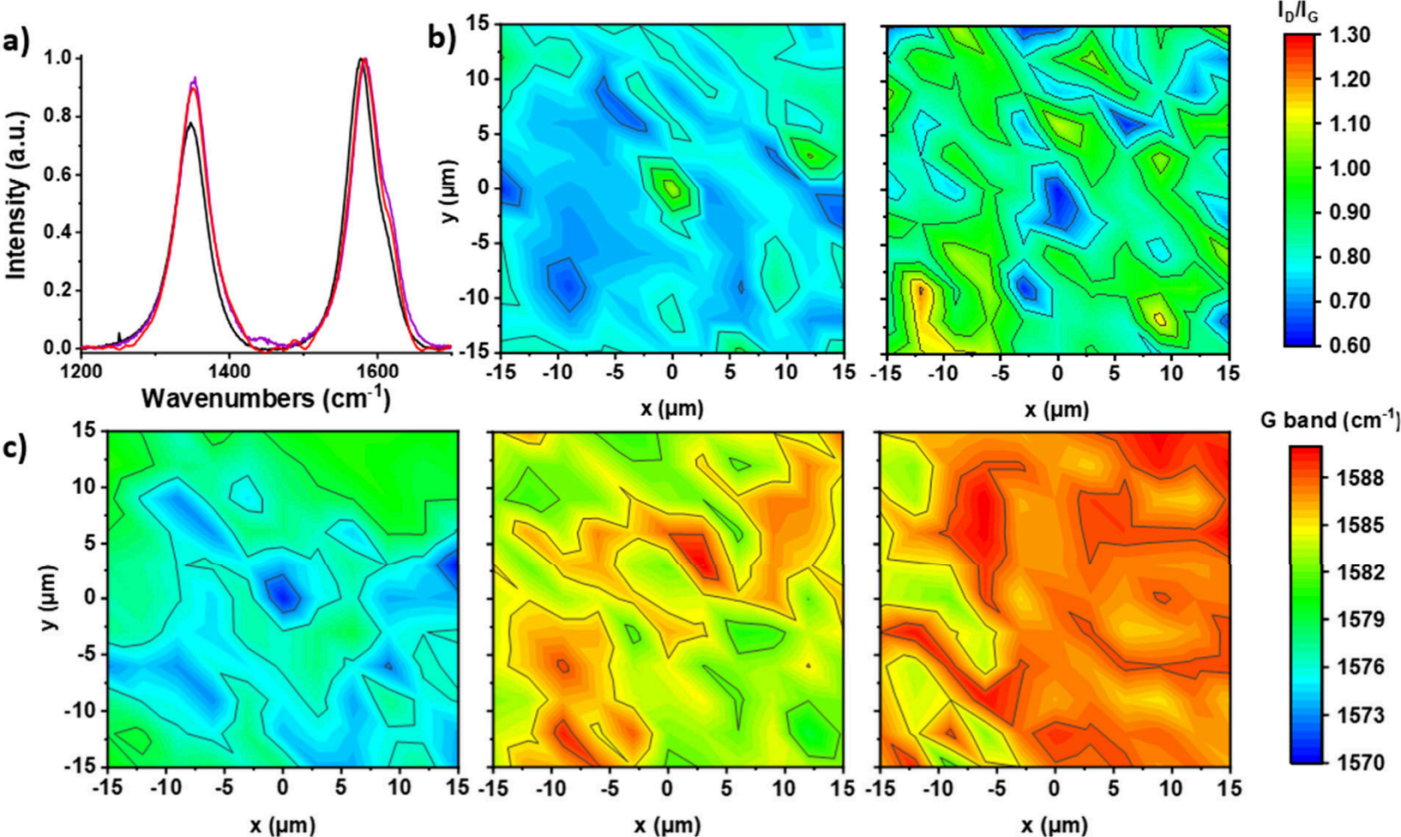
a) Raman spectra of **1** (black), **3** (purple) and **8** (red), b) Raman 30 μm × 30 μm spectral maps of I_D_/I_G_ ratio of **1** (left) and **8** (right), c) Raman 30 μm × 30 μm spectral maps of G position band of **1** (left), **8** (center) and **7** (right).

Raman spectroscopy offers valuable insights into the structural characteristics of MWCNTs nanomaterials. Raman measurements were taken on the powder samples of **1**, **3**, and **5–8**. All MWCNTs nanomaterials exhibit the characteristic D band, attributed to structural defects and disorder within the carbon nanotube lattice, as well as the G band, associated with the graphitic structure and crystallinity of the carbon nanotubes.^50,51^ Purified MWCNTs (**1**) have an intensity I_D_/I_G_ ratio of 0.80 ± 0,01 indicating an interplanar distance of 0.34 nm between graphite layers (SI, Figure S9a).^52^ The calculated I_D_/I_G_ ratio of MWCNTs-N_3_ (**3**) is 0.90 ± 0.01, suggesting an elevated presence of structural defects in the carbon lattice (Figure 2a and SI, Figure S13).^53^ This confirms the efficient covalent functionalization in **3** and subsequent partial transformation of sp^2^ to sp^3^ C atoms, due to the introduction of defects in the carbon framework. The I_D_/I_G_ ratio is maintained at 0.90 ± 0.01 (Table 1, Figure 2a-2b and SI, Figures S18a, S20a, S29a), validating that no further covalent modification occurs on the MWCNTs walls in nanomaterials **5–8**. The slight fluctuation in the observed I_D_/I_G_ ratio (0.89 – 0.91 ± 0.01) falls within the expected experimental uncertainty, arising from local sampling variations, and does not indicate any significant alteration in the MWCNT framework. However, the blue shifts observed in the peak positions of the D and G bands across nanomaterials **5–8** (as presented in Table 1) in comparison to purified MWCNTs **1**, signify notable alterations in terms of mechanical strain and electronic environment within the carbon nanoframework.^54−56^ Specifically, the D and G bands of nanomaterials **5** and **7** (Table 1 and SI, Figures S18b, c and S29b, c) exhibit significant and consistent blueshifts, approximately 4 cm^–1^ and 9 cm^–1^, as well as 5 cm^–1^ and 10 cm^–1^, respectively, gradually reflecting π-electron withdrawal and compressive strain induced by imidazolium chloride ligands. However, upon transforming imidazolium chloride in **5** into copper carbenes in **6**, the D and G bands undergo downshifts of approximately 2 cm^–1^ and 3 cm^–1^, respectively (Table 1 and SI, Figure S20b, c), corresponding to π-electron donation and lattice relaxation as the ligand evolves from an electron-poor imidazolium species to a more electron-rich NHC-Cu complex. In Figure 2c, Raman mapping illustrates shifts in the G band positions for MWCNTs **1**, control electrocatalyst **7**, and the advanced electrocatalyst **8**. It is also worth noting that electrocatalyst **8** shows consistent wavenumbers with **6** (Table 1), following the formation of PtNPs, suggesting a limited impact on the carbon framework upon the formation of PtNPs in electrocatalyst **8**. This is presumably due to the persistence of NHC-metal ligation following the transmetalation of **6**, thereby preventing any additional significant structural perturbation. Overall, the observed shifts in the peak positions of the D and G bands reflect substantial alterations of the carbon nanoframeworks and their electronic properties during the transition from purified MWCNTs **1** to advanced electrocatalyst **8** (Figure 2c, Table 1 and SI, Figure S24), simultaneously differentiating it from control electrocatalyst **7** (Figure 2c, Table 1 and SI, Figure S29b,c). Overall, these Raman shifts serve as a direct spectroscopic signature of the evolving charge transfer dynamics, ligand–metal coordination effects, and mechanical strain interplay, distinguishing electrocatalyst **8** from its precursors and control material **7**.

**Table 1:** 

Nanomaterial	I_D_/I_G_ ratio^a^	G shift (cm^–1^)^b^	D shift (cm^–1^)^b^
**1**	0.80	1577	1348
**5**	0.91	1586	1352
**6**	0.90	1583	1350
**7**	0.90	1587	1353
**8**	0.89	1584	1350

a I_D_/I_G_ average ratio (error bar = ± 0.01).

b G and D band average position for nanomaterials **1** and **5-8** (error bar = ± 1 cm^–1^).

**Figure 3:**
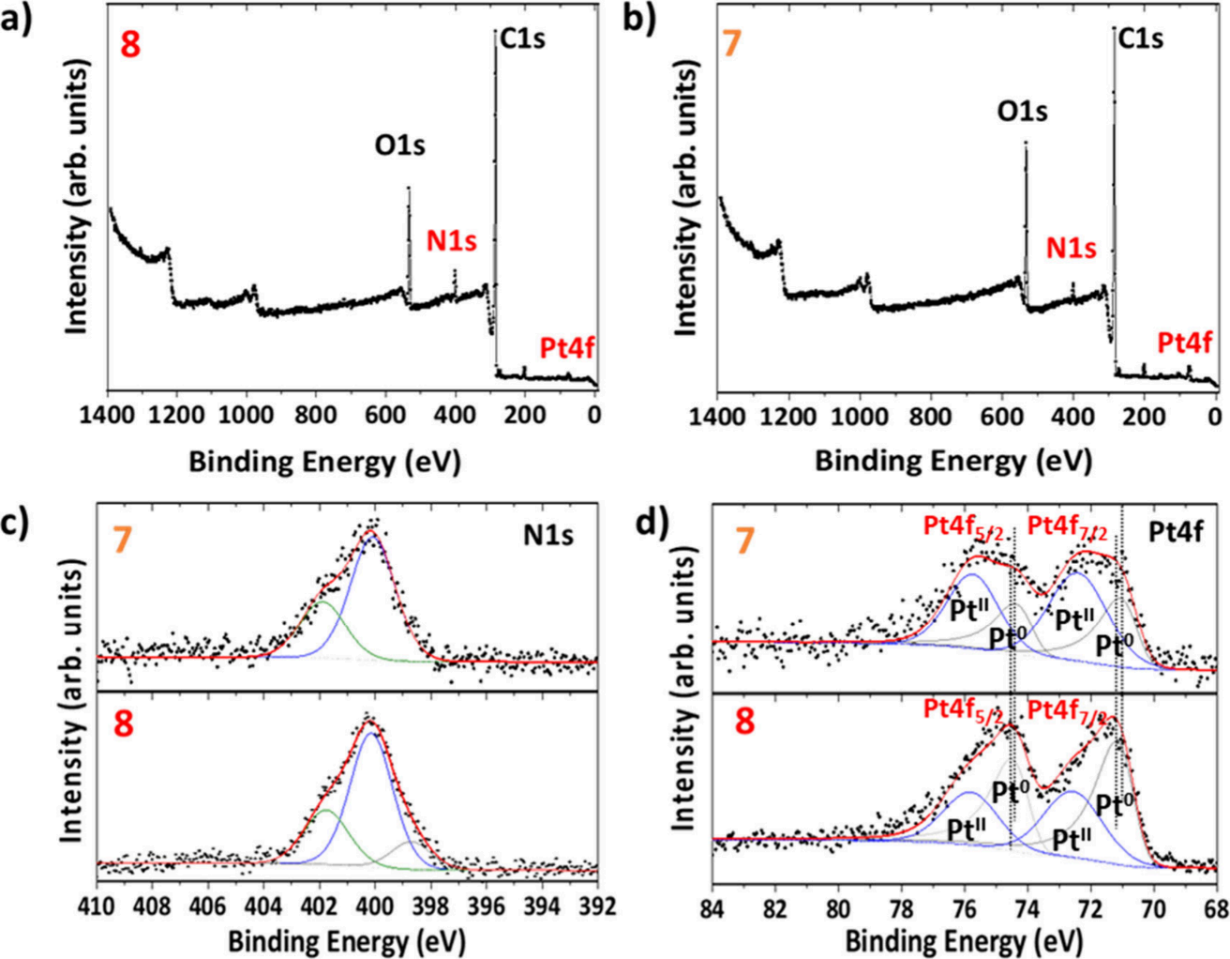
XPS Survey scan of a) **8** and b) **7**, XPS Narrow scan focusing on c) N 1s fitted peaks of **7** and **8** and d) Pt 4f fitted peaks of **7** and **8**.

X-ray Photoelectron Spectroscopy (XPS) provided additional, essential insights into the composition and electronic properties of nanomaterials **5**-**8**. The survey spectra accurately identified the presence of carbon (C), nitrogen (N) and oxygen (O) within nanomaterials **5**, **6, 8** and **7** (SI, Figures S19a, S21a and Figure 3a, b, respectively), confirming the composition of the desired nanostructures. The absence of copper in **5**, as shown by XPS, signifies the complete removal of the Cu (I) click reaction catalyst. On the other hand, copper was detected in **6**, confirming the success of the metalation reaction and our synthetic strategy (SI, Figure S21). The high-resolution N 1s spectra of nanomaterial **5** provide further strong support for the successful implementation of the CuAAC click reaction on the MWCNTs surface (SI, Figure S19c). This is evident from the absence of the characteristic −N=*N*=N– peak associated with the azide moiety, typically observed around 405 eV.^57^ In the same line, the N 1s photoelectron peak confirmed the existence of both imidazolium chlorides,^58^ and 1,2,3-triazole moieties.^59^ This consists of two distinct fitted components, one at 400.1 eV, corresponding to the −*N*–N=*N*– of 1,2,3-triazole and the **N** atom of imidazolium chloride and another at 401.8 eV, corresponding to the −N–*N*=N– of 1,2,3-triazole and the quaternary *N*^*+*^ of the imidazolium salt. The ratio of these two fitted peak areas in **5**, **6**, and **7** is 3:2, validating our synthetic route (Figure 3c and SI, Figures S19c and S21d). In the case of advanced electrocatalyst **8**, three components were clearly detected, at binding energies (BE) of 398.7, 400.1, and 401.8 eV (Figure 3c) in a ratio of 1:3:1. The appearance of the component observed at 398.7 eV is attributed to the Pt–C–*N* atom of the *N*-heterocyclic carbene, found at approximately 3.1 eV less energy compared to the *N*^*+*^ atom of its imidazolium salt control analogue **7**. This provides strong evidence for the significantly increased electron density around this N atom and is consonant with the conversion of the cationic imidazolium salts (with strongly bound electrons) into neutral, electron-richer carbene ligand species bonded to PtNPs.^60,61^ In the same line, high-resolution C 1s spectrum of **8** revealed a fitted component at 282.8 eV, attributed to the Pt–*C*–N. The Pt 4f spectra of **8** (Figure 3d) reveal a distinct Pt ligation electronic environment with a characteristic Pt (0) BE of Pt 4f_7/2_ found at 71.2 eV.^62^ Interestingly, the BE of Pt (0) in the final electrocatalyst **8** is 0.13 eV higher than that of control electrocatalyst **7**. This highlights the influence of the electronic environment and the π-accepting properties of the NHCs in **8**, that is, showing π-back-donation from Pt (0) atoms to the NHC scaffolds. Pt (II) was also detected in **8**, attributed to residual surface-bound Pt [δ+] atoms, as well as PtO oxidized species.^38^ It is important to note that the Pt (0)/Pt (II) normalized area ratio is 2-fold higher in electrocatalyst **8** than control electrocatalyst **7**,^62^ highlighting the protective role of NHC ligands in PtNPs against oxidation.^63^ The absence of Cu 2p detection in **8** confirms our notion for the nanoarchitecture of **8** on the MWCNTs carbon nanoframework (Figure 3a and SI, table in Figure S25), while Cu 2p, corresponding to metallic Cu (0) and CuO, were indeed detected in the byproducts (SI, pp S29 and Figure S26). Finally, PtNPs immobilized on MWCNTs were shown to contain only 0.4% Pt/C atomic ratio (SI, Figure S25). This reduced Pt loading in **8**, considering the scarcity and high cost of platinum, underscores its importance, given its high electrocatalytic efficiency in HER. In short, XPS results highlight the crucial role of NHC ligands in governing the PtNPs electronic environment, enhancing both stability and electrocatalytic performance.

**Figure 4:**
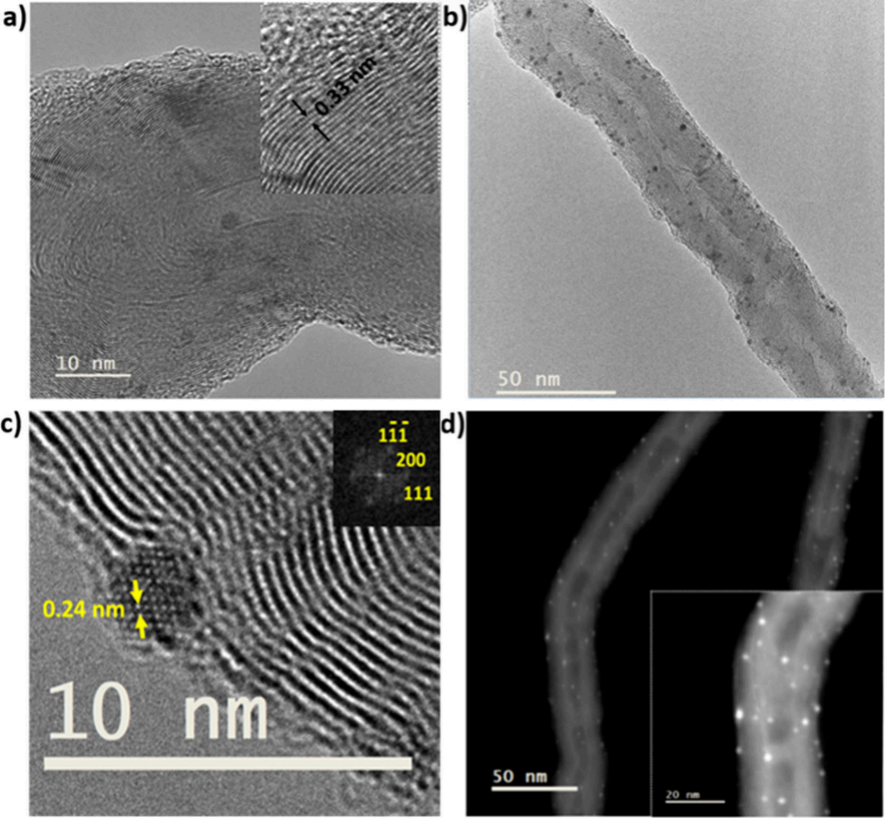
a) HRTEM micrograph of **8**(inset: interwall spacing of MWCNTs), b) TEM micrograph of **8**, c) HRTEM micrograph of **8** (inset: FFT), d) STEM-HAADF image of **8**.

Transmission Electron Microscopy (TEM) provided very important insights into the structural characteristics of electrocatalyst **8** as well. Low magnification TEM showed the presence of uniformly bent MWCNTs decorated with metal nanoparticles. The length of the MWCNTs is in the micrometer range, with an outer diameter found in the range of 30–40 nm and an inner diameter ca. 10 nm (SI, Figure S27a). High-resolution TEM (HRTEM) revealed the presence of well-ordered graphitic layers in MWCNTs, with an interwall spacing of 0.33 nm (Figure 4a), which is consistent with the findings of the Raman analysis of MWCNTs **1**. Immobilized within the MWCNTs, PtNPs exhibit remarkable uniformity in size distribution (Figure 4b), with diameters ranging from 1.5 to 2.7 nm and a mean diameter (d) of 2.0 ± 0.3 nm (SI, Figure S27b). Atomic resolved HRTEM images show the highly crystalline nature of the face-centered cubic (fcc) PtNPs along the [0–11] direction (Figure 4c). Notably, the observed periodicities in the atomic resolved nanoparticles and their corresponding FFT fit well with fcc Pt along the [0–11] zone axis. These observations reveal that nanoparticles exhibit a strongly preferred orientation along the {1 1 1} crystal plane, thus promoting enhanced electrocatalytic activity and stability against corrosion.^16,20,21,63^ The number of Pt atoms of the PtNPs in Figure 4c with d = 2.3 nm is 418, calculated using the Rhodius software.^64^ HRTEM micrographs of PtNPs situated at the edges of MWCNTs allowed us to detect an amorphous surrounding that can be attributed to the presence of the NHC ligands (Figure 4c). Scanning Transmission Electron Microscopy (STEM) coupled with High-Angle Annular Dark-Field (HAADF) imaging was also employed, to precisely locate the metal nanoparticles within the carbon nanocarrier based on their higher atomic number (Figure 4d), revealing the exceptional spatial distribution and dispersion of PtNPs within the MWCNTs. Additionally, energy-dispersive X-ray spectroscopy (EDS) analysis further confirmed the absence of copper while exclusively validating the presence of carbon and platinum elements, attesting to the pure Pt composition of the nanoparticles (SI, Figure S28). The exceptional distribution, within the MWCNTs nanocarrier, and surface-to-volume ratio of these ultrasmall PtNPs, coupled with their {1 1 1} faceted crystalline structure, offers substantial advantages in enhancing electrocatalytic activity for HER in the electrocatalyst **8**. Control electrocatalyst **7** was also examined, to rationalize its inferior activity. Indeed, its structural characteristics were markedly different: TEM and HRTEM micrographs of **7** revealed the presence of MWCNTs with larger Pt nanoparticles, along with extensive aggregates (SI, Figures S31 and S32), thus leading to significantly reduced electrocatalytic activity and stability, compared to electrocatalyst **8**.

**Figure 5:**
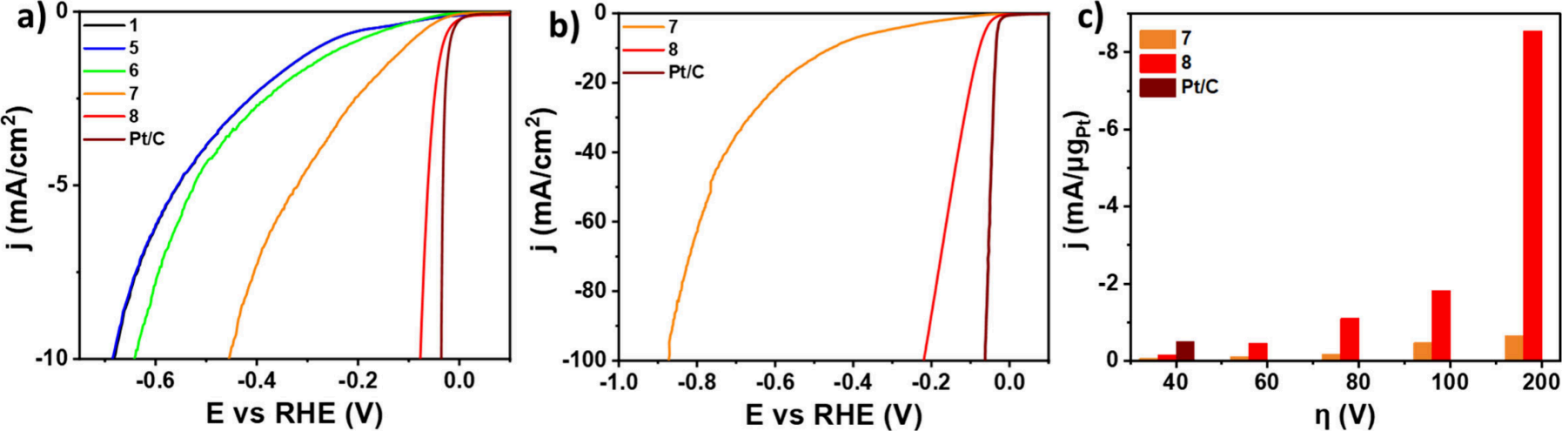
a) LSV polarization curves of **1, 5–8** and commercial **Pt/C** (20% wt. on carbon) recorded up to j = -10 mA/cm^2^. b) LSV polarization curves of **7, 8** and commercial **Pt/C** (20% wt. on carbon) recorded up to j = -100 mA/cm^2^. c) Current densities normalized Pt mass at different potentials for **7, 8** and commercial **Pt/C** (20% wt. on carbon). All LSVs were recorded at 1600 rpm rotation speed and 5 mV/s scan rate.

The electrocatalytic activity toward HER was investigated employing electrocatalyst **8**, control electrocatalyst **7**, and all related reference nanomaterials, by using the linear sweep voltammetry (LSV) technique, and the results are summarized in Table 2. LSV experiments were conducted with a glassy carbon (GC), rotating-disc electrode (RDE) as the working electrode in a standard three-electrode cell, with a scan rate of 5 mV s^–1^ in N_2_-saturated 0.5 M H_2_SO_4_ electrolyte. Polarization curves for electrocatalyst **8**, control electrocatalyst **7**, and the related reference nanomaterials **1**, **5**, and **6** were obtained, revealing an exceptional electrocatalytic activity of **8**. Compared to the commercial, state-of-the-art, Pt-based electrocatalyst (20% wt. on carbon), nanohybrid **8**, with only 0.4% Pt atomic loading on the nanocarrier’s carbon, demonstrates a similar onset potential (E_ons_) for HER, whereas its potential at the current density of −10 mA/cm^2^ (E_10_), is only 43 mV greater, appearing at −0.077 V vs RHE. Impressively, the E_10_ of **8** is found to be lower by 599, 606, and 560 mV compared to reference materials **1**, **5**, and **6**, respectively, highlighting its superior electrocatalytic performance (Figure 5a). The enhanced electrocatalytic activity of **8** is governed by the direct and indirect impact of the NHC-PtNPs ligation. Control electrocatalyst **7** shows a certain electrocatalytic activity against HER as well; however, its E_10_ appears at −0.453 V, 376 mV and 419 mV greater than **8** and commercial **Pt/C** (20% wt. on carbon), respectively. The E_100_ values further highlight the strong performance of electrode **8**, which exhibits an overpotential of 217 mV (Figure 5b). In contrast, electrode **7** shows a significantly higher overpotential of 870 mV at −100 mA/cm^2^. This comparison unequivocally demonstrates that the reliance on triazole ligation alone, even when enhanced by additional electrostatic interactions with Pt nanoparticles (via imidazolium chlorides) in **7**,^65^ falls short of achieving the remarkable performance seen in our advanced nanoelectrocatalyst **8**. Overall, the comparison of **8** with precursors **1**, **5**, **6** and control electrocatalyst **7**, conclusively establishes that neither MWCNT defects nor non-NHC-ligated PtNPs contribute to HER enhancement. Instead, the exceptional electrocatalytic performance of **8** arises from PtNPs strongly ligated via Pt–C–N bonding on the ligands attached to MWCNTs, as evidenced by comparative Raman analysis and HER activity trends across the entire nanomaterial series (**1** and **5–8**).

**Table 2:** Summary of Electrocatalytic HER Parameters in N_2_-Saturated 0.5 M H_2_SO_4_ Electrolyte for **8** in Comparison with Nanomaterials **1** and **5–7** as well as **commercial Pt/C** (20% wt. on Carbon).

Nanomaterial	E_ons_ vs RHE (V)	E_10_ vs RHE (V)	E_100_ vs RHE (V)	E_10_ after 10^4^ scans vs RHE (V)	Tafel slope (mV/dec)	Rct (Ω)
**1**	–0.220	–0.676	–	–0.693	434	983
**5**	–0.220	–0.683	–	–0.622	436	793
**6**	–0.186	–0.637	–	–0.655	432	622
**7**	–0.033	–0.453	–0.870	–0.477	191	281
**8**	–0.005	–0.077	–0.217	–0.085	50	90
**Pt/C**	–0.003	–0.034	–0.064	–0.030	19	6

The Pt mass loadings of the advanced electrocatalyst **8** and the control electrocatalyst **7** are estimated to be 10.6 μg_Pt_ and 3.9 μg_Pt_, respectively, based on the Pt/C ratio of 0.4% in **8** and 0.2% in **7** as found in XPS measurements (Figure S25 and Figure S30), which provide a quantitative chemical composition of a material surface within 10 nm.^66^ The normalized, *iR*-compensated LSV curves, recorded up to −100 mA/cm^2^ with respect to the Pt loading (Figure S33), indicate that **8** exhibits a mass activity of 1.8 A/mg_Pt_ at η = 100 mV and 8.6 A/mg_Pt_ at η = 200 mV, highlighting the effective utilization of Pt in **8**, underpinned by its unique nanostructure and favorable surface interactions (Figure 5c). Interestingly, the mass activity of **8** surpasses that of **7** by a factor of 4 at 60 mV whereas at 200 mV, the mass activity of **8** is 13-fold higher than that of **7.**

The superior performance of **8** can be attributed to several key factors: First, NHC-Pt covalent bonding plays a crucial role, enhancing stability,^38^ preventing aggregation,^28−30^ and mitigating irreversible Pt oxidation,^44^ thus ensuring sustained performance. Additionally, the PtNPs crystalline structure, with a preferred orientation along the {1 1 1} crystal plane, provides a highly active catalytic surface due to the high density of active sites in **8**, leading to an optimal balance between adsorption/desorption of H on the {1 1 1} facets. Finally, the uniform dispersion of small-sized {1 1 1} faceted PtNPs on the MWCNTs, coupled with the high surface-to-volume ratio resulting from both PtNPs and MWCNTs nanocarrier, contribute to enhanced catalytic activity and efficient mass transport. This surface arrangement provides a larger number of electroactive sites and facilitates the diffusion of reactants, thus resulting in faster HER kinetics. In contrast, control electrocatalyst **7** exhibits larger nanoparticles, lack of uniformity, polydispersion, extensive aggregation, and weaker supramolecular stabilization, resulting in the observed overall inferior performance.

**Figure 6:**
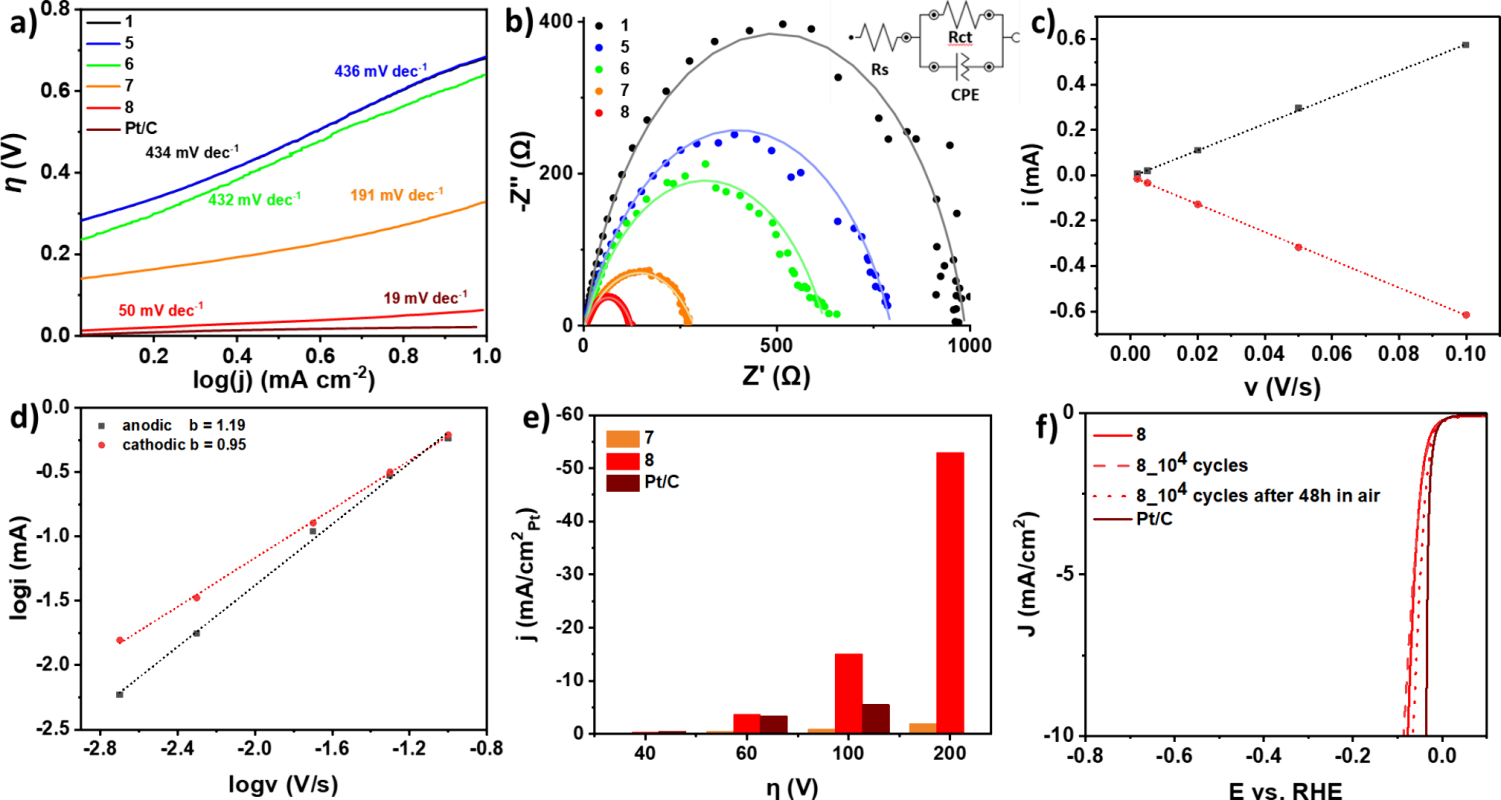
a) Tafel Slopes and tabulated values for **1, 5–8** and commercial **Pt/C** (20% wt. on carbon). b) Experimental Nyquist plots (dots) fitted (solid lines) with equivalent Randles circuit (inset) for **1** and **5–8**. c) Linear Fits of i (mA) vs v (V/s) plot with R^2^_cathodic_ (red) and R^2^_anodic_ (black) values of 0.99. d) Linear fits of logi (mA) vs logv (V/s) with calculated cathodic and anodic slopes (b). e) Current densities normalized Pt surface (cm^2^) at different potentials for **7, 8** and commercial **Pt/C** (20% wt. on carbon). f) LSV polarization curves of **8** (red solid line), after 10^4^ cycles (red dashed line) and its subsequent exposure in air for 48 h (red dotted line) and commercial Pt/C (20% on carbon) (wine line), obtained at 1600 rpm rotation speed and 5 mV s^–1^ scan rate.

Valuable insights into the reaction kinetics and mechanism were obtained by analyzing the Tafel slopes, extracted from LSV polarization curves, charge transfer resistance values (R_ct_) obtained from electrochemical impedance spectroscopy (EIS) assays and Cyclic Voltammetry (CV) measurements, once more demonstrating the superior activity of **8**. The Tafel slope value for nanohybrid **8** was found to be 50 mV/dec, revealing that it follows the Volmer–Heyrovsky path, where the rate-limiting step is the electrochemical desorption of adsorbed hydrogen atoms from the modified electrode surface (Heyrovsky step). On the other hand, **7** exhibits a much higher Tafel slope (191 mV/dec), leading to a hydrogen adsorption rate-limiting step on the electrode surface (Volmer step), thus resulting in slower reaction kinetics (Figure 6a). Further insights into HER kinetics were provided from EIS, particularly the **R**_**ct**_ at the electrode–electrolyte interface. Experimental Nyquist plots fitted with Randles circuit (Figure 6b and SI, Figure S34 and Table S4) show that nanohybrid **8** demonstrates the smallest frequency semicircle, suggesting the lowest R_ct_ value (R_ct_ = 90 Ω), therefore exhibiting enhanced charge transfer, compared to control electrocatalyst **7** (R_ct_ = 281 Ω), as well as reference nanomaterials, **1** (R_ct_ = 983 Ω), **5** (R_ct_ = 793 Ω), and **6** (R_ct_ = 622 Ω). The tailored nanoarchitecture of **8**, involving the covalent linkage of MWCNTs with NHC ligands and their covalent robust attachment with {1 1 1} faceted PtNPs, facilitates higher conductivity and improved accessibility for charge transfer, thus leading to significantly more efficient electrocatalytic HER performance. CV measurements in 0.5 M H_2_SO_4_ reveal characteristic redox waves for hydrogen (H_2_) desorption/adsorption and the irreversible PtO redox wave in both **Pt/C** and **7**, although the latter exhibits substantially lower currents (SI, Figure S35). In contrast, the CV of **8** displays featureless H_2_ desorption/adsorption, but presents a distinct, chemically reversible PtO/Pt redox wave, which remains reversible on the electrochemical time scale (SI, Figure S36). The strong linearity of both anodic (i_a_) and cathodic (i_c_) currents with the scan rate (*v*) (R^2^ = 0.99 for both) highlight that the redox process is predominantly surface-controlled (Figure 6c). Additionally, the linear correlation of both anodic (i_a_) and cathodic (i_c_) currents with √v (R^2^ = 0.96 for both), suggests that the redox kinetics also exhibit diffusion-controlled characteristics (SI, Figure S37).^67^ This dual control illustrates the intricate balance between proton diffusion and adsorption in determining the electrochemical behavior of **8** during HER.^68^

To gain deeper insight, the dynamic relationship between the scan rate (*v*) and current (i): i = α*v*^b^, where α and b are constants, was further explored.^69^ The b values derived from the linear slopes of log(i) versus log(v) plots were found to be b = 0.95 for the oxidation peak and b = 1.19 for the reduction peak. These values further elucidate the surface-controlled PtO/Pt redox process and reveal capacitive characteristics at the surface of electrocatalyst **8** (Figure 6d).^70^ This pseudocapacitive behavior provides an electron-rich surface, facilitating proton reduction coupled with PtO/Pt redox cycling during the Heyrovsky step of HER, leading to the reduction of PtO to Pt (0) and restoring active Pt sites for continuous HER. Furthermore, the high Y_0_ value of 86 μS.s^N^, with a nonideal CPE exponent of N = 0.6, obtained from Nyquist plot analysis of electrode **8** (SI, Table S4), confirms pseudocapacitive charge storage governed by redox processes at the electrode–electrolyte interface. The PtO/Pt redox cycling stabilizes the electron-rich electrode surface and enhances overall reaction kinetics, showcasing the robustness of the NHC-Pt (0) bond in electrocatalyst **8**.

Following the findings presented, the specific activity was evaluated to gain a deeper understanding of the efficiency and intrinsic properties of individual Pt electroactive sites. This specific activity was calculated from the normalized, *iR*-compensated LSV curves in relation to the electroactive surface area (ESCA) of electrocatalysts **7**, **8**, and **Pt/C** (SI, Figure S38). Given the differing redox characteristics between **7**, **8** and **Pt/C**, the ESCA was estimated by CV measurements in nonfaradaic regions at various scan rates for all materials (SI, Figures S39–S43). At an overpotential of 100 mV, the specific activity of electrocatalyst **8** is 15 mA/cm^2^_Pt_, which is 18-fold higher than that of control electrocatalyst **7** and 3-fold higher than **Pt/C**. Notably, at 200 mV, this specific activity significantly increases to 53 mA/cm^2^_Pt_ (Figure 6e). These results underscore the remarkable efficiency of each Pt site within the nanostructure of electrocatalyst **8** across varying overpotentials.

Last, stability and durability are key aspects for practical electrocatalyst applications, so these were also thoroughly investigated for both **8** and **7**, as well as for all related nanomaterials (SI, Figure S44, Table S5). The LSV measurements were performed before and after a) 10^4^ cycles and b) exposing the modified electrode with **8**, after 10^4^ cycles, to air for 48 h (Figure 6f). Remarkably, the LSV curves of **8**, in both testing cases, demonstrated no significant current loss, highlighting its exceptional stability. In contrast, control electrocatalyst **7** exhibited a 24 mV greater **E**_**10**_ value, after 10^4^ repetitive cycles, maintaining its inferior performance (SI, Figure S45).

Detailed HER parameters for all nanomaterials and commercial **Pt/C** (20% wt. on carbon), measured under the same conditions, are summarized in Table 2, while a comparison table of low-loading Pt-based electrocatalysts is provided in SI, Table S6.

## Conclusions

Our work introduces a strategically designed nanoarchitecture to address critical challenges in the field of advanced electrocatalysis for HER. This innovative approach combines MWCNTs covalently decorated with NHC ligands, which, in turn, form robust bonds, through transmetalation, with highly crystalline, {1 1 1}-faceted, ultrasmall (∼ 2 nm) PtNPs. With a Pt/C atomic content of only 0.4%, as determined by XPS, our novel electrocatalyst **8** shows a minimal overpotential of 77 mV at −10 mA/cm^2^ and 217 mV at −100 mA/cm^2^ at and fast reaction kinetics with an R_ct_ markedly lower than reference materials and a Tafel slope of 50 mV/dec. The mass activity and specific activity at 200 mV is 8.6 A/mg_Pt_ and 53 mA/cm^2^_Pt_, respectively, highlighting the catalytic efficiency of each Pt site involved in **8**. CV measurements revealed a distinct redox behavior, characterized by featureless H_2_ redox waves and a prominent reversible PtO/Pt redox wave. The comprehensive analysis of the current versus scan rate relationship highlights the interplay between adsorption and diffusion, demonstrating surface-controlled kinetics supported by pseudocapacitive behavior, facilitating an electron-rich surface which enhances proton reduction in electrocatalyst **8**, particularly during the rate-limiting Heyrovsky step of the HER.

Additionally, even after enduring 10^4^ scans of cycling and exposure to air, it retains its stability and durability, presenting no loss of current. The exceptional electrocatalytic activity of **8** is attributed to its unique nanoarchitecture, combining a robust nanocarrier with NHC-PtNPs ligation, which prevents aggregation, enhances stability, and mitigates irreversible Pt oxidation. Moreover, the uniform dispersion and distribution of small-sized PtNPs on the MWCNTs carbon framework, coupled with the resultant high surface-to-volume ratio of electroactive sites, facilitate efficient charge-transfer processes, thus leading to faster HER kinetics. The PtNPs crystalline structure, exposing {1 1 1} crystal planes promoted by NHC-Pt (0) ligation, boosts both the electrocatalytic efficiency and reaction kinetics, due to its superior resistance against surface rearrangement and dissolution.

Its comparison with the control electrocatalyst (**7**), which deliberately lacks the NHC-Pt (0) ligation, shows the pivotal role of NHC-PtNPs bonding in promoting HER reaction. A comprehensive analysis of the distinctive variations in surface and electronic properties between **7** and **8** was conducted through FTIR, Raman, and XPS spectroscopies. The transformative potential of our platform not only deepens our understanding of electrocatalysis but also paves the way for “greener” and more efficient energy conversion technologies, therefore contributing to a more sustainable future.

## Methods

### Chemicals and Reagents

All chemicals and materials used in this study were of analytical grade and were obtained from reputable manufacturers. Sulfuric acid (H_2_SO_4_, 95–98% purity, Sigma-Aldrich) was used as the electrolyte for electrochemical experiments. Potassium hydroxide (KOH, 85% purity, Fisher Scientific) was employed in the synthesis of the catalysts. Platinum(II) chloride (PtCl_2_, ≥ 99% purity, Alfa Aesar) was utilized for the preparation of the platinum nanoparticles. The synthesized ligand, 1-mesityl-3-(4-((prop-2-yn-1-yloxy) methyl) benzyl)-1H-imidazol-3-ium chloride, was prepared according to established procedures. Multiwalled carbon nanotubes (MWCNTs, ≥95% purity, obtained from Nanostructured & Amorphous Materials, Inc.) served as the support material for the catalysts. All reactants and reagents were obtained from commercial suppliers and used as received unless otherwise stated. Aligned multiwalled carbon nanotubes/MWCNTs (d = 10 nm, CNTs >95%) used were purchased from NanoAmor (Los Alamos, NM, USA). The course of the organic reactions was monitored via thin-layer chromatography (TLC), using aluminum sheets (0.2 mm) coated with silica gel 60 with a fluorescence indicator (silica gel 60 F_254_). The developed TLC plates were analyzed by a UV lamp (254 and 365 nm) or by potassium permanganate solution for visualization. Purification of the products was carried out by flash column chromatography, using silica gel 60 (230–400 mesh). Pt on graphitized carbon was purchased from Aldrich with 20 wt % loading.

**Proton nuclear magnetic resonance spectra (**^**1**^**H NMR)** were obtained with a Bruker Avance 400 MHz or a Varian Mercury 200 MHz spectrometer, using CDCl_3_ as solvent and its residual solvent peak as a reference. Data are reported as follows: chemical shift, multiplicity (s = singlet, d = doublet, t = triplet, m = multiplet), coupling constant (Hz), and integration.

**High-resolution mass spectra (HRMS)** were recorded in a QTOF maxis impact (Bruker) spectrometer with electron spray ionization (SI).

**Probe sonication** was performed with a Bandelin Sonopuls Ultrasonic Homogenizer HD 3200 equipped with a flat head probe (VS-70T), running at 35% (87.5 W) of the maximum power (250 W).

**Midinfrared spectra** in the 675–4500 cm^–1^ region were acquired on a Fourier transform IR spectrometer (Tensor II, Bruker Optics) equipped with a single reflection Ge ATR crystal (Miracle by PIKE Technologies). Typically, 100 scans were acquired at 4 cm^–1^ resolution.

**Micro-Raman scattering** measurements were performed employing compressed powder samples, at room temperature, in the backscattering geometry using a RENISHAW inVia Reflex Raman spectrometer equipped with a CCD camera and a Leica microscope. A 2400 lines mm^–1^ grating was used for all measurements, providing a spectral resolution of ± 1 cm^–1^. As the excitation source, an Ar^+^ laser line of 514 nm was used. Measurements were taken with varying numbers of accumulations, each with a 10 s exposure and laser power ∼0.3 mW cm^–2^ to avoid overheating of the samples. The laser spot was focused on the sample surface using a long working distance Leica 50x objective lens. Raman spectra were collected after map image acquisition was conducted on various (3–5) areas of the sample and recorded with Peltier cooled CCD camera. The intensity ratio D/G was obtained by taking the integrated peak intensities, following baseline correction. For the mapping recordings: 5 areas (30 μm x 30 μm each) were measured with a 3 μm step. for every sample, and we include the most representative map, closest to the total average with respect either to the intensity ratio D/G, or the G and D bands position. The data were collected and analyzed with Renishaw Wire 3.4 and Origin 8 Pro software. **Thermogravimetric analysis** was performed using a TGA Q500 V20.2 Build 27 instrument by TA in an inert atmosphere of nitrogen (purity >99.999%). In a typical experiment, 2 mg of the material were placed in the platinum pan and the temperature was equilibrated at 40 °C. Subsequently, the temperature was increased to 800 °C at a rate of 10 °C/min and the mass changes were recorded as a function of temperature. Derivatives of mass loss to temperature were used to assess the thermal decomposition profile of each hybrid material.

**XPS measurements** were performed under Ultra High Vacuum conditions (UHV, with a base pressure of 4 × 10^–10^ mbar), using a monochromatic Al Kα line as exciting photon source for core level analysis (hν = 1486.7 eV). The emitted photoelectrons were collected in a hemispherical energy analyzer (SPHERA-U7, pass energy set to 20 eV for the XPS measurements to have a resolution of 0.6 eV) and to compensate for the built-up charge on the sample surface it was necessary (for the XPS measurements) the use of a Flood Gun (FG-500, Specs), with low energy electrons of 3 eV and 40 μA. The sp2 component of the C 1s core level centered in 284.4 eV was used as a binding energy reference.

**Electron Microscopy.** Samples for electron microscopy were deposited on carbon-coated nickel grids by drop-casting butanol suspensions of functionalized MWCNTs. The **Transmission Electron Microscopy (TEM)** study at low magnification was performed with a JEOL 2100 transmission electron microscope operated at 200 kV. **High Resolution Transmission Electron Microscopy (HRTEM)** images were obtained in a JEOL-JEM GRAND ARM 300cF microscope equipped with a Cs Corrector (ETA-JEOL). A precise measurement of the aberrations and an optimized correction has been done using the corrector control software JEOL COSMO. The accelerating voltage was set to 60 kV in order to minimize the sample damage. The HRTEM images were acquired by a Gatan OneView camera (4096 × 4096 pixels). **EDS** spectra were acquired in STEM mode using a SDD CENTURIO detector.

**Hydrogen evolution reaction (HER)** measurements were carried out on an AUTOLAB PGSTAT128N (Metrohm) potentiostat/galvanostat in a standard three-compartment electrochemical cell. A graphite rod was used as the counter-electrode, a glassy carbon (GC), ring rotating disk electrode (RRDE) with geometric surface area **A**_**Geom**_ = 0.196 cm^2^ was used as the working electrode and a mercury sulfate electrode (MSE) (Hg/HgSO_4_, saturated K_2_SO_4_ as filling solution) were used as the reference electrodes for studies in acidic conditions. LSV polarization curves were recorded at ambient temperature in freshly prepared, N_2_-saturated aqueous 0.5 M H_2_SO_4_ (pH = 0.3, acidic conditions) at a scan rate of 5 mV s^–1^. The catalyst ink was prepared by dispersing 1.0 mg of the catalytic powder in a 250 μL mixture of distilled water, isopropanol, and 5% Nafion (v/v/v = 4:1:0.02) and bath sonicated for 30 min prior to use. Before casting the electrocatalytic ink on the electrode surface, the working electrode was polished with 1 mm diamond paste and fine Al_2_O_3_ powder and thoroughly rinsed with distilled water. Afterward, an 8.5 μL aliquot of the electrocatalytic ink was drop casted on the electrode surface and was first left to dry at ambient temperature, then under N_2_ stream. Total catalyst loading on the GC-RRDE was ∼173 μg cm^–2^. The Pt/C electrode was prepared also as described above. The platinum loading on the Pt/C electrode was approximately 34.6 μgPt cm^–2^.

All measured potential values were referenced to the reversible hydrogen electrode (RHE) using the following eq 1:
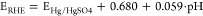
1

To account for Ohmic resistance in the electrochemical measurements, iRs compensation was applied at 100% for all LSV measurements using eq 2:
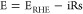
2where E is the iRs-corrected potential, E_RHE_ is the measured potential relative to the RHE, i is the measured current, and Rs is the uncompensated solution resistance determined via EIS. EIS was conducted over a frequency range of 10^5^ to 10^–1^ Hz with an AC amplitude of 0.01 V. The uncompensated resistance (Rs) was extracted from the Nyquist plot, where the real part of the impedance was assessed at the imaginary part being zero. For enhanced accuracy, the impedance spectrum was fitted to a Randles circuit model, yielding precise values for Rs. The EIS experiments were performed at j = −2 mA/cm^2^ for **1**, **5–8** and **Pt/C**. All Rs values are detailed in Table S4. No distortions or instabilities were observed in LSV plots after 100% iRs correction, confirming the reliability of the compensation approach.

For the calculation of double layer capacitance (**C**_**dl**_), cyclic voltammograms were recorded at a 300–400 mV window in a region where no Faradaic processes take place and with scan rates ranging from 50 to 500 mV s^–1^. All CV measurements were conducted after electrode stabilization through multiple CV cycles to ensure steady-state behavior before data collection. It is known that charging current (*i*_*c*_) is directly proportional to the scan rate (*v*), with the slope (b) corresponding to **C**_**dl**_ (*i*_*c*_ = b · *v*, see SI, ref. S4). Therefore, the 1/2 Δ*i* (Δ*i* = *i*_anode_ – *i*_cathode_) was plotted against the scan rate and an average value of **C**_**dl**_ was calculated. For the calculation of electrochemically active surface area (**ECSA**), the roughness factor (**RF**) should initially be estimated. By taking into consideration a 40 μF cm^–2^ value for the specific capacitance of a flat electrode (**C**_**s**_), roughness factor was calculated by the eq 3 (see SI, ref. S5):
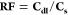
3

Finally, ECSA was calculated via eq 4:
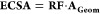
4

Minor fluctuations in capacitive current were observed across all nanomaterials, which is expected for nanostructured electrodes due to localized charge redistribution and interfacial dynamics. However, all ECSA values remained consistent upon remeasurements, confirming the reliability of the approach. The linear correlation of 1/2 Δi vs scan rate plots (Figures S39–S43, insets) reinforces the accuracy of capacitance-based ECSA estimation despite minor variations.

The cycling stability test was performed at a 50 mV/s scan rate. The potential was cycled for 10^4^ times between the values corresponding to current densities of 0 mA/cm^2^ and −10 mA/cm^2^ for each catalyst (SI, pp S46).

## Supporting Information Available

Full experimental procedures and data, ^1^H and ^13^C NMR, ATR-FTIR, Raman, and XPS spectra, TGA thermographs, HRTEM and TEM analyses data, and electrocatalytic data. (PDF)
