# Supplementary material for: Nanoarchitectured N-Heterocyclic Carbene-Pt Nanoparticles on Carbon Nanotubes: Toward Advanced Electrocatalysis in the Hydrogen Evolution Reaction
Source: ACS Appl Mater Interfaces. 2025 Mar 13;17(19):28138–50. doi: 10.1021/acsami.5c02182 (PMC12086845; doi:10.1021/acsami.5c02182)
Supplement: Supplementary file 1 — am5c02182_si_001.pdf [file am5c02182_si_001.pdf]

SUPPORTING INFORMATION

Nanoarchitectured *N*-Heterocyclic Carbene-Pt Nanoparticles on Carbon Nanotubes: Toward Advanced Electrocatalysis in the Hydrogen Evolution Reaction

Amalia Rapakousiou*[,†], Michail P. Minadakis[†], Savvas G. Chalkidis[‡], María Luisa Ruiz-González§, Cristina Navio[‖], Georgios C. Vougioukalakis*[,‡], Nikos Tagmatarchis*[,†]

†Theoretical and Physical Chemistry Institute, National Hellenic Research Foundation, 48 Vassileos Constantinou Avenue, Athens 11635, Greece

‡Laboratory of Organic Chemistry, Department of Chemistry, National and Kapodistrian University of Athens, 15771, Athens, Greece

§Departamento de Química Inorgánica, Universidad Complutense de Madrid, Madrid, Spain

‖IMDEA Nanoscience, C/Faraday 9, Ciudad Universitaria de Cantoblanco, 28049 Madrid, Spain

Corresponding Author

E-mail: arapak@eie.gr

1. Experimental Procedures & Characterization

1.1 Synthesis of ligand 4

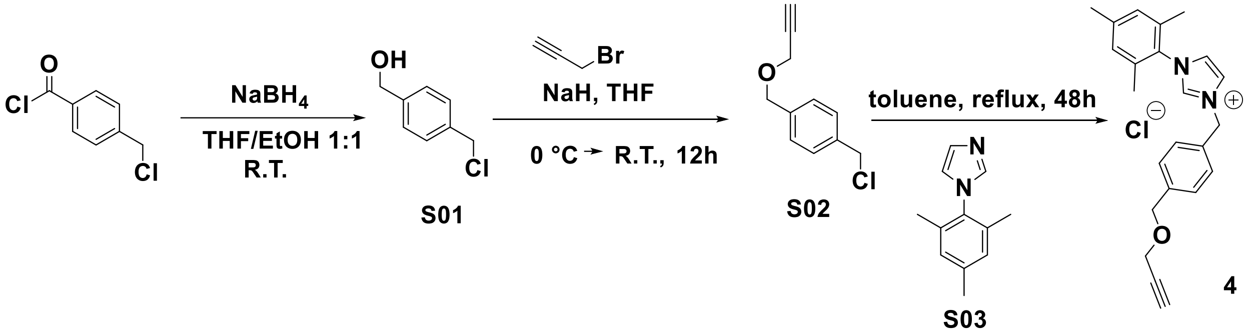

Scheme S1. Synthesis of ligand **4**.

1.2.1 (4-(chloromethyl)phenyl)methanol (S01)

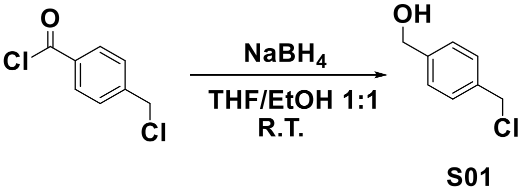

A round bottom flask was charged with 4-(chloromethyl)benzoyl chloride (1.34 g, 6 mmol, 1 eq.) and THF:EtOH (100 mL, 1:1) mixture. Then, $NaBH_4$ (0.91 g, 24 mmol, 4 eq.) was added in portions and the reaction was stirred under argon at room temperature for 2 h. The solvent was removed under reduced pressure. EtOAc (100 mL) was added and washed with 0.3 M $NaHCO_3$ (100 mL). The phases were separated, and the organic layer was dried over Na_2SO_4, filtrated and concentrated. The crude product was recrystallized from *n*-hexane to afford (4-(chloromethyl)phenyl)methanol as colorless crystals (695 mg, 74%). The spectral data are in accordance with the literature.[2]

^{1}H-NMR (400 MHz, $CDCl_3$): δ 7.42 – 7.33 (m, 4H), 4.70 (d, J = 6.0 Hz, 2H), 4.59 (s, 2H), 1.70 (s, 1H). **^{13}C-NMR** (50 MHz, $CDCl_3$): δ 141.2, 136.9, 128.9, 127.3, 64.8, 46.1.

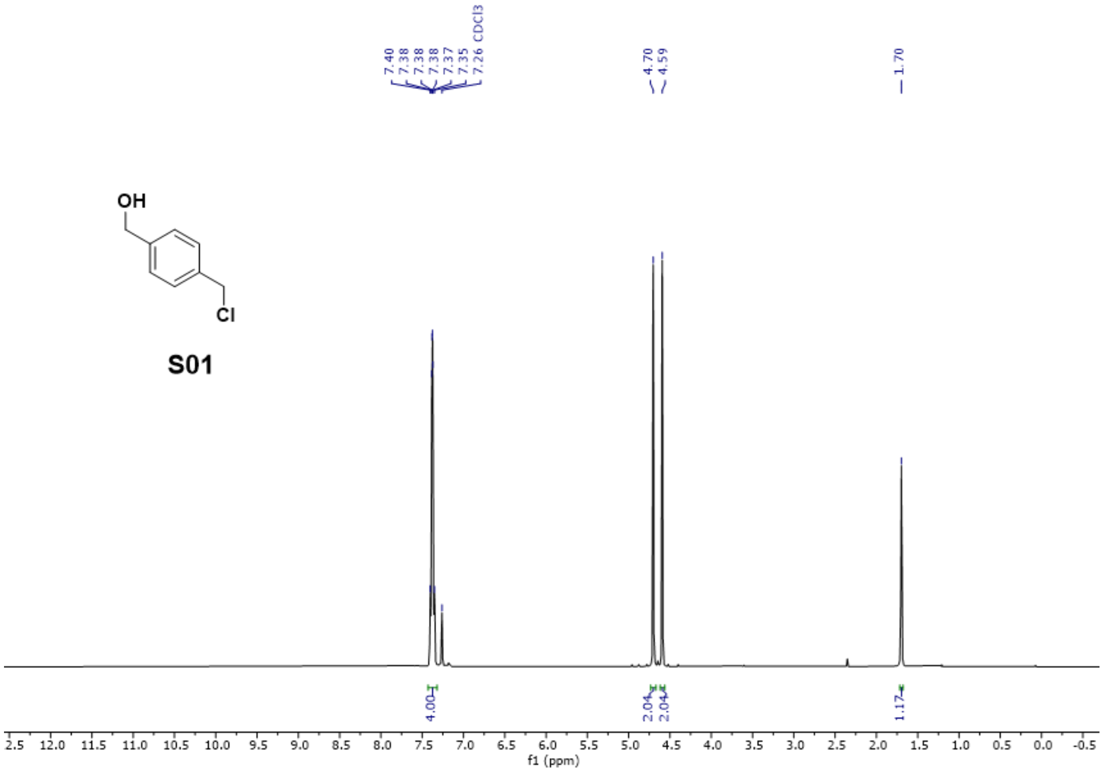

Figure S1. ^{1}H-NMR (400 MHz, $CDCl_3$) spectrum of compound **S01**.

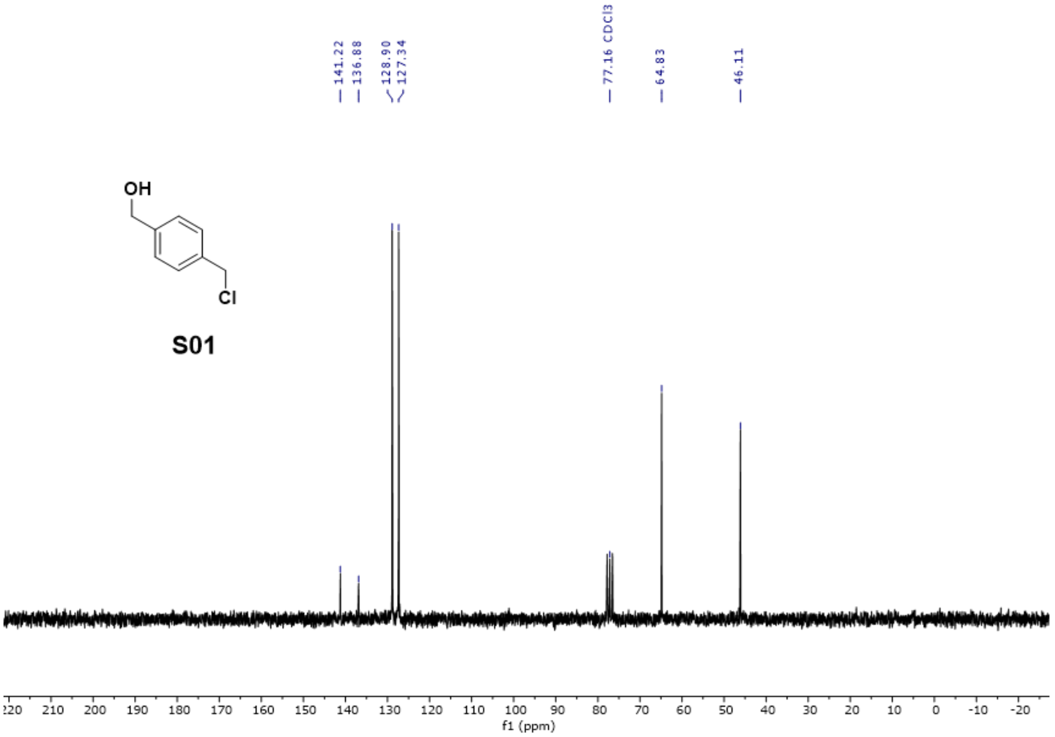

Figure S2. ^{13}C-NMR (50 MHz, $CDCl_3$) spectrum of compound **S01**.

1.2.2 1-(chloromethyl)-4-((prop-2-yn-1-yloxy)methyl)benzene (S02)

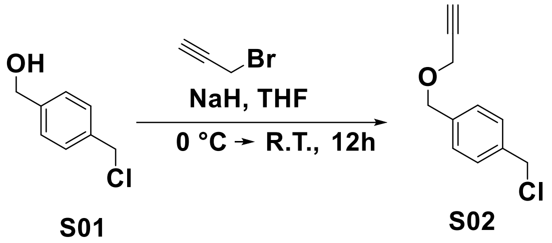

A flame dried round bottom flask was charged with **S01** (600 mg, 3.83 mmol, 1 eq.) and anhydrous THF (20 ml). NaH (184 mg, 4.6 mmol, 60% in mineral oil, 1.2 eq.) was added in portions at 0 °C and the resulting mixture was stirred for 30 min. Then propargyl bromide (855 mg, 2 mmol, 80% in toluene, 1.5 eq.) was added dropwise. The reaction was warmed to room temperature and stirred for 18 h. The reaction was quenched with water and extracted with Et_2O (50 ml x 3). The combined organic layers were dried over Na_2SO_4, filtered, and concentrated under reduced pressure. The residue was purified by column chromatography (PE/EtOAc: 2/8) to afford 1-(chloromethyl)-4-((prop-2-yn-1-yloxy)methyl)benzene as a yellowish oil (537 mg, 72%).

^{1}H-NMR (200 MHz, $CDCl_3$): δ 7.37 (s, 4H), 4.61 (s, 2H), 4.59 (s, 2H), 4.18 (d, *J* = 2.4 Hz, 2H), 2.51 – 2.44 (m, 1H). **^{13}C-NMR** (50 MHz, $CDCl_3$): δ 137.6, 137.0, 128.6, 128.2, 79.5, 74.8, 70.9, 57.1, 45.9. **HRMS (ESI-TOF):** m/z $[M + Na]^+$ Calcd. for $C_{11}H_{11}ClONa$ 217.0391, Found 217.0373.

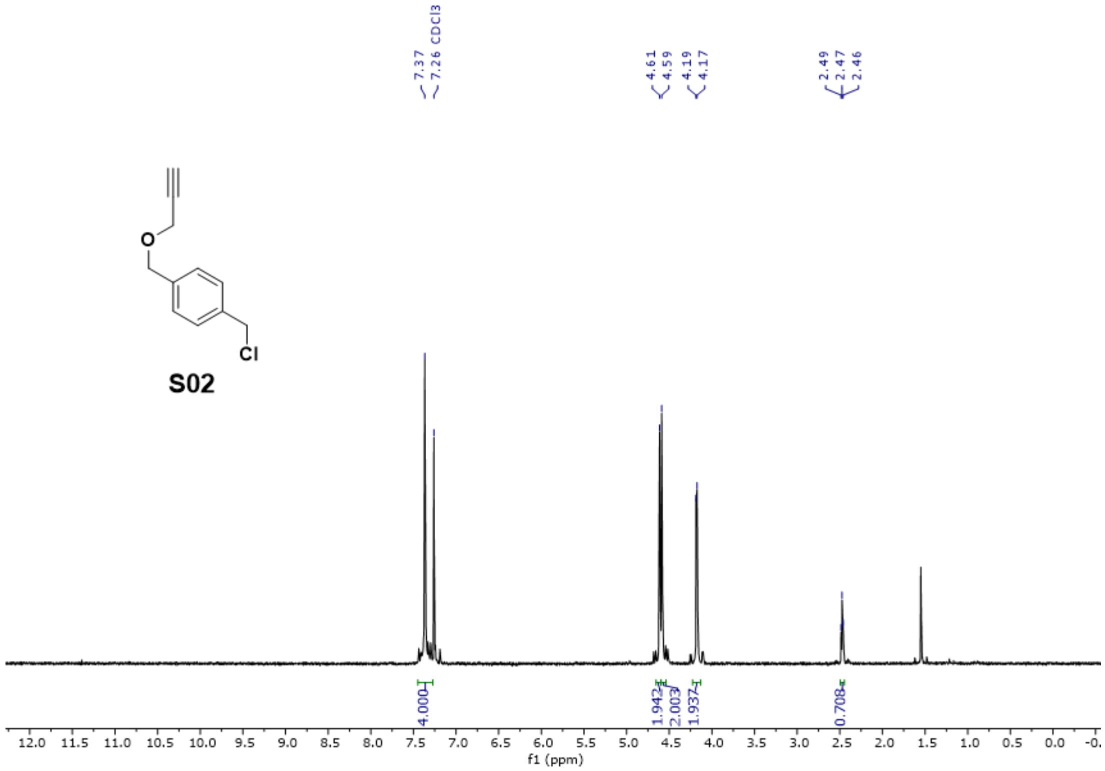

Figure S3. ^{1}H-NMR (200 MHz, $CDCl_3$) spectrum of compound **S02**.

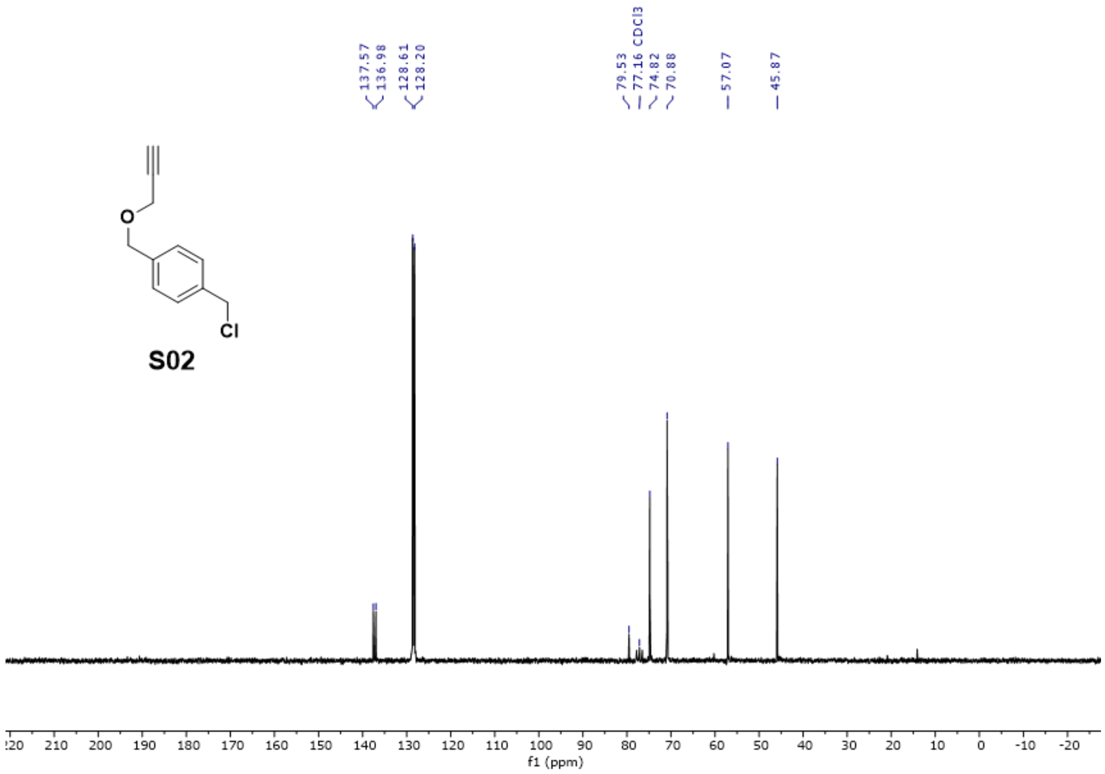

Figure S4. ^{13}C-NMR (50 MHz, $CDCl_3$) spectrum of compound **S02**.

1.2.3 1-mesitylimidazole (S03)

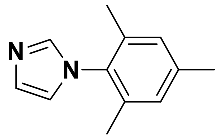

S03

2,4,6-Trimethylaniline (2.00 g, 2.08 mL, 14.8 mmol, 1 eq.) and glyoxal (2.15 g, 1.7 mL, 14.8 mmol, 40% in water, 1 eq.) were added into a round bottom flask and stirred in methanol (10 mL) at 25 °C for 24 h. Followingly, NH_4Cl (1.6 g, 29.6 mmol, 2 eq.), formaldehyde (2.4 g, 2.2 mL, 29.6 mmol, 37% in water) and methanol (60 mL) were added and refluxed for 1 h. After this time, H_3PO_4 (2.1 mL, 85%) was added to the reaction mixture and was refluxed for another 24 h. Then, the solvent was removed *in vacuo*, and the reaction mixture was poured into crushed ice. 40% solution of KOH was gradually added until pH = 9. The resulting mixture was extracted with Et_2O (3 x 100 mL). The collected organic layers were dried over Na_2SO_4, filtered, and concentrated under reduced pressure. The crude product was purified by column chromatography (PE/EtOAc: 2/8 to 1/1) to give 1-mesitylimidazole as a white solid (1.5 g, 54%). The spectral data are in accordance with the literature.[3]

^{1}H-NMR (400 MHz, $CDCl_3$): δ 7.47 (s, 1H), 7.25 (s, 1H), 6.97 (s, 2H), 6.90 (s, 1H), 2.34 (s, 3H), 1.99 (s, 6H). **^{13}C-NMR** (100 MHz, $CDCl_3$): δ 138.9, 137.5, 135.5, 133.5, 129.6, 129.1, 120.1, 21.1, 17.4.

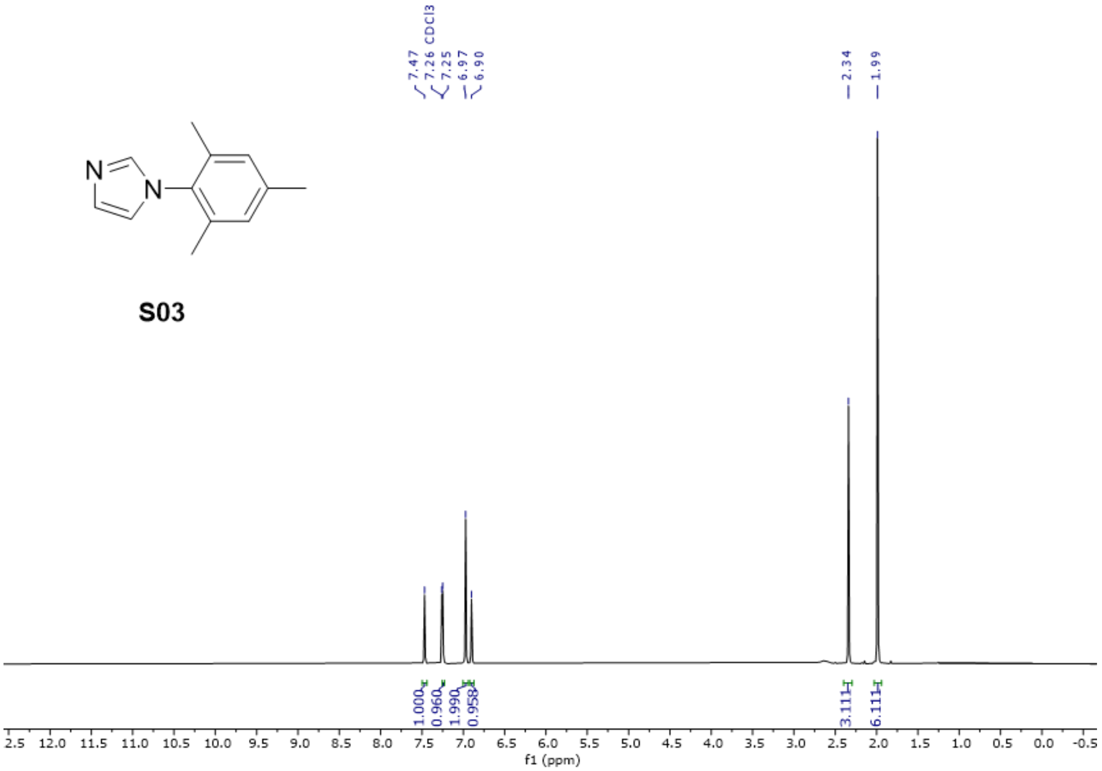

Figure S5. ^{1}H-NMR (400 MHz, $CDCl_3$) spectrum of compound **S03**.

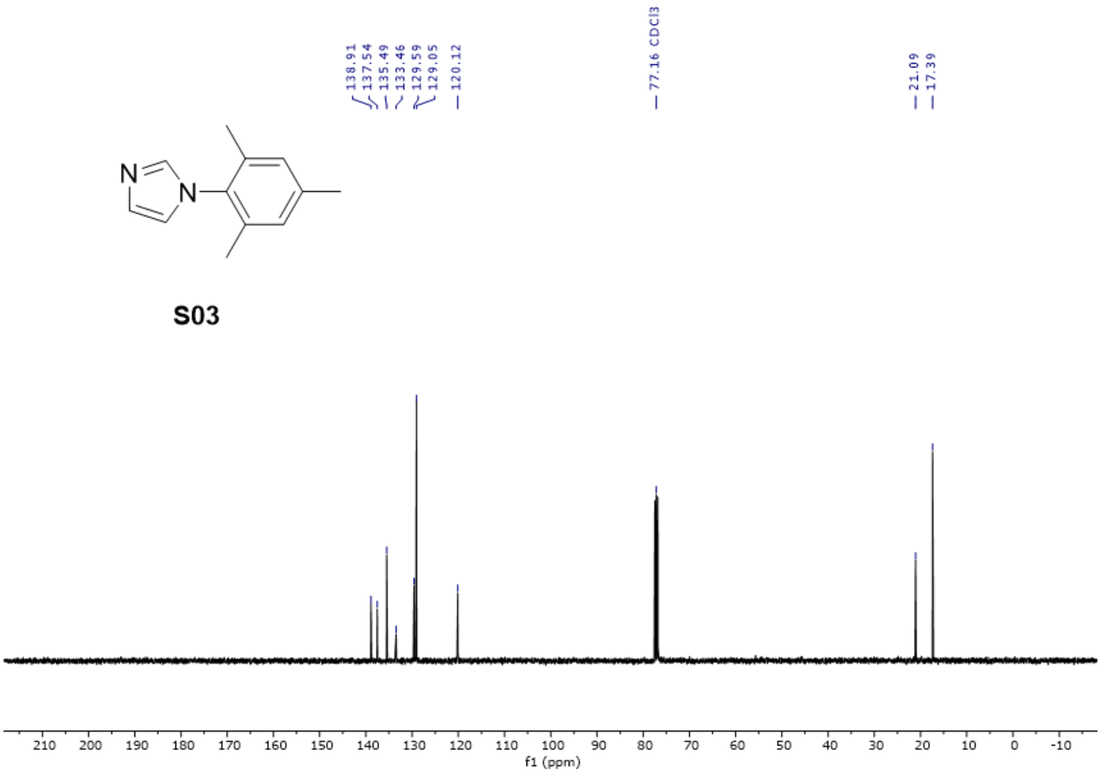

Figure S6. ^{13}C-NMR (100 MHz, $CDCl_3$) spectrum of compound **S03**.

1.2.4 1-mesityl-3-(4-((prop-2-yn-1-yloxy)methyl)benzyl)-1*H*-imidazol-3-ium chloride (4)

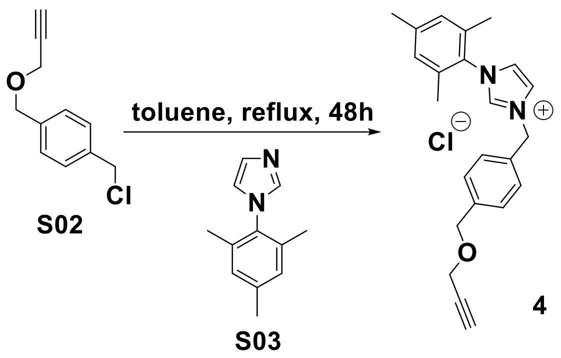

In a Schlenk tube, **S03** (400 mg, 2.15 mmol), **S02** (500 mg, 2.58 mmol), and toluene (10 mL) were added and heated at 120°C for 48 h. Initially, the product formed was a brown, insoluble oil. Then, the reaction was cooled to room temperature, the insoluble oil solidified, and the toluene was decanted. The brown precipitate was dissolved in CH_2Cl_2 and concentrated under reduced pressure to afford the product as a light brown solid (565 mg, 69%).

^{1}H NMR (400 MHz, $CDCl_3$): δ 11.07 (d, *J* = 10.1 Hz, 1H), 7.60 – 7.54 (m, 2H), 7.50 – 7.35 (m, 3H), 7.06 (s, 1H), 7.00 (s, 2H), 6.00 (s, 2H), 4.61 (s, 2H), 4.23 – 4.18 (m, 2H), 2.51 – 2.46 (m, 1H), 2.34 (s, 3H), 2.06 (s, 6H). **^{13}C NMR** (100 MHz, $CDCl_3$): δ 141.1, 138.6, 138.0, 134.1, 133.5, 130.7, 129.7, 129.2, 128.8, 123.3, 123.0, 79.3, 74.9, 70.9, 57.4, 52.9, 21.0, 17.5. **HRMS (ESI-TOF):** m/z [M - Cl]$^+$ Calcd. for $C_{23}H_{25}N_2O^+$ 345.1961, Found 345.1982.

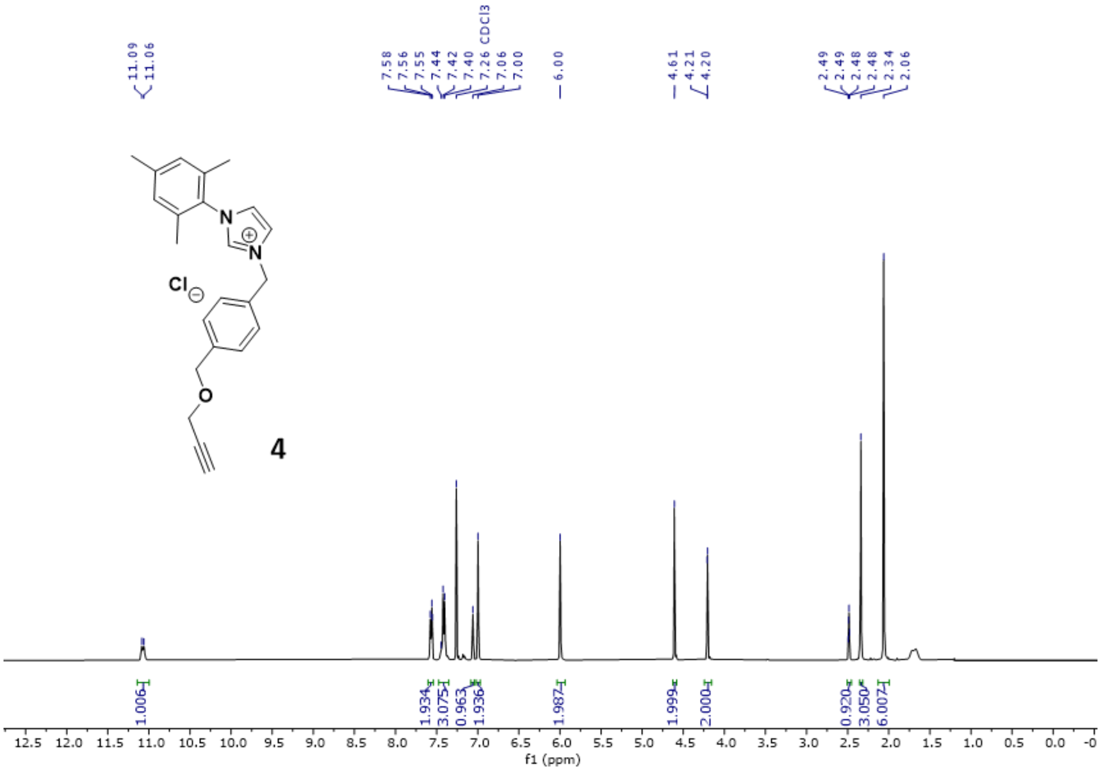

Figure S7. ^{1}H-NMR (400 MHz, $CDCl_3$) spectrum of compound **4**.

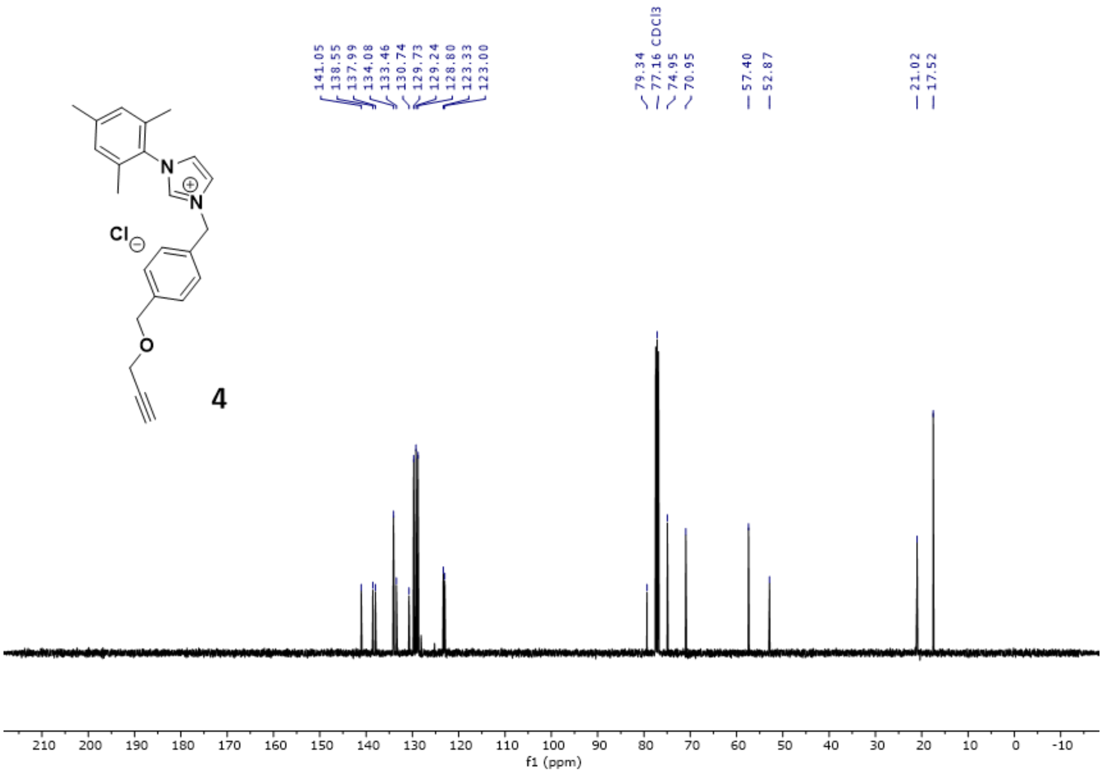

Figure S8. ^{13}C-NMR (100 MHz, $CDCl_3$) spectrum of compound **4**.

1.2 Preparation of MWCNTs (1)

Removal of metal residues from MWCNTs via acid treatment (1)

In a round bottom flask charged with 500 mg MWCNTs (NanoAmor, CNTs > 95%), 250 mL of concentrated HCl (36% $^{v}/_{v}$) were added. The mixture was briefly sonicated for 1 h and then stirred under reflux for 4h. Afterwards, it was poured into a flask containing 500 mL of ice-cold distilled water and filtered through PTFE membrane (47 mm diameter, 0.2 μm pore size), followed by extensive washing with distilled water and finally with EtOH. The solid residue was dried at 90 ˚C for 10 h. 495 mg of cleaned MWNTs (with lesser metal traces) were obtained as a black amorphous powder.

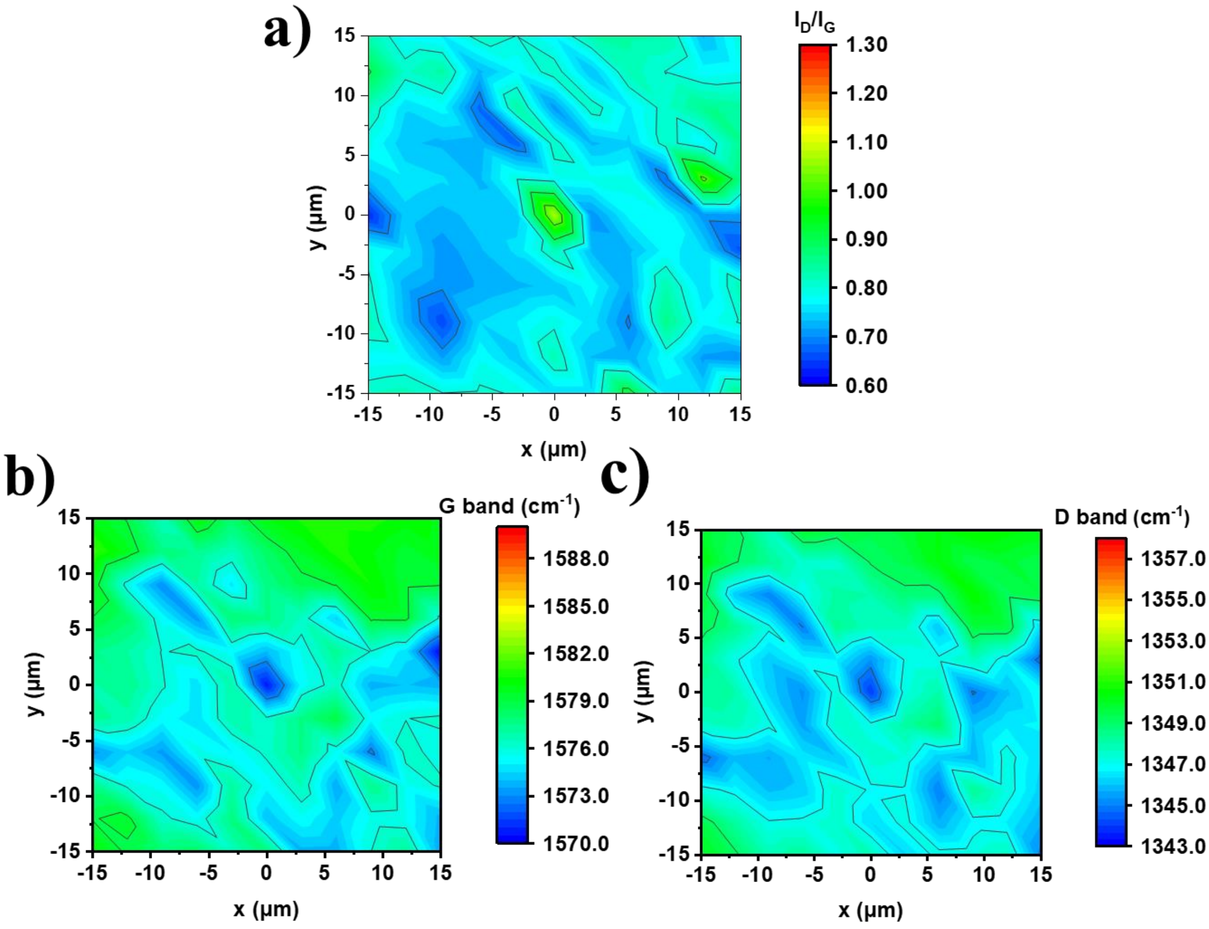

Figure S9. Raman 30 μm x 30 μm spectral maps of **1**. a) Color scale: D/G intensity ratio (average: 0.80) and b, c) Color scale: G (left) and D (right) band position, (average G band position: 1577 cm^{-1} and average D band position 1348 cm^{-1}).

1.3 Synthesis of 4-azido aniline (2)

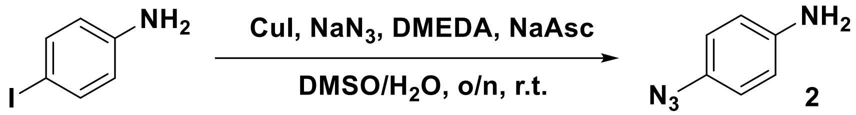

Scheme S2. Synthesis of 4-azido aniline (**2**).

A round bottom flask was charged with a degassed DMSO:H_2O (50 mL, 5:1) mixture, 4-iodo aniline (1.75 g, 8 mmol, 1 eq.), NaN_3 (1.04 g, 16 mmol, 2 eq.), sodium ascorbate/NaAsc (80 mg, 0.4 mmol, 0.05 eq.), CuI (152 mg, 0.8 mmol, 0.1 eq.), and *N, N'*-dimethylethylene diamine (106 mg, 1.2 mmol, 0.15 eq.). The dark green mixture was stirred under argon for 18 h at room temperature. Then the reaction mixture was poured in a separation funnel containing EtOAc (200 mL) and brine (200 mL). The phases were separated, and the aqueous phase was extracted with EtOAc (3 x 100 mL). The combined organic phases were dried over Na_2SO_4. After filtration, the solvent was concentrated under reduced pressure. The crude product was passed through a short silica gel column with CH_2Cl_2 to afford 4-azido aniline as orange crystals (966 mg, 90%). The spectral data are in accordance with the literature.[1]

^{1}H-NMR (400 MHz, $CDCl_3$): 6.84 (d, *J* = 8.5 Hz, 1H), 6.67 (d, *J* = 8.6 Hz, 1H), 3.64 (br, 1H).
^{13}C-NMR (100 MHz, $CDCl_3$): δ 143.9, 130.3, 120.1, 116.4.

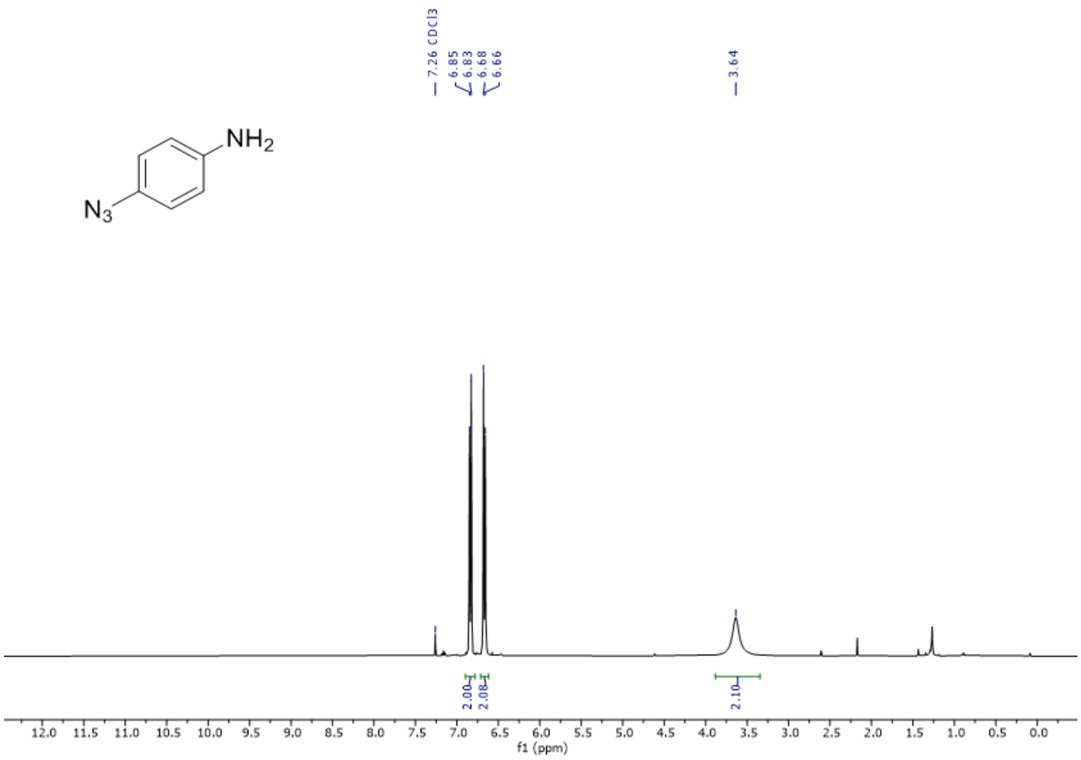

Figure S10. ^{1}H-NMR (400 MHz, $CDCl_3$) spectrum of compound **2**.

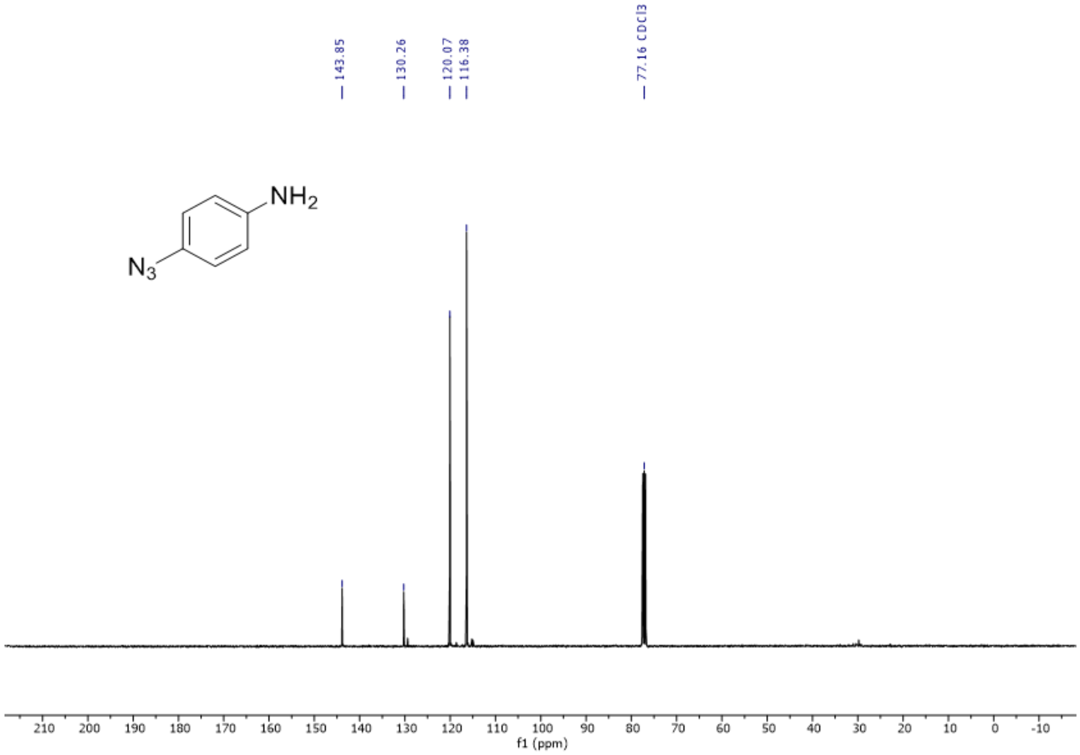

Figure S11. ^{13}C-NMR (100 MHz, $CDCl_3$) spectrum of compound **2**.

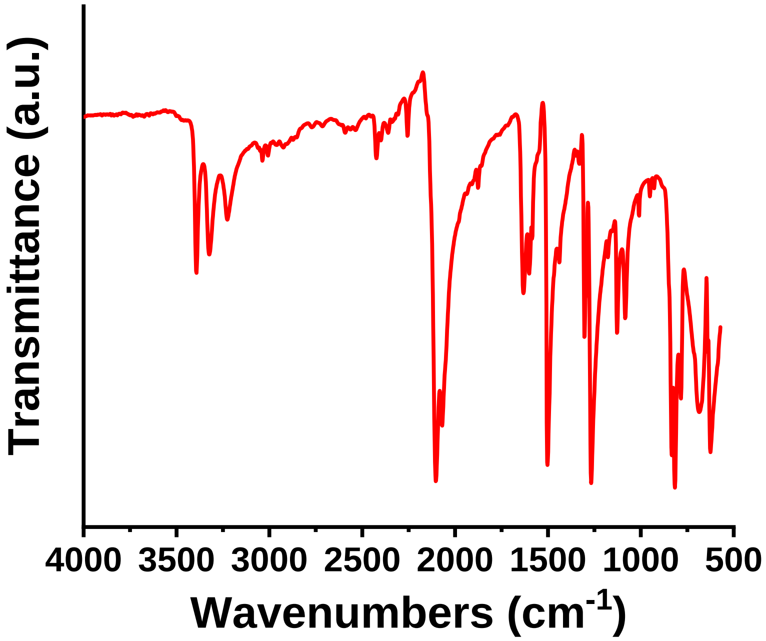

Figure S12. FT-IR spectrum of compound **2**.

1.4 Preparation and characterization of MWCNTs-functionalized nanomaterials (3, 5 and 6).

1.4.1 Azido-functionalized MWCNTs (MWCNTs-N_3, 3)

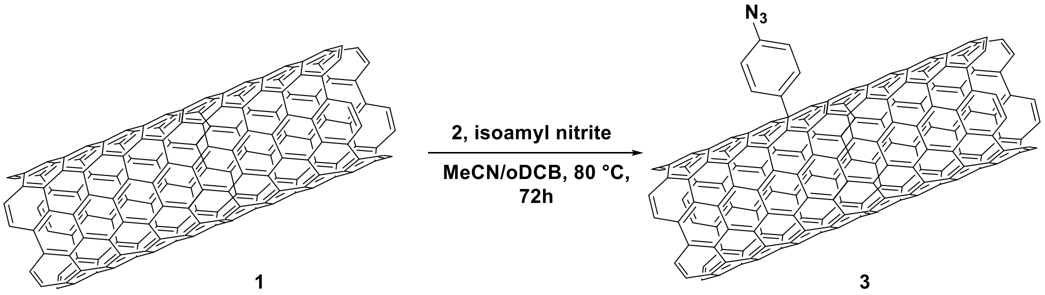

Scheme S3. Preparation of **MWCNTs-N_3** (**3**).

A dry two-necked round bottom flask equipped with a condenser was charged with MWCNTs **1** (50 mg) and **2** (1.14 g, 8.5 mmol, 2 eq.). After the system was deaerated under high vacuum, 50 mL of 1,2-dichlorobenzene and 25 mL of acetonitrile were added. Afterwards, the mixture was extensively degassed with alternating vacuum-N_2 cycles, isoamyl nitrite (2.9 g, 3.4 mL, 25 mmol, 6 eq.) was quickly added and then additional vacuum-N_2 cycles were performed. The mixture was briefly sonicated, and it was left stirring at 80 ˚C under N_2 for 72 h. The reaction mixture was diluted with DMF, briefly sonicated, filtered through PTFE membrane (47 mm diameter, 0.2 μm pore size), and extensively washed with DMF, DCM and EtOH. The product was dispersed in EtOH, dried under air stream and then at 90 ˚C for 12h. 50 mg of **3** were collected as amorphous black powder.

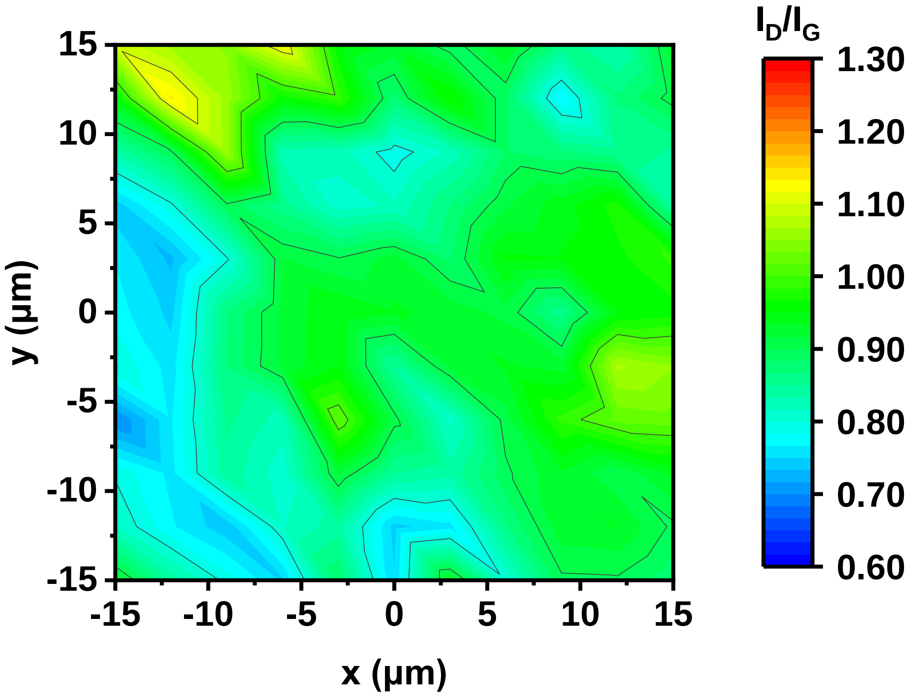

Figure S13. Raman 30 μm x 30 μm spectral map of **3**. Color scale: D/G intensity ratio (average: 0.91).

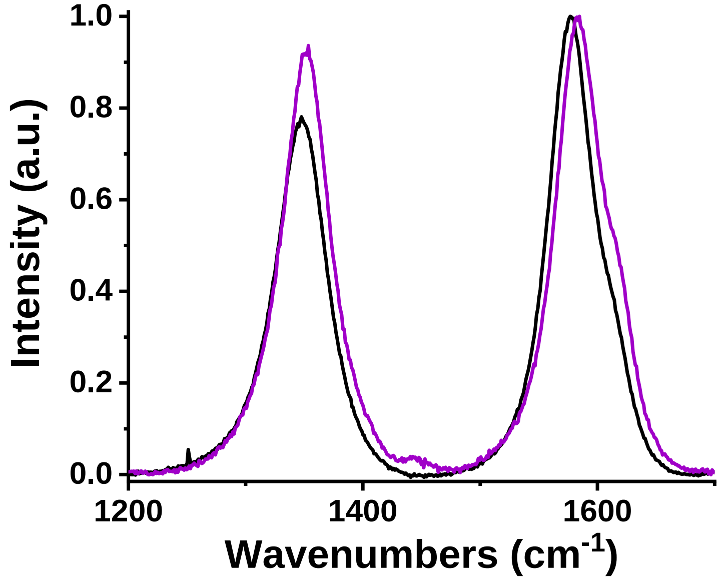

Figure S14. Comparative Raman spectra focusing on D and G band region of **1** (black) and **3** (purple).

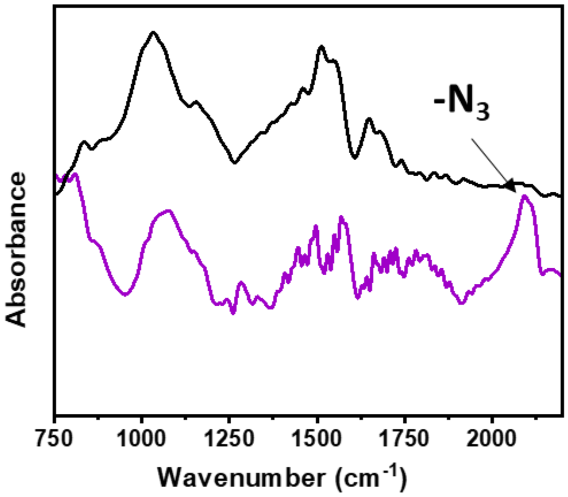

Figure S15. Comparative ATR-FTIR spectra of **1** (black) and **3** (purple).

1.4.2 Preparation of CuAAC "click-derivative" nanomaterial (MWCNTs-NHC, 5)

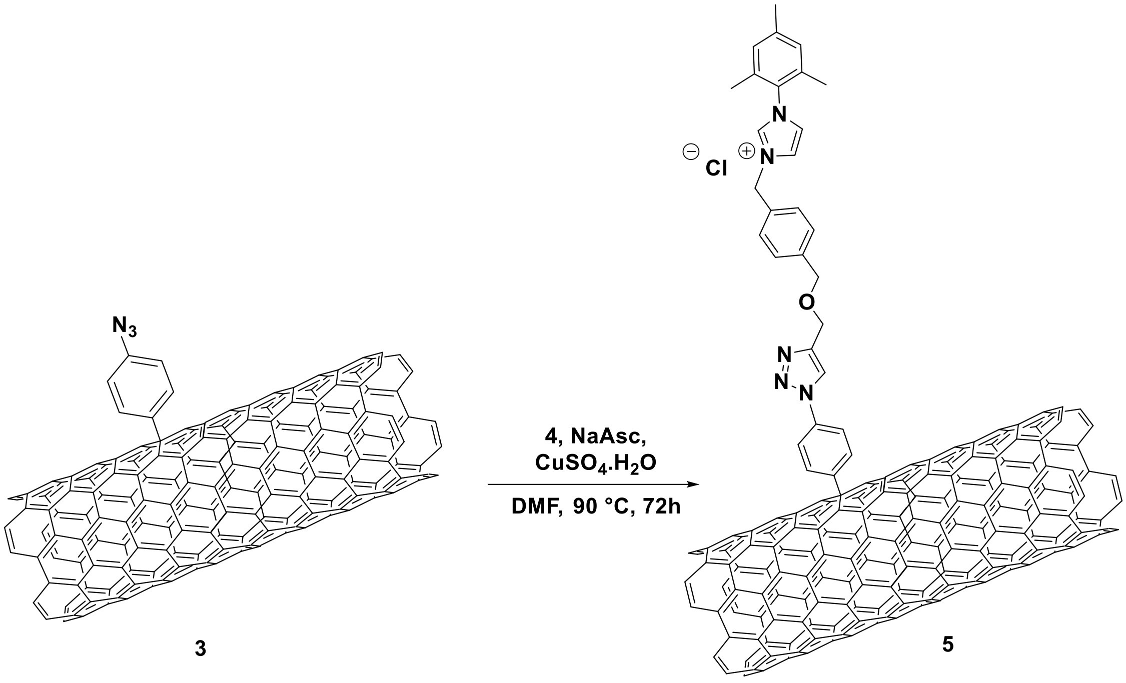

Scheme S4. Preparation of **MWCNTs-NHC** (**5**).

To a degassed 24 mL of a dispersion of **3** (1 mg/mL) in DMF, **4** (240 mg, 0.63 mmol, 52 eq.) was added as a solution in 4 mL DMF, along with NaAsc (142 mg, 0.73 mmol, 60 eq.) and $CuSO_4 \cdot H_2O$ (77 mg, 0.3 mmol, 25 eq.) under N_2 flow and the mixture was left stirring at 90 ˚C for 72 h. The reaction mixture was filtered through PTFE membrane (47 mm diameter, 0.2 μm pore size) and washed extensively with DMF, DCM and EtOH. The solid residue was redispersed in EtOH, diluted with 1 L of distilled water containing 1.4% $^{v}/_{v}$ of 22% aqueous NH_4OH solution and left to settle overnight. The mixture was filtered again and washed with distilled water and EtOH. The solid residue was dispersed in EtOH, dried under air stream, and then dried in an oven at 90 ˚C for 10h. 23 mg of **5** were collected as amorphous black solid.

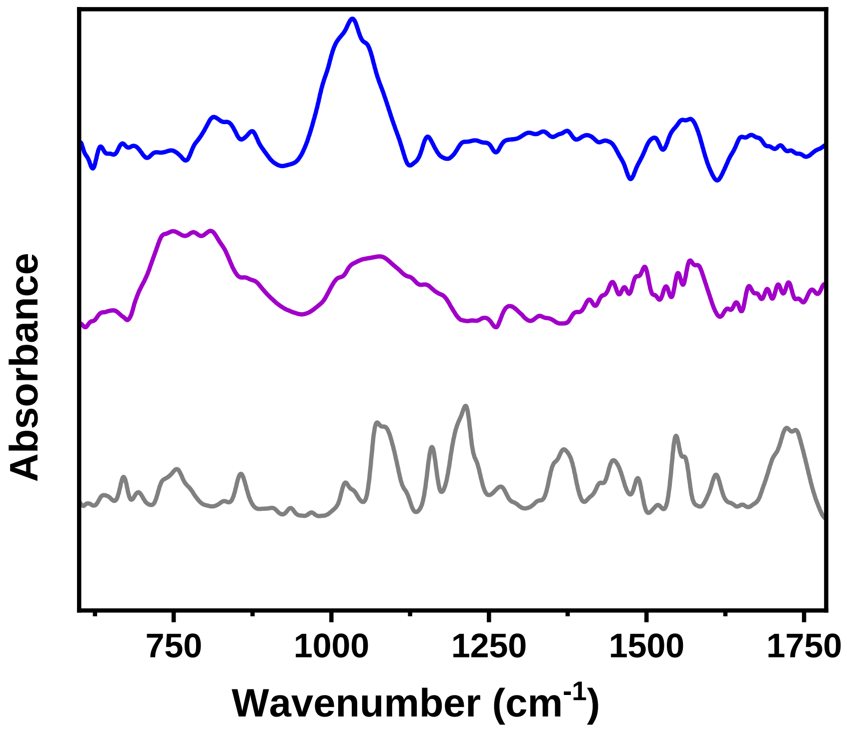

Figure S16. Comparative ATR-FTIR spectra of MWCNTs-N_3 **3** (purple), ligand **4** (grey), and click-MWCNTs-NHC **5** (blue).

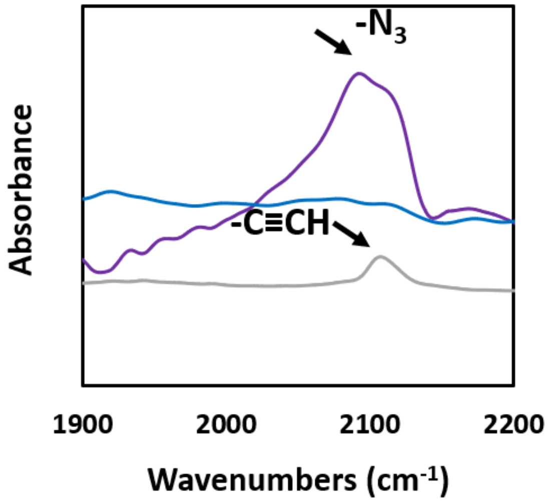

Figure S17. Comparative ATR-FTIR spectra, focusing on azide/alkyne region, of MWCNTs-N_3 **3** (purple), ligand **4** (grey), and click-MWCNTs-NHC **5** (blue).

Table S1. Observed ATR-FTIR bands of **3-5** and their assignment.[S6, S7]

Assignements	3	4	5
**$-N_3$**	2096 cm^{-1}	**-**	**-**
**-C≡CH**	**-**	2110 cm^{-1}	**-**
**$-C=N^+$**	-	1720 cm^{-1}	1720 cm^{-1}
**Arom. C=C (MWCNTs)**	1565 cm^{-1}	-	1565 cm^{-1}
**Arom. C=C (Ligand) & Imid. C=N ring**	-	1549 cm^{-1}, 1485 cm^{-1} and 1445 cm^{-1}	1549 cm^{-1}, 1485 cm^{-1} and 1445 cm^{-1}
**N=N (triazole)**	-	-	1406 cm^{-1}
**Imid. C=N/C-N ring stretch.**	-	1374 cm^{-1}	-
**C-N stretch.**	-	1215 cm^{-1}	-
**(N)-CH_2- stretching & C-O-C**	-	1147 cm^{-1}, 1066 cm^{-1}	1147 cm^{-1}, 1066 cm^{-1}
**CC, CH & arom. C-C ring stretch.**	-	1023 cm^{-1}	1023 cm^{-1}
**N(CH)N bend. & ring HCCH bend.**	-	856 cm^{-1}	-
**Ring CC bend**	813 cm^{-1}	-	813 cm^{-1}
**ring HCCH bend. & CC, C=C-H**	-	754 cm^{-1}	754 cm^{-1}

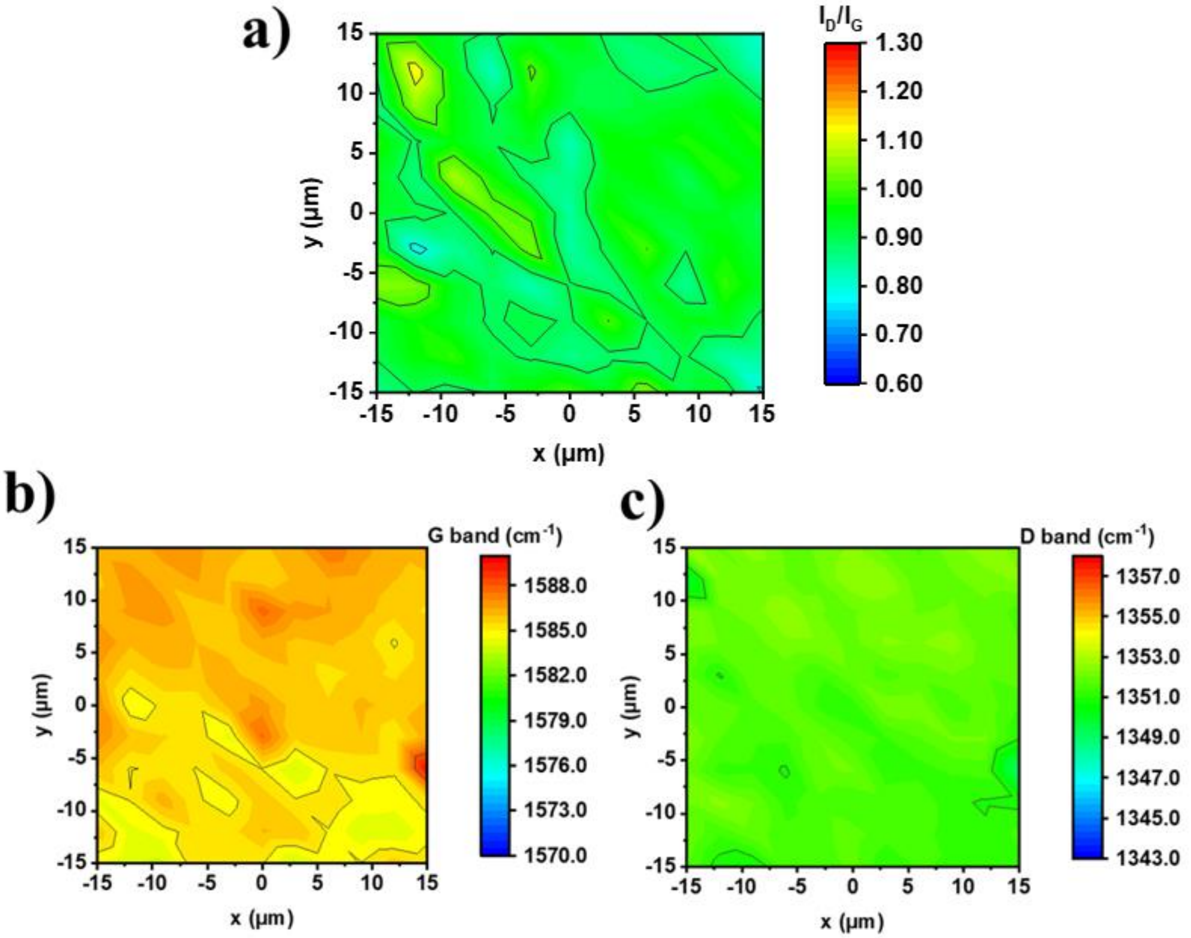

Figure S18. Raman 30 µm x 30 µm spectral maps of **5** a) Color scale: D/G intensity ratio (average: 0.91) and b) Color scale: G (left) and D (right) band position (average G band position: 1586 cm^{-1} and average D band position: 1352 cm^{-1}).

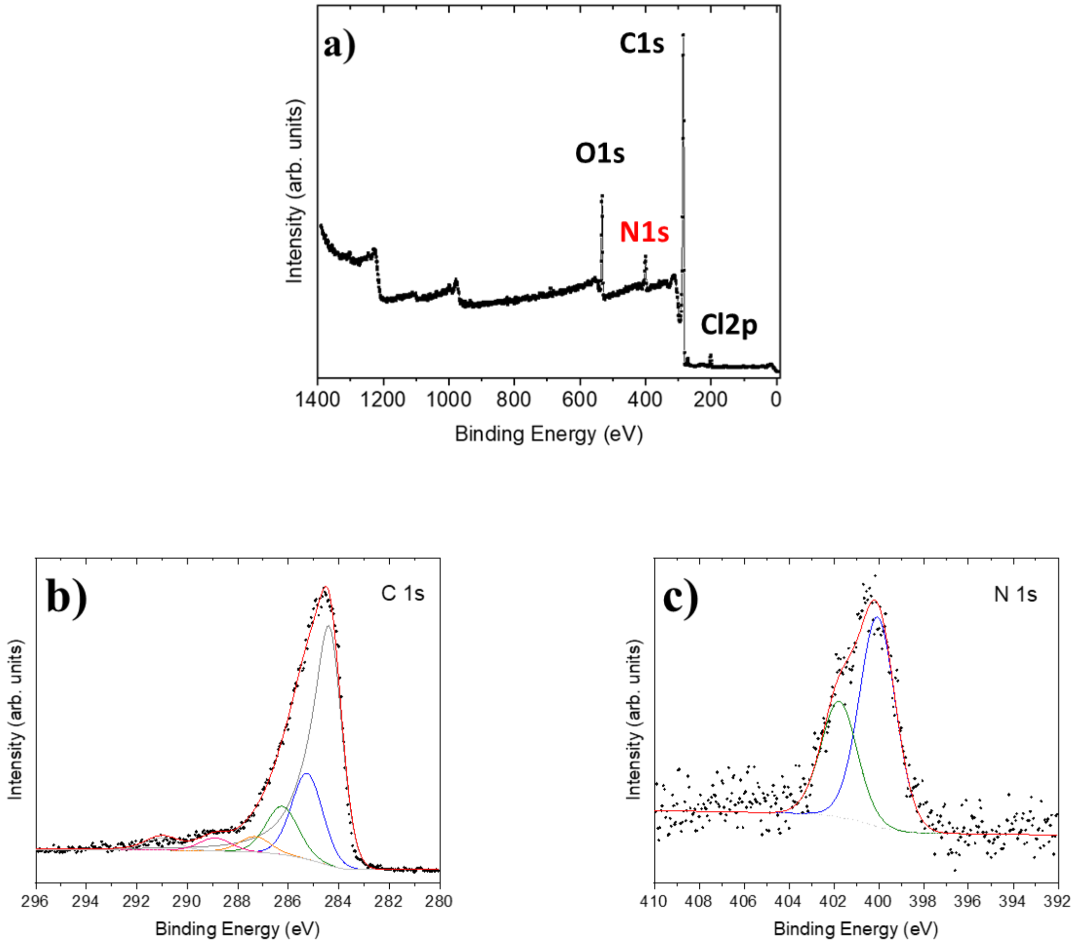

**Nanomaterial 5**	**Normalized Areas**	**Atomic Percentage C : N : Cu (%)**
**C 1s**	1175.1	94.2
**N 1s**	72.3	5.8
**Cu 2p3/2**	0	0

Figure S19. XPS a) survey scan and b, c) narrow scans of C1s and N1s, respectively, of nanomaterial **5**. The table below shows normalized areas and atomic percentages, calculated with high-resolution XPS narrow scans.

1.4.3 Preparation of Cu (I) carbene-functionalized MWCNTs (MWCNTs-CuNHC, 6)

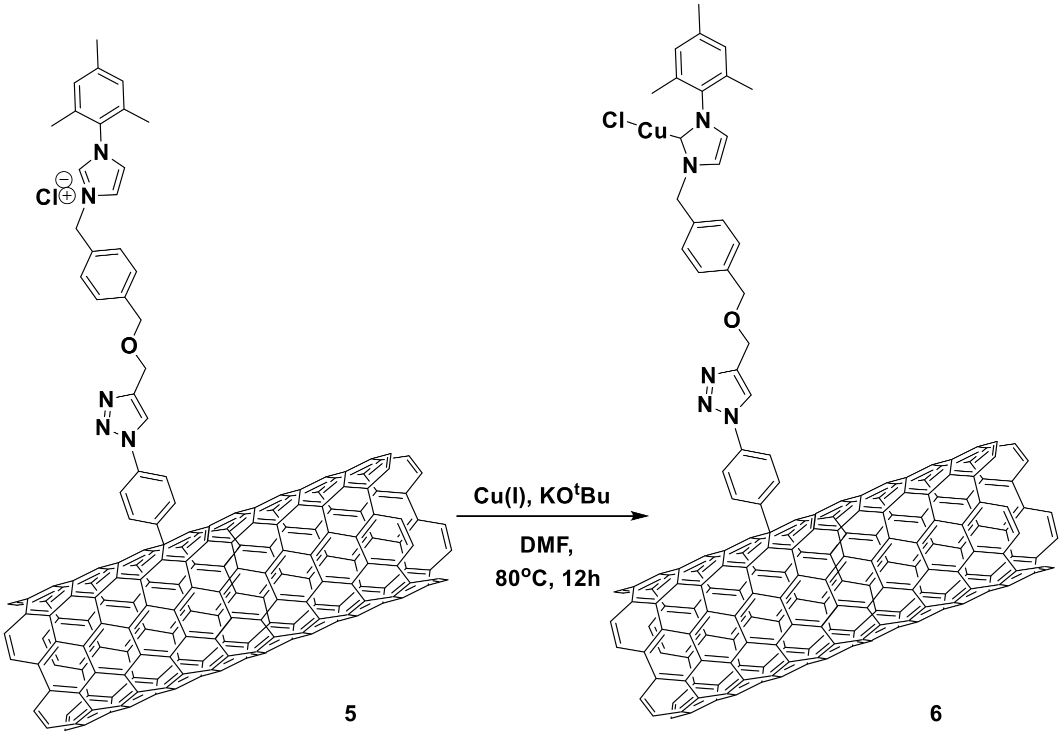

5 6

Scheme S5. Preparation of **MWCNTs-CuNHC** (**6**).

To a degassed dispersion of **5** (0.67 mg/mL) in 25.4 mL DMF, CuCl (26 mg, 0.26 mmol, 35 eq.) and KO^{t}Bu (29 mg, 0.26 mmol, 35 eq.) were added under N_2 atmosphere. The mixture was left stirring at 25 °C for 48 h. The dispersion was filtered through PTFE membrane and washed three times with DMF and EtOH after sonicating it each time for 5 min. The solid residue was redispersed in EtOH, diluted with 100 mL of distilled water containing 1.4% $^v/_v$ of 22% aqueous NH_4OH solution and left to settle overnight. The mixture was filtered again and washed with distilled water and EtOH. The solid residue was redispersed in EtOH, dried under N_2 stream and was subsequently dried in an oven at 80 ˚C for 10 h. 16 mg of amorphous black solid were collected.

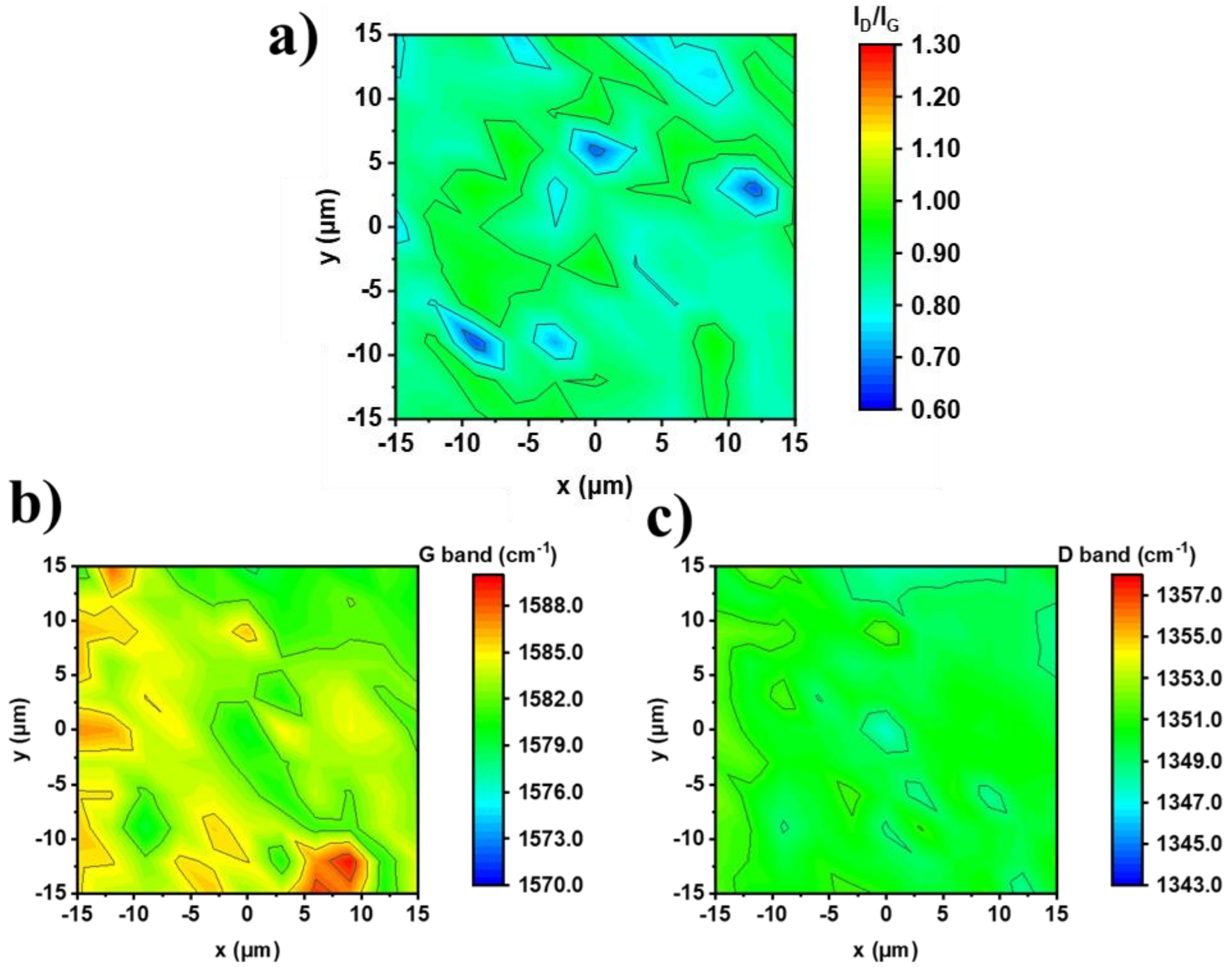

Figure S20. Raman 30 μm x 30 μm spectral maps of **6** a) Color scale: D/G intensity ratio (average: 0.9) and b) Color scale: G (left) and D (right) band position (average G position: 1583 cm^{-1} and average D position: 1350 cm^{-1}).

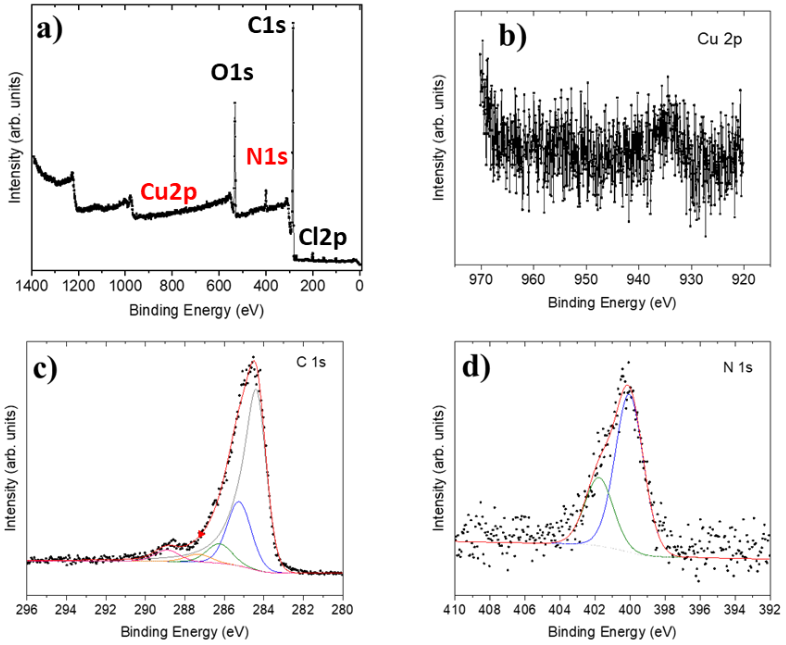

**Nanomaterial 6**	**Normalized Areas**	**Atomic Percentage C : N : Cu (%)**
**C 1s**	779	94.1
**N 1s**	45	5.4
**Cu 2p3/2**	4	0.5

Figure S21. XPS a) survey scan, b) Cu 2p scan and c, d) narrow scans of C1s and N1s, respectively, of nanomaterial **6**. The table below shows normalized areas and atomic percentages calculated with high-resolution narrow scans.

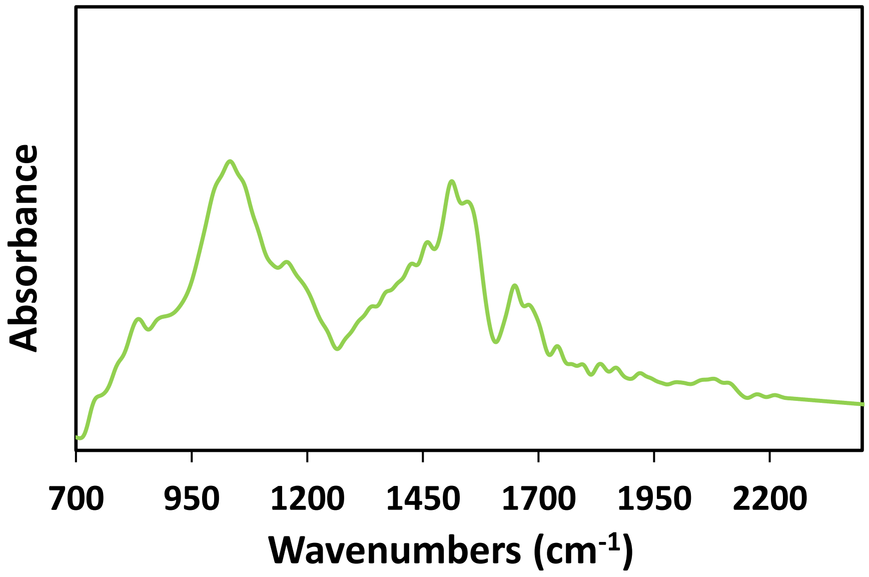

Figure S22. ATR-FTIR spectrum of **6**.

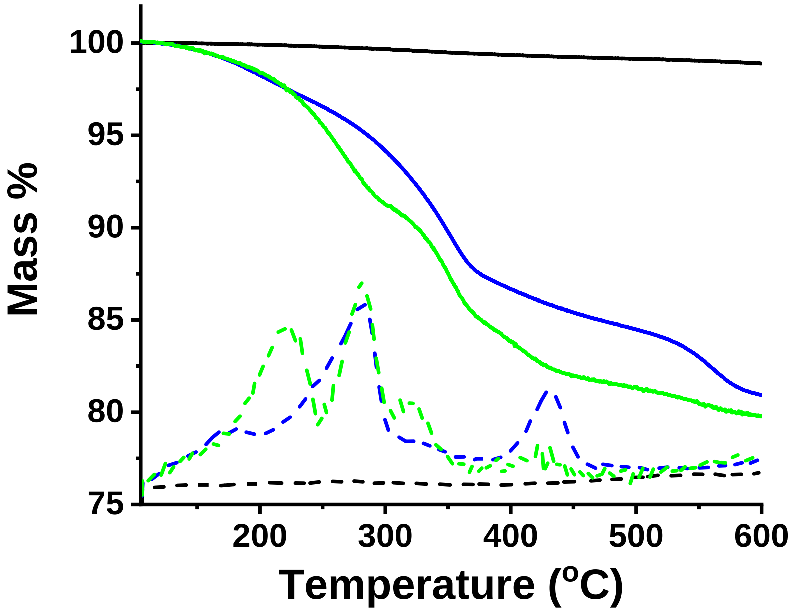

Figure S23. Comparative TGA thermographs (solid lines) and derivatives of % mass to temperature (dashed lines) of **1** (black), **5** (blue) and **6** (green).

1.5 Preparation of advanced electrocatalyst 8.

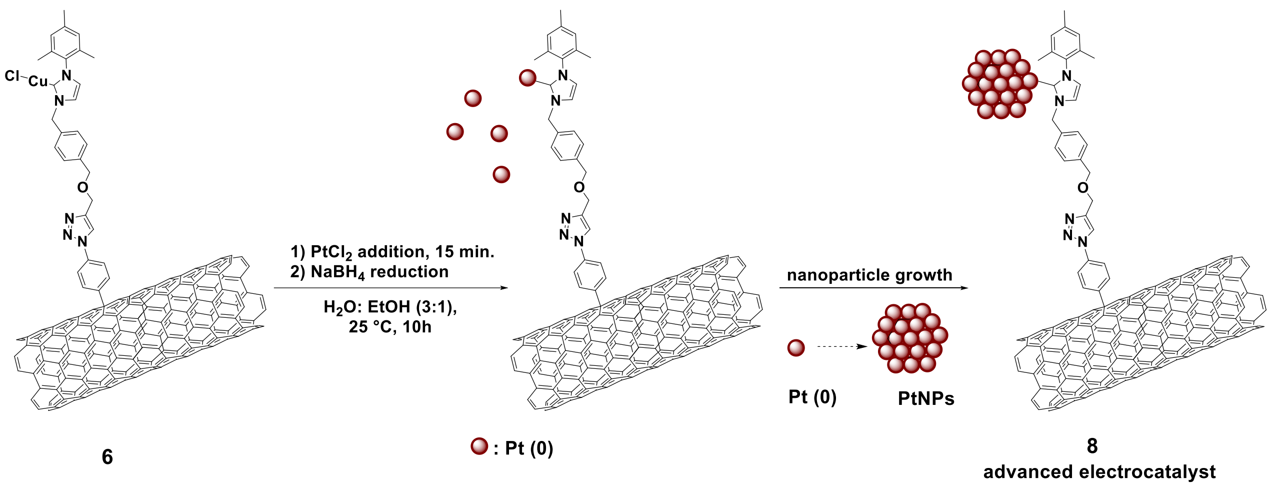

Scheme S6. Preparation of advanced electrocatalyst **8.**

To a degassed dispersion of **6** (6 mg) in 18 mL of a H_2O:EtOH (3:1) mixture, $PtCl_2$ (0.4 mg, 0.002 mmol) was added in one portion and the mixture was briefly sonicated and left to stir vigorously under N_2 for 15 min. $NaBH_4$ (1.5 mg, 0.04 mmol) was then added and the mixture was left to stir vigorously for 10 h under N_2. Impurities were removed via two-fold dilution with H_2O:EtOH (3:1), centrifugation (4000 rpm, 20 min) and three cycles of redispersion and centrifugation of precipitate, twice in H_2O:EtOH (3:1) and once in EtOH. The solid residue was dried under N_2 stream and at 80 ˚C overnight. 6 mg of amorphous black solid were finally collected.

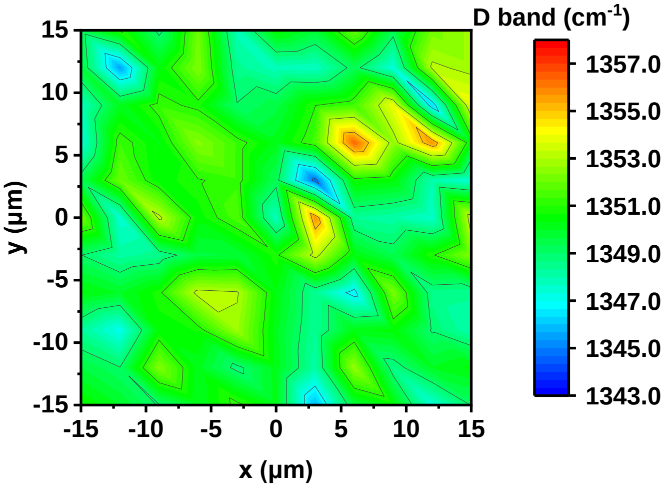

Figure S24. Raman 30 μm x 30 μm spectral map of **8**. Color scale: D band position (average D band position: 1353 cm^{-1}).

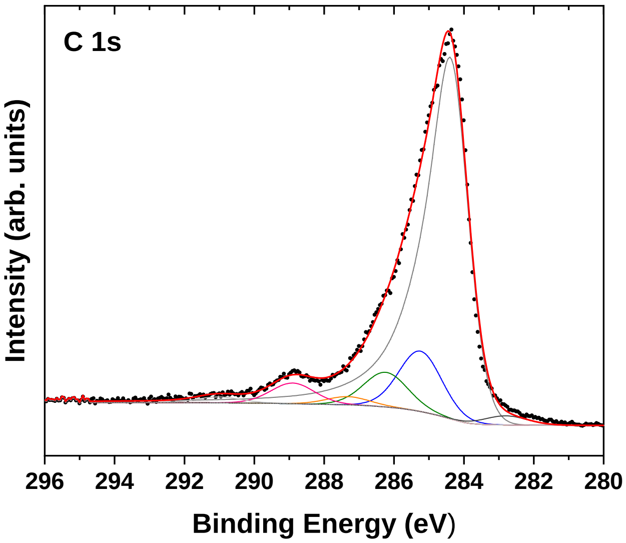

Nanomaterial 8	Normalized Areas	Atomic Percentage C : N : Cu : Pt (%)
**C 1s**	1107	94.76
**N 1s**	58	4.90
**Cu 2p3/2**	0	0.00
**Pt 4f**	4	0.34

Figure S25. XPS narrow scan of C 1s of advanced electrocatalyst **8**. The table below shows normalized areas and atomic percentages calculated with high-resolution narrow scans. Pt/C ratio is 0.4 %.

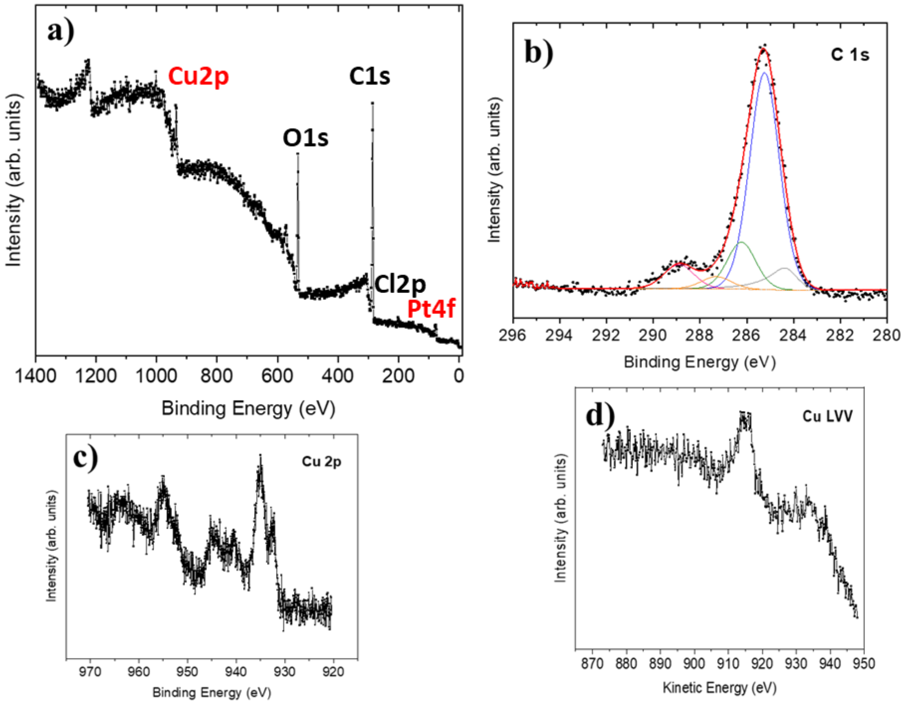

Nanomaterial 8	Atomic Percentage C : N : Cu : Pt (%)
C 1s	94.4
N 1s	0.0
Cu 2p3/2	5.0
Pt 4f	0.6

Figure S26. XPS a) survey scan, b) narrow scan of C 1s, c) Cu 2p scan and d) Cu LVV of by-product collection during preparation of advanced electrocatalyst **8**. The table below shows atomic percentages calculated with high-resolution narrow scans.

Note: The survey scan of the by-products (after solvent evaporation and meticulous washes with distilled water to remove salt residues) detected the elements C, Pt, Cu, and O. Both the lack of nitrogen N1s signals and the minimal comparatively characteristic C1s sp^2 peak intensity, reveal the absence of carbon nanotubes in the sample area. Furthermore, high-resolution spectra of Cu 2p revealed the presence of two components, corresponding to metallic Cu (0) and CuO. This finding supports the idea that unsupported (on the carbon nanoframework) metal nanoparticles formed in the reaction media, including Cu and Pt, were washed out as by-products during the centrifugation process in water.

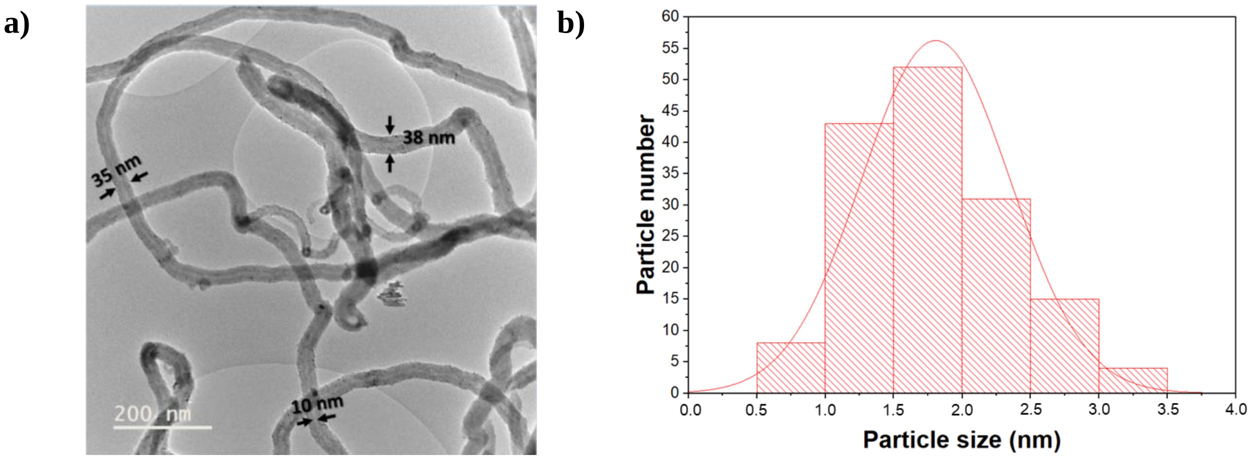

Figure S27. a) Characteristic TEM micrograph of advanced electrocatalyst **8**. Arrows indicate the outer wall diameter (35 nm and 38 nm), or inner wall diameter (10 nm), b) PtNPs' size (nm) histogram of Fig. 4b.

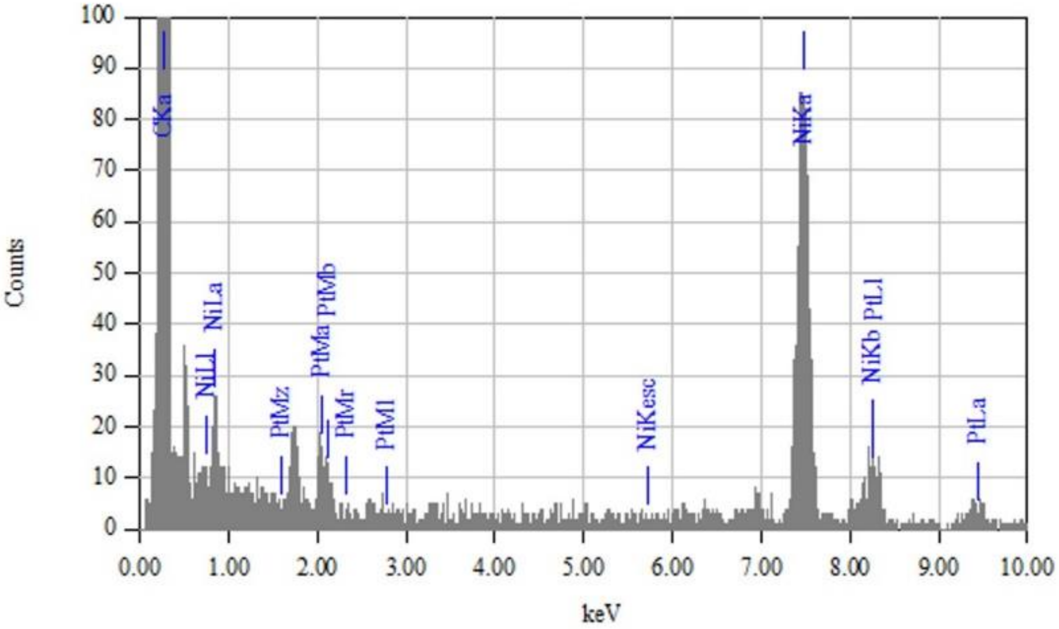

Figure S28. EDS spectrum of advanced electrocatalyst **8** on Ni grid.

1.6 Preparation of control electrocatalyst 7.

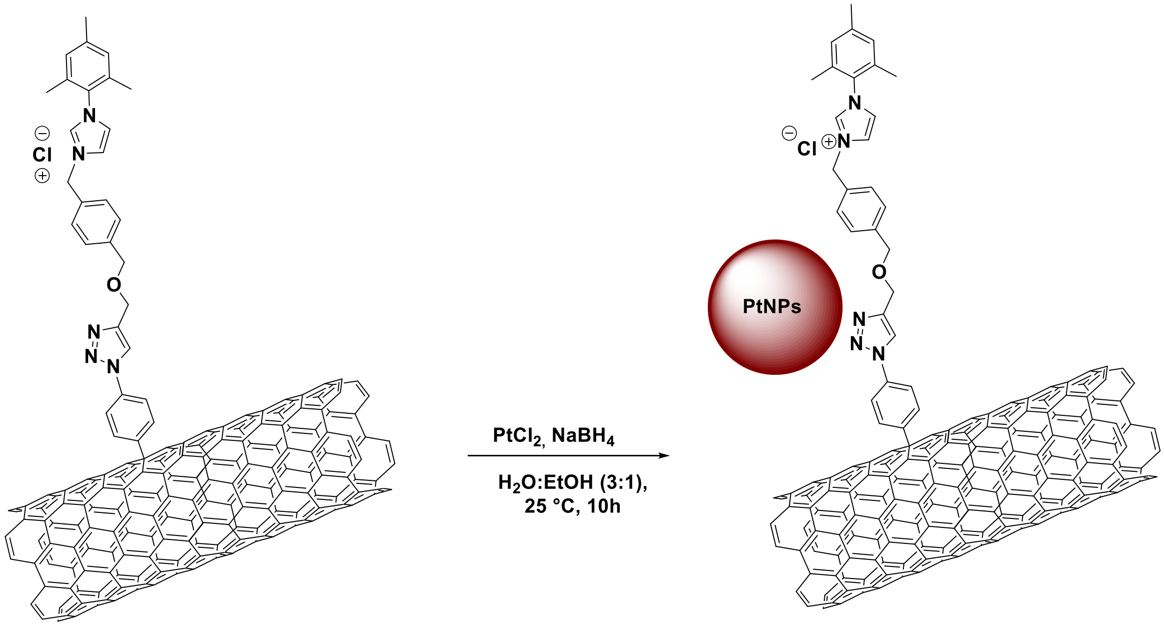

5

7 (control electrocatalyst)

Scheme S7. Preparation of control electrocatalyst **7**.

To a degassed dispersion of **5** (6 mg) in 18 mL of a H_2O:EtOH (3:1) mixture, $PtCl_2$ (0.4 mg, 0.002 mmol) was added in one portion and the mixture was briefly sonicated and left to stir vigorously under N_2 for 15 min. $NaBH_4$ (1.5 mg, 0.04 mmol) was then added and the mixture was left to stir vigorously for 10 h under N_2. Removal of impurities was carried out via two-fold dilution with H_2O:EtOH (3:1), centrifugation (4000 rpm, 20 min) and three cycles of redispersion and centrifugation of precipitate, twice in H_2O:EtOH (3:1) and once in EtOH. The solid residue was dried under N2 stream and at 80 ˚C overnight. 6 mg of amorphous black solid were finally collected.

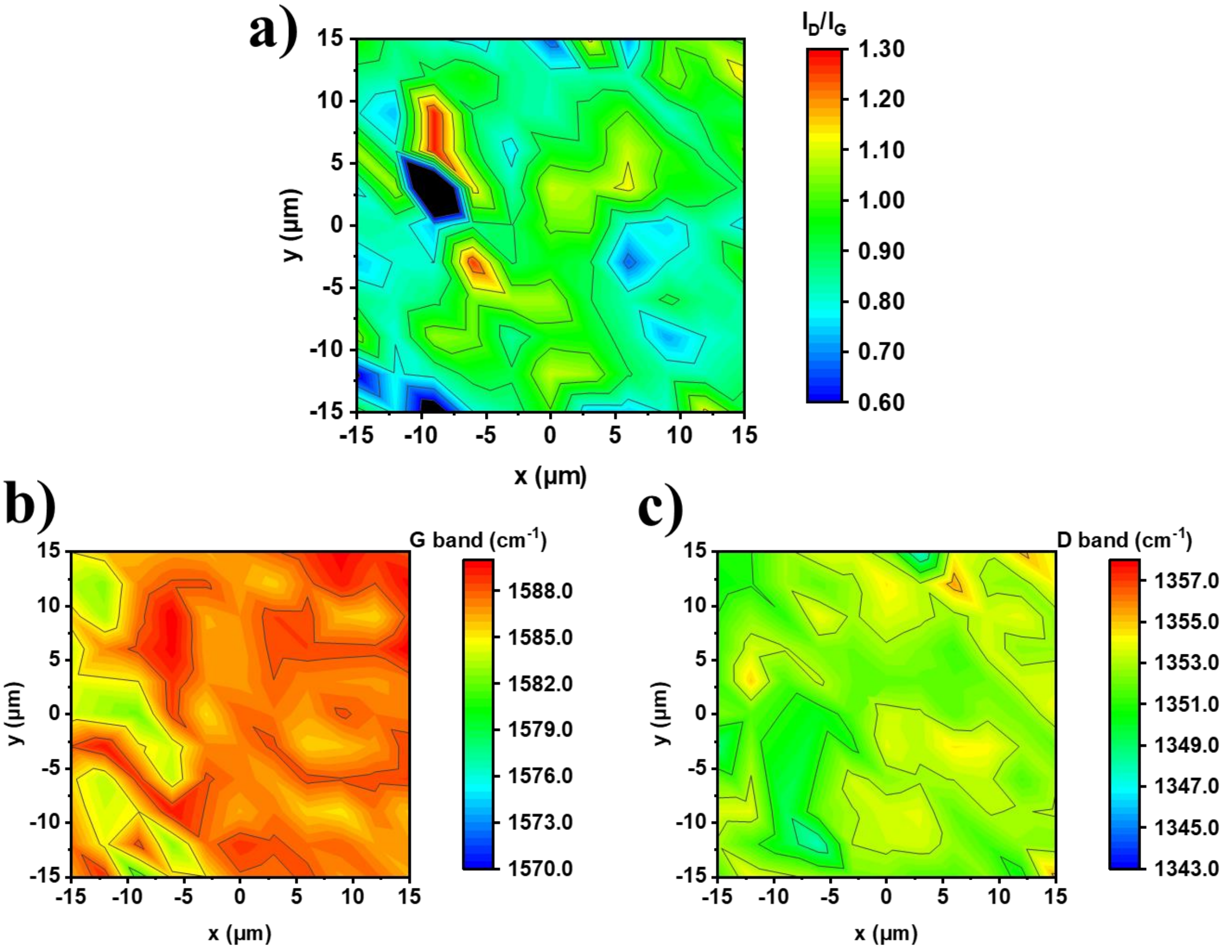

Figure S29. Raman 30 μm x 30 μm spectral maps of **7**. a) Color scale: D/G intensity ratio and b) Color scale: G (left) and D (right) band position (average G position: 1587 cm^{-1} and average D position: 1353 cm^{-1}).

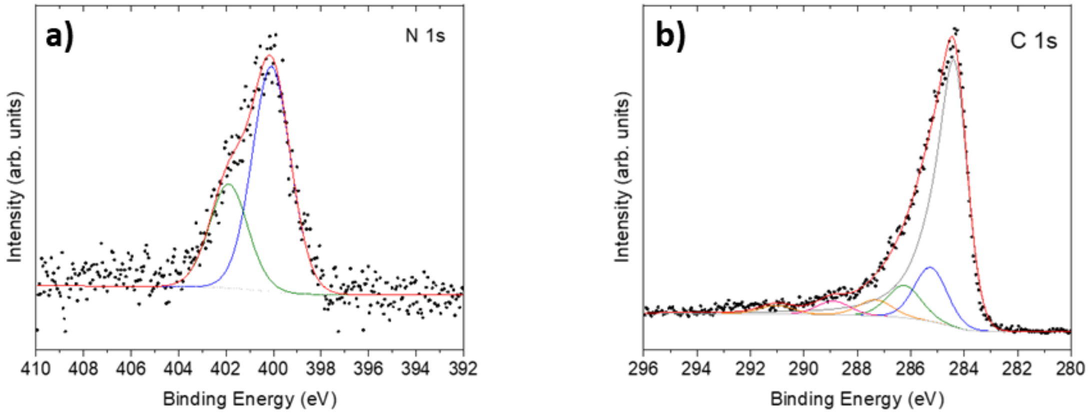

Nanomaterial 7	Normalized Areas	Atomic Percentage C : N : Cu : Pt (%)
**C 1s**	993	94.96
**N 1s**	51	4.90
**Cu 2p3/2**	0	0.00
**Pt 4f**	2	0.14

Figure S30. XPS a, b) narrow scans of N 1s and C 1s, respectively, of control electrocatalyst **7**. The table below shows normalized areas and atomic percentages calculated with high-resolution narrow scans. Pt/C ratio is 0.2%.

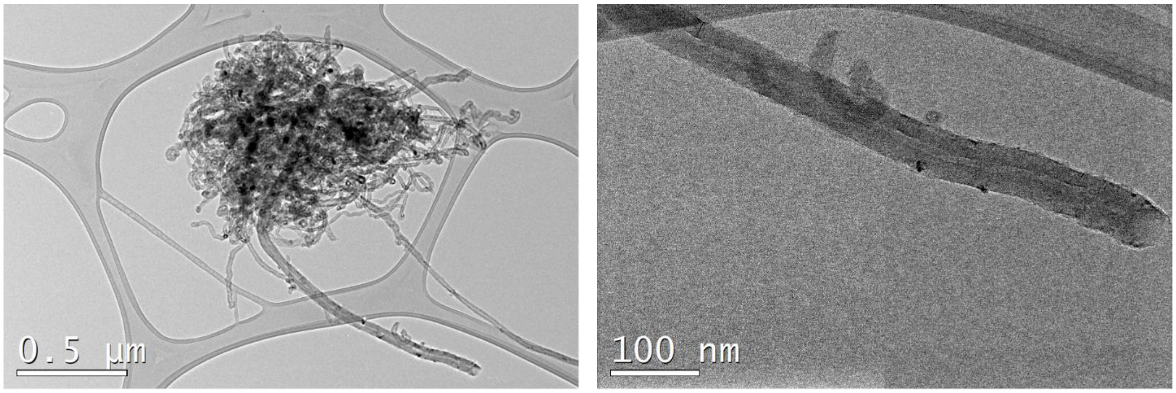

Figure S31. a, b) TEM micrographs of control electrocatalyst **7** a) scale bar: 500 nm and b) scale bar: 100 nm magnification depicting the morphology of the functionalized material.

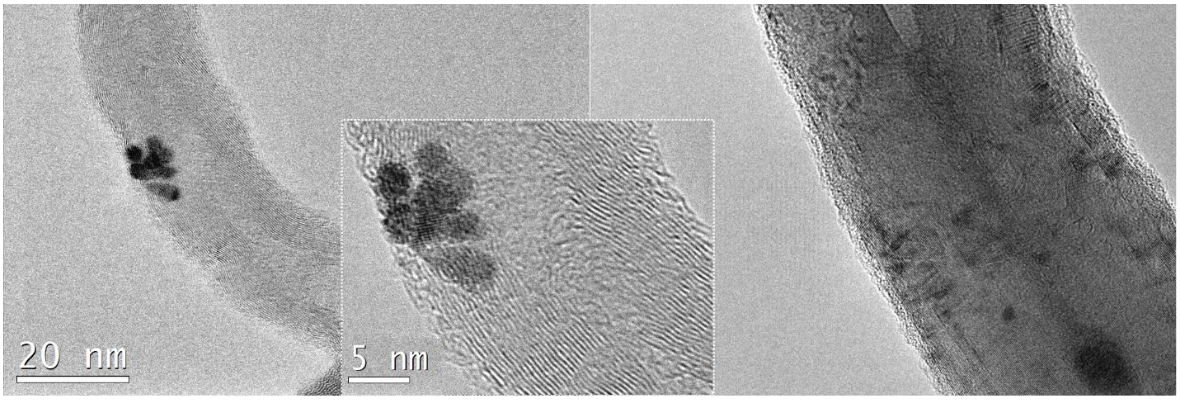

Figure S32. TEM micrographs of control electrocatalyst **7** at high (20 nm) magnification (Inset: ultra-high magnification-5 nm- of **7**).

Supplementary Note on ATR-FTIR Spectra of Ligand 4 and Nanomaterials 8 and 7

Characteristic Fingerprint Bands of **4**

The ATR-FTIR spectrum of ligand **4** exhibits several characteristic fingerprint bands in the region 1800 cm^{-1}- 1000 cm^{-1} that are associated with its chemical structure and are assigned in **Fig. S16** and **Table S1**.

Table S2. Observed ATR-FTIR bands and their assignment.

**Assignements**	**4**	**7**	**8**
**Arom. C=C (MWCNTs)**	-	1565 cm^{-1}	**1565 cm^{-1}**
**Arom. C=C (Ligand) & Imid. C=N ring**	1549 cm^{-1}, 1485 cm^{-1} and 1445 cm^{-1}	1549 cm^{-1}, and 1445 cm^{-1}	**1549 cm^{-1}**, 1485 cm^{-1} and 1445 cm^{-1}
**N=N (triazole)**	-	1406 cm^{-1}	**1406 cm^{-1}**
**Imid. C=N/C-N ring stretch.**	1374 cm^{-1}	-	**1374 cm^{-1}**
**C-N stretch.**	1215 cm^{-1}	**1215 cm^{-1}**	1215 cm^{-1}
**(N)-CH_2- stretching & C-O-C**	1147 cm^{-1}, 1066 cm^{-1}	**1147 cm^{-1}**, and **1066 cm^{-1}**	-
**CC, CH & arom. C-C ring stretch.**	1023 cm^{-1}	-	1023 cm^{-1}
**N(CH)N bend. & ring HCCH bend.**	856 cm^{-1}	865 cm^{-1}	865 cm^{-1}
**Ring CC bend**	-	-	763 cm^{-1}
**ring HCCH bend. & CC, C=C-H**	754 cm^{-1}		

Collectively, the enhancements of peaks in nanomaterial **8**, along with the pronounced 'in-ring' enhancements from the MWCNTs, suggest a strong interaction between the PtNPs and the NHCs, as well as the triazolyl moieties, all facilitated by NHC ligation. This indicates a robust integration of the PtNPs within the nanostructure. In contrast, the enhancement of the bands at 1066 cm^{-1}, 1147 cm^{-1}, and 1215 cm^{-1} in nanomaterial **7**, which are associated with the C-N and C-O stretching vibrations of the ligand chain, coupled with the absence of corresponding 'in-ring' enhancements

from the MWCNTs, suggests differences in ligation and interactions with the PtNPs, as well as variations in conformation and integration of the three distinct components (MWCNTs, triazoles and PtNPs) in nanomaterial **7**. This indicates that the overall structural and electronic properties of nanomaterial **7** differ substantially to those of nanomaterial **8**.

Table S3. XPS narrow scans of C1s. Comparative table assignments of C1s.

**C1s assignment**	**5**	**6**	**7**	**8**
**Pt-*C*-N-**	-	-	-	282.8 eV
***C*sp2**	284.4 eV	284.4 eV	284.4 eV	284.4 eV
***C*sp3**	285.3 eV	285.3 eV	285.3 eV	285.3 eV
***C*-N**	286.4 eV	286.4 eV	286.4 eV	286.4 eV
***C*-O-*C*, *C*-N+**	287.3 eV	287.3 eV	287.3 eV	287.3 eV
**adsorbed CO_2 / Oxidized carbon**	289.1 eV	289.1 eV	289.1 eV	289.1 eV
π-π* **shake-up satellite**	291.0 eV	291.0 eV	291.0 eV	291.0 eV
**N1s assignment**	**5**	**6**	**7**	**8**
**Pt-C-*N*-**	-	-	-	398.7 eV
**-*N*-N=*N*- (trz), *N* imidaz.**	400.1 eV	400.0 eV	400.1 eV	400.1 eV
**-N-*N*=N- (trz), N^+ imidaz.**	401.8 eV	401.8 eV	401.9 eV	401.8 eV

2. Electrochemical characterization of hybrid nanomaterials 1, 3, 5, 6, control electrocatalyst 7 and advanced electrocatalyst 8 against hydrogen evolution reaction

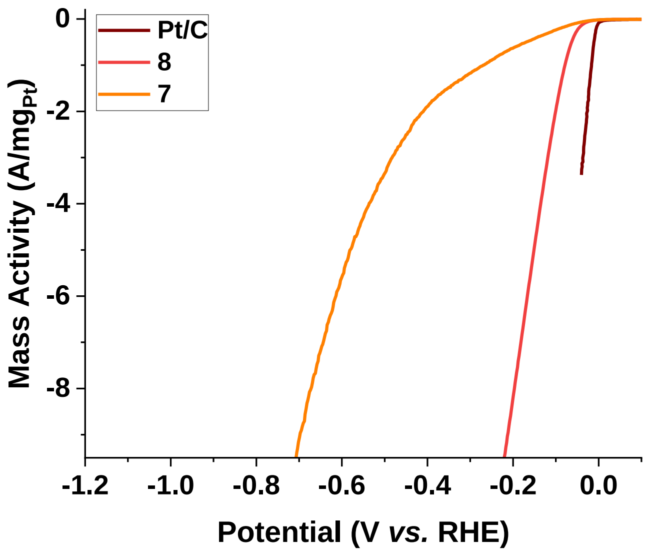

Figure S33. Polarization current density normalized by Pt mass of **8**, **7** and **Pt/C**.

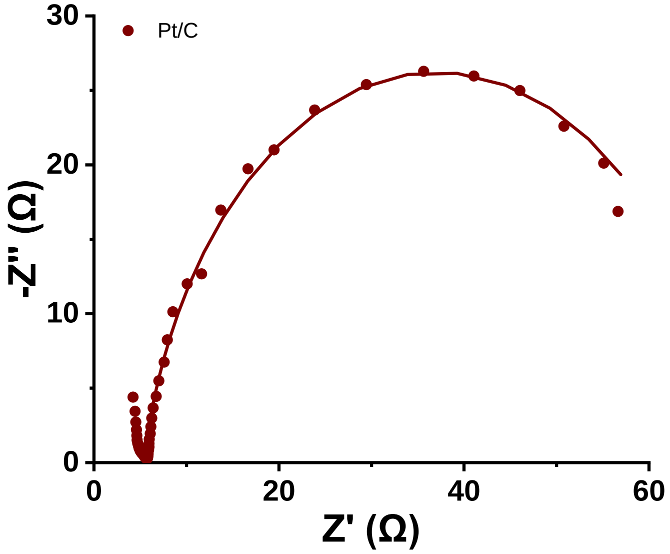

Figure S34. Fitted Nyquist plot of Pt/C 20% wt.

Table S4. Fitted with Randles circuit parameters of Nyquist plots of 1 and 5-8.

Fitted Parameters	1	5	6	7	8
**Rs (Solution Resistance, Ω)**	4.56	3.02	4.50	4.51	2.61
**Rct (Charge Transfer Resistance, Ω)**	983	793	622	281	90
**Y0 (Constant Phase Element, μS·s^N)**	10.0	19.9	65.8	133.0	86.0
**N (exponent)**	0.8	0.7	0.7	0.6	0.6

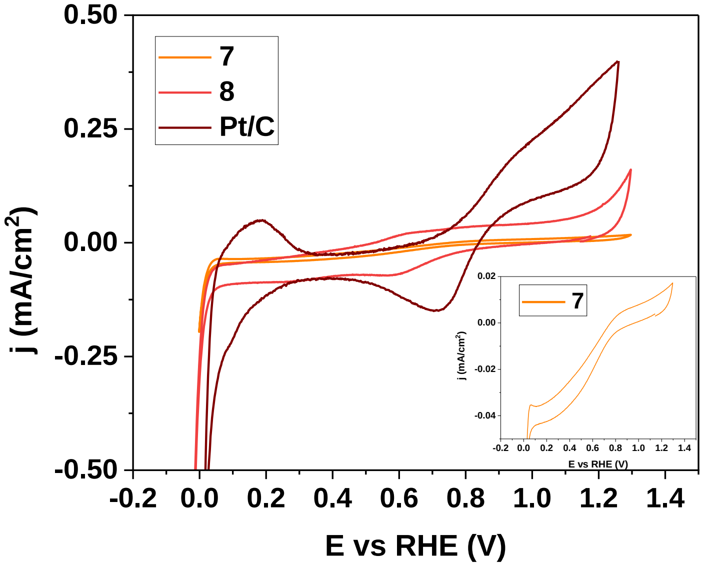

Figure S35. Cyclic Voltammograms of 7, 8 and Pt/C at 0.02 V/s, in 0.5 M H_2SO_4.

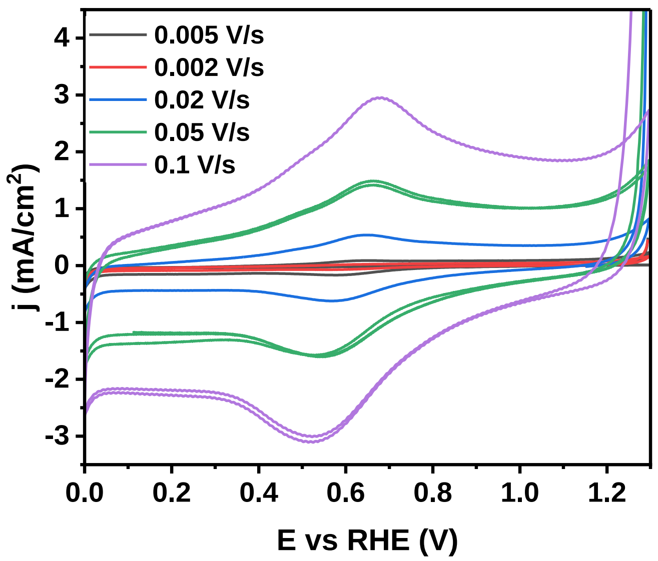

Figure S36. Cyclic Voltammograms of 8 in 0.5 M H_2SO_4, at different scan rates.

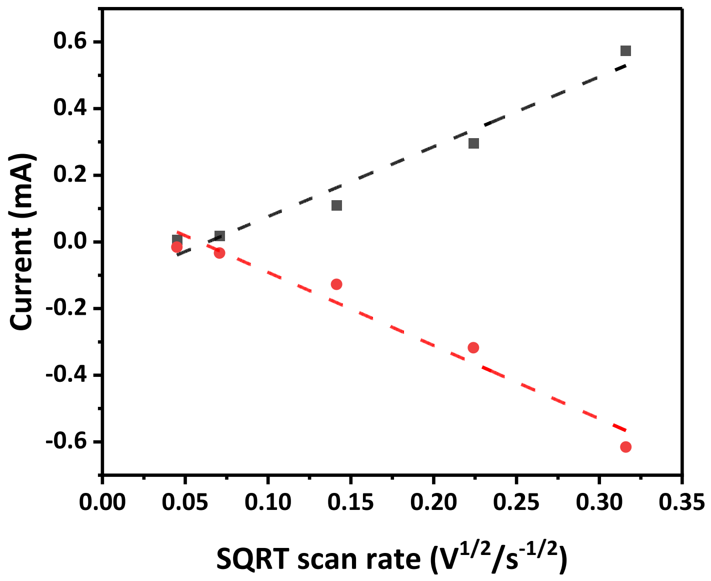

Figure S37. Current (i) versus square root of scan rate (√v) plots for PtO/Pt.

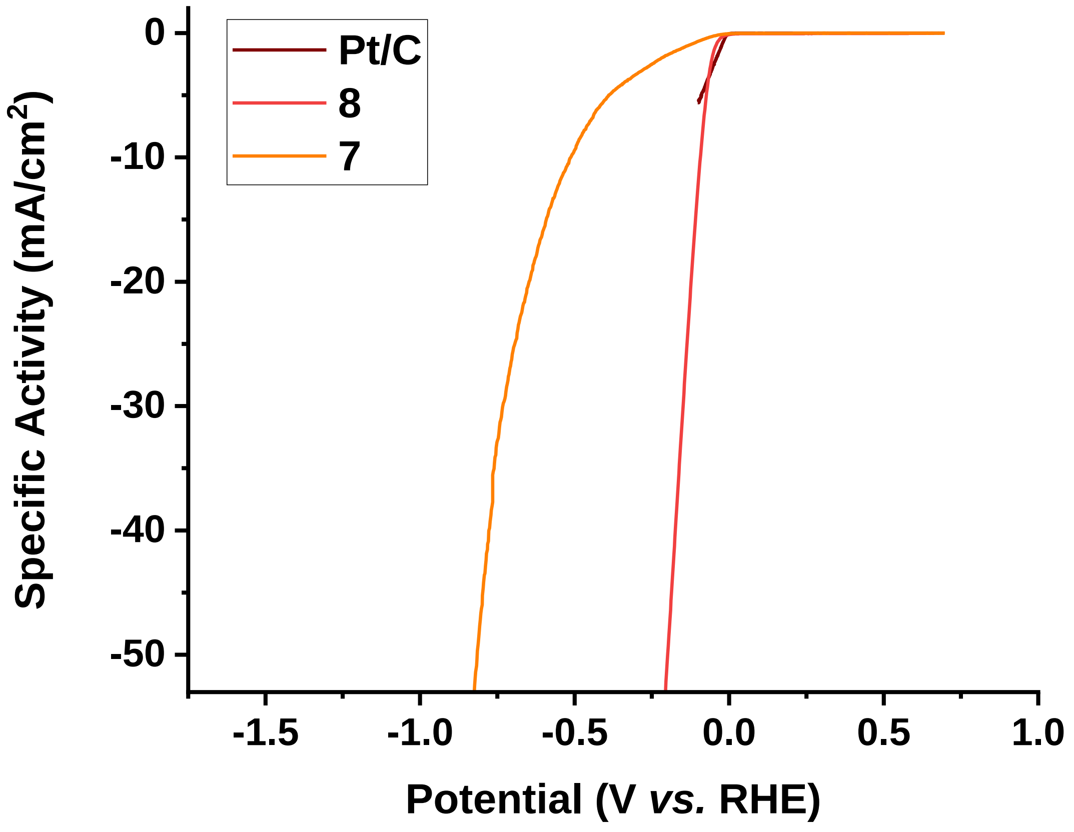

Figure S38. iR-Normalised LSVs with respect to ESCA for 7, 8 and Pt/C.

Electrochemically active surface area (ECSA) measurements for hybrid nanomaterials 1, 5, 6, control electrocatalyst 7 and advanced electrocatalyst 8 in acidic medium (0.5 M H_2SO_4).[4,5]

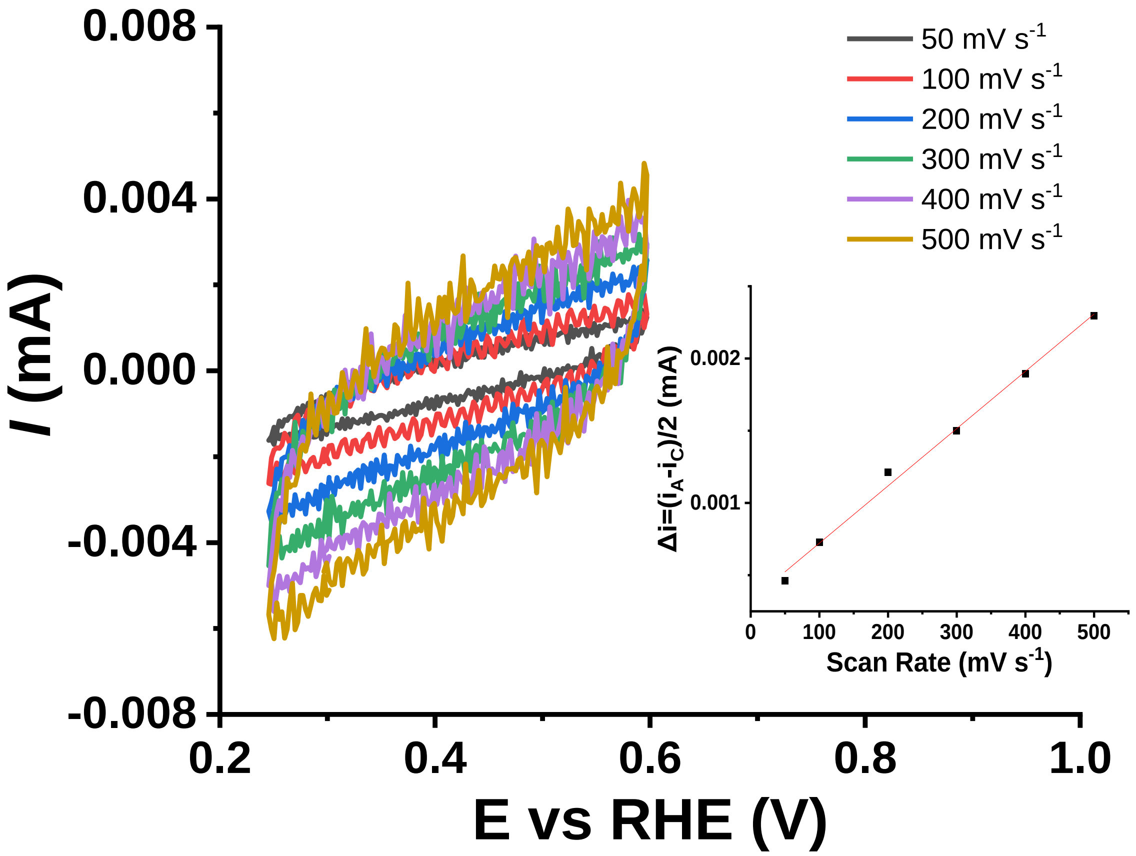

Figure S39. Cyclic voltammograms of **1** at scan rates 50-500 mV s^{-1} (Inset: Δi/2 vs scan rate for C_{dl} calculation) in N_2-saturated 0.5 M H_2SO_4 electrolyte. Double-layer capacitance (**C_{dl}**) was found to be 3.96 ± 0.148 μF and **ECSA** was estimated to be 0.099 ± 0.0037 cm^2.

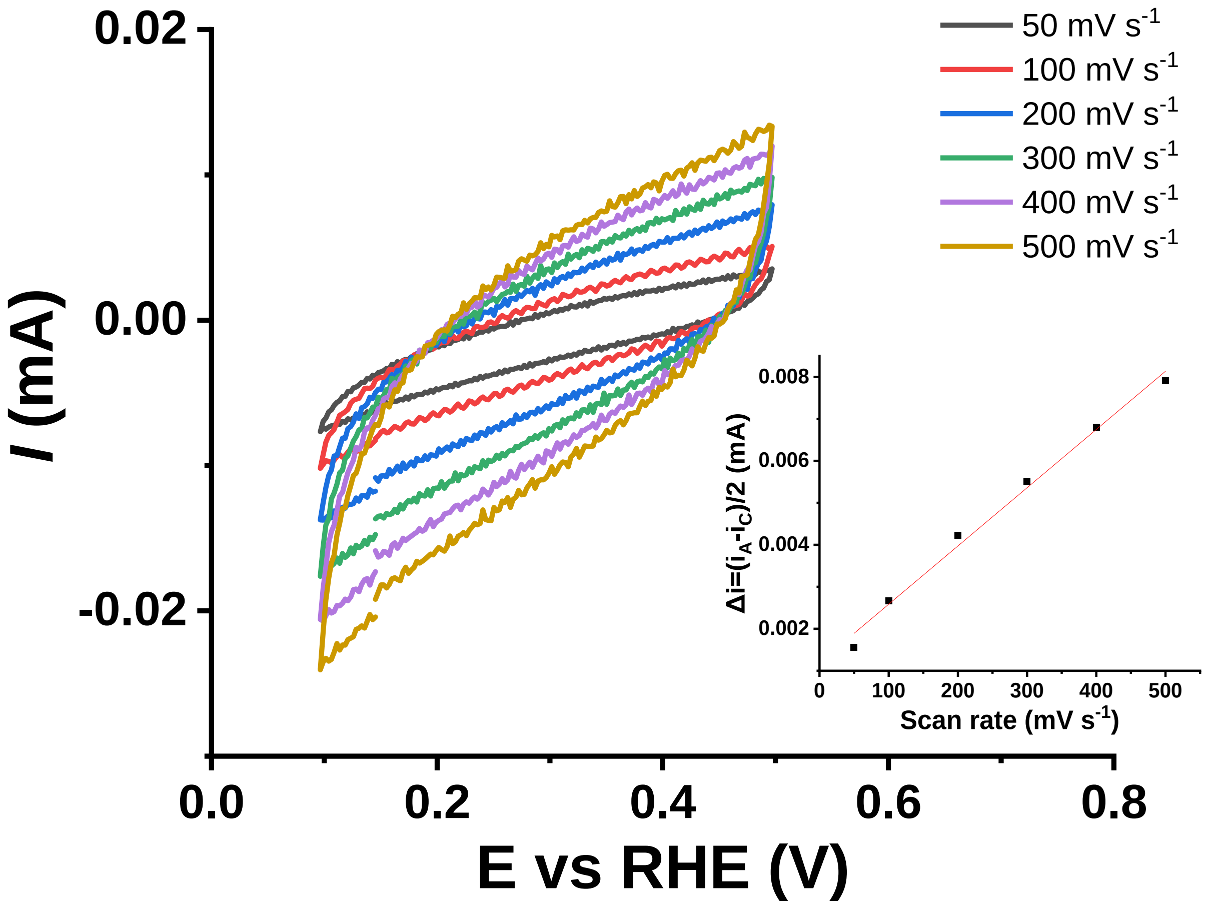

Figure S40. Cyclic voltammograms of **5** at scan rates 50-500 mV s^{-1} (Inset: Δi/2 vs scan rate for C_{dl} calculation) in N_2-saturated 0.5 M H_2SO_4 electrolyte. Double-layer capacitance (**C_{dl}**) was found to be 13.87 ± 0.656 μF and **ECSA** was estimated to be 0.346 ± 0.016 cm^2.

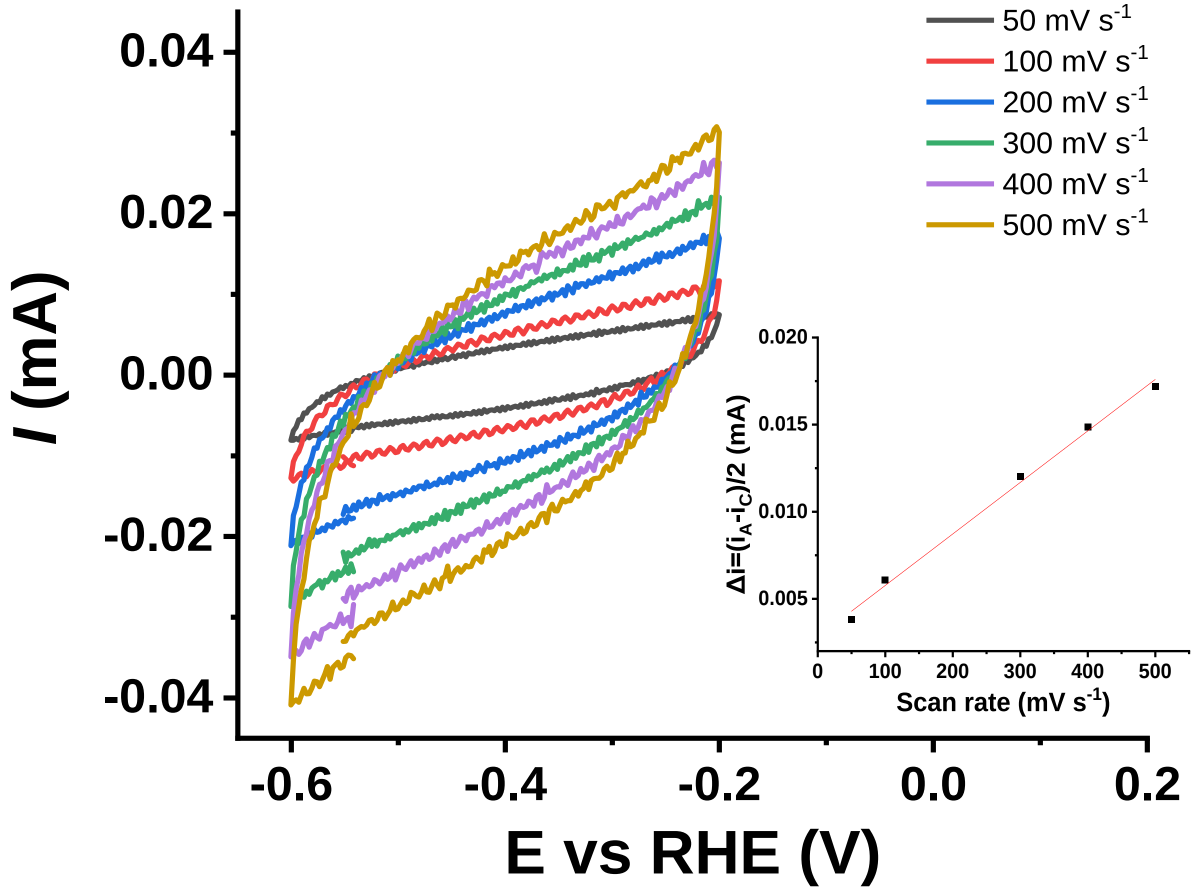

Figure S41. Cyclic voltammograms of **6** at scan rates 50-500 mV s^{-1} (Inset: Δi/2 vs scan rate for C_{dl} calculation) in N_2-saturated 0.5 M H_2SO_4 electrolyte. Double-layer capacitance (**C_{dl}**) was found to be 29.57 ± 1.218 μF and **ECSA** was estimated to be 0.739 ± 0.03 cm^2.

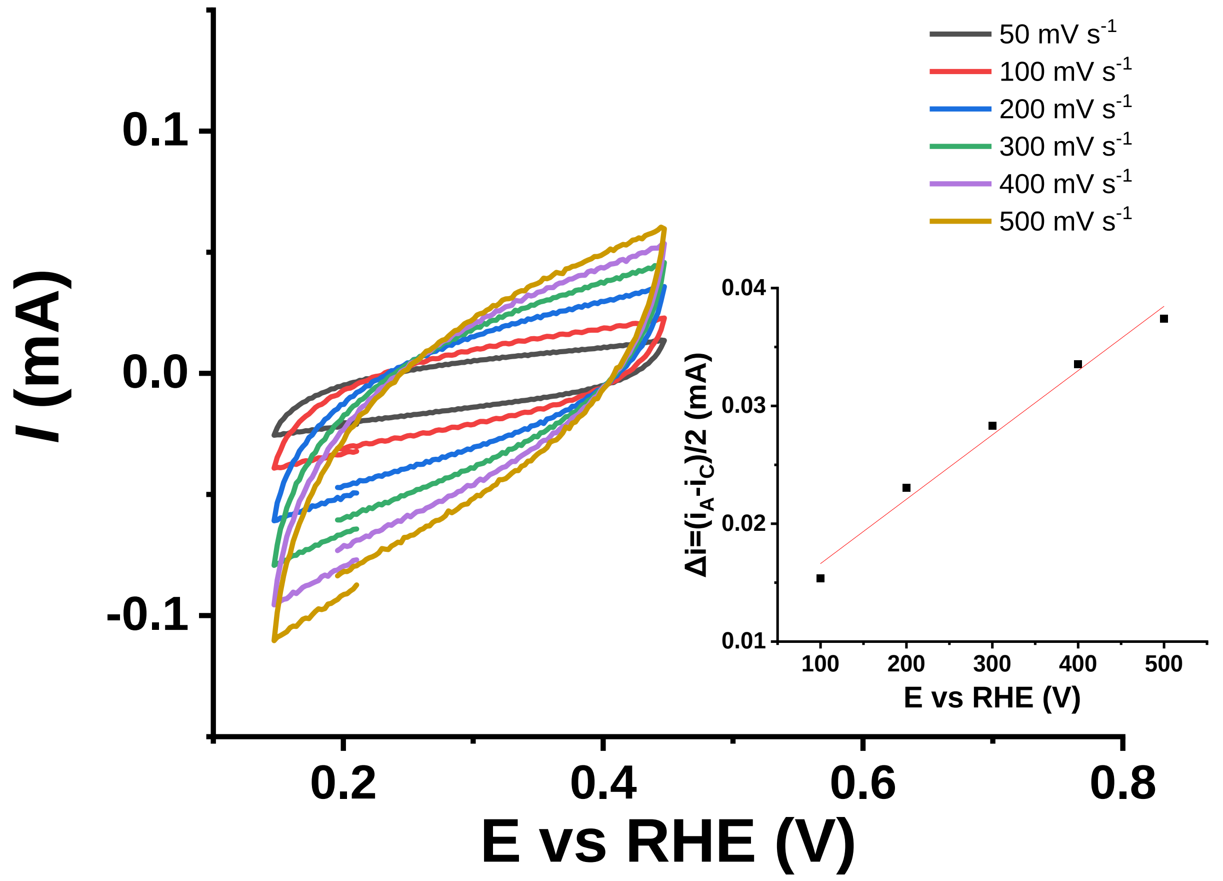

Figure S42. Cyclic voltammograms of control electrocatalyst **7** at scan rates 50-500 mV s^{-1} (Inset: Δi/2 vs scan rate for C_{dl} calculation) in N_2-saturated 0.5 M H_2SO_4 electrolyte. Double-layer capacitance (**C_{dl}**) was found to be 54.6 ± 3.89 μF and **ECSA** was estimated to be 1.365 ± 0.097 cm^2.

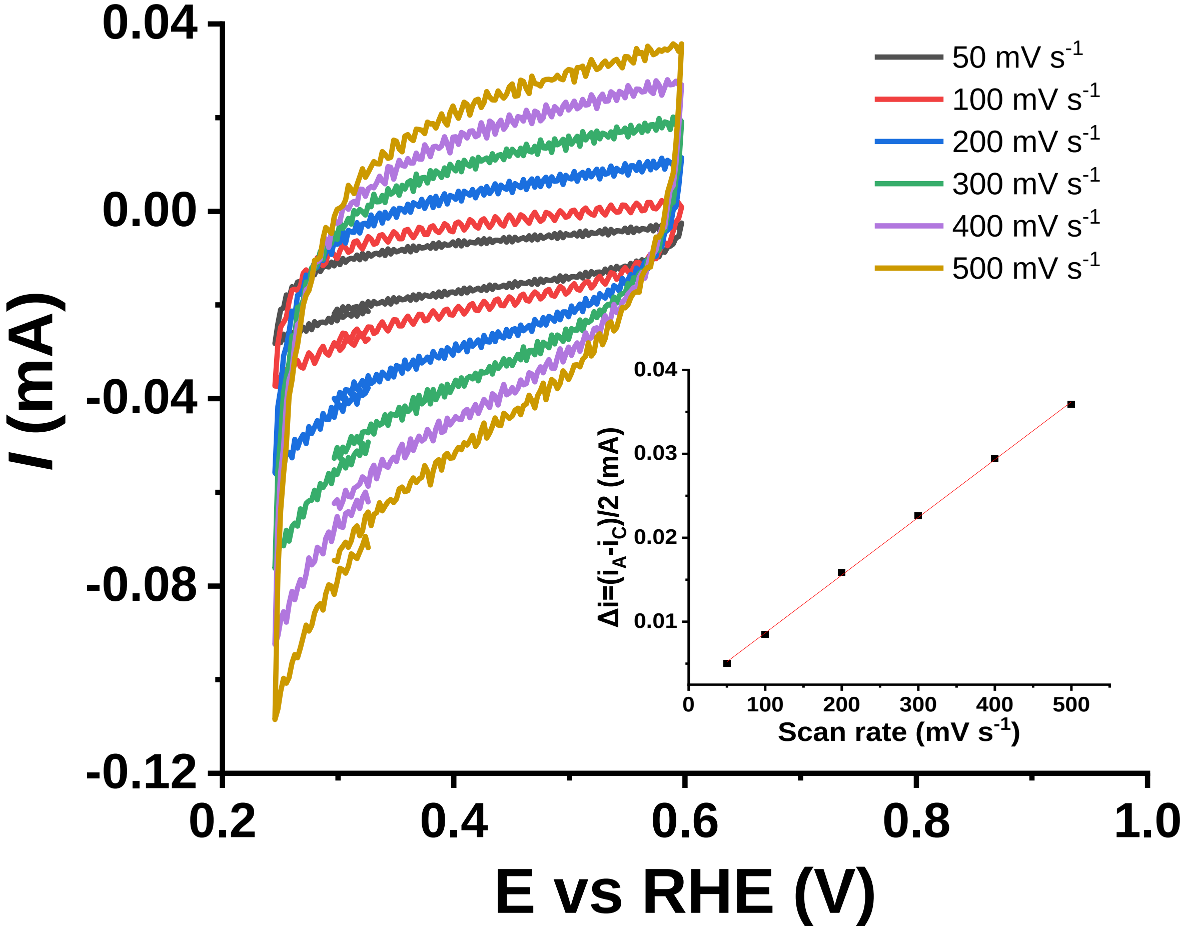

Figure S43. Cyclic voltammograms of advanced electrocatalyst **8** at scan rates 50-500 mV s^{-1} (Inset: Δi/2 vs scan rate for C_{dl} calculation) in N_2-saturated 0.5 M H_2SO_4 electrolyte. Double-layer capacitance (**C_{dl}**) was found to be 68.83 ± 3.89 μF and **ECSA** was estimated to be 1.720 ± 0.018 cm^2.

Comparative LSV polarization curves for hybrid nanomaterials 1, 5, 6, control electrocatalyst 7 and advanced electrocatalyst 8 in acidic medium (0.5 M H_2SO_4).

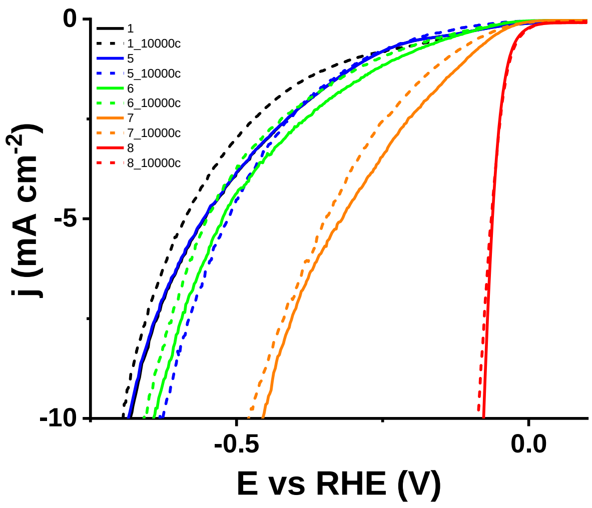

Figure S44. LSV polarization curves before (solid lines) and after 10^4 cycles (dashed lines), obtained at 1600 rpm rotation speed and 5 mV/s[1] scan rate for hybrid nanomaterials **1**, **5**, **6**, control electrocatalyst **7** and advanced electrocatalyst **8**.

Table S5. Cycling test: The cycling stability test was performed at a 50 mV/s scan rate. The potential was cycled for 10^4 times between the potential values corresponding to current densities of 0 mA/cm^2 and -10 mA/cm^2 for each catalyst. Thus, the specific potential values:

Nanomaterial	E_{start} *vs* RHE (V)	E_{stop} *vs* RHE (V)
**1**	-0.150	-0.676
**5**	-0.150	-0.683
**6**	-0.100	-0.637
**7**	0.02	-0.453
**8**	0.02	-0.077
**Pt/C**	0.02	-0.034

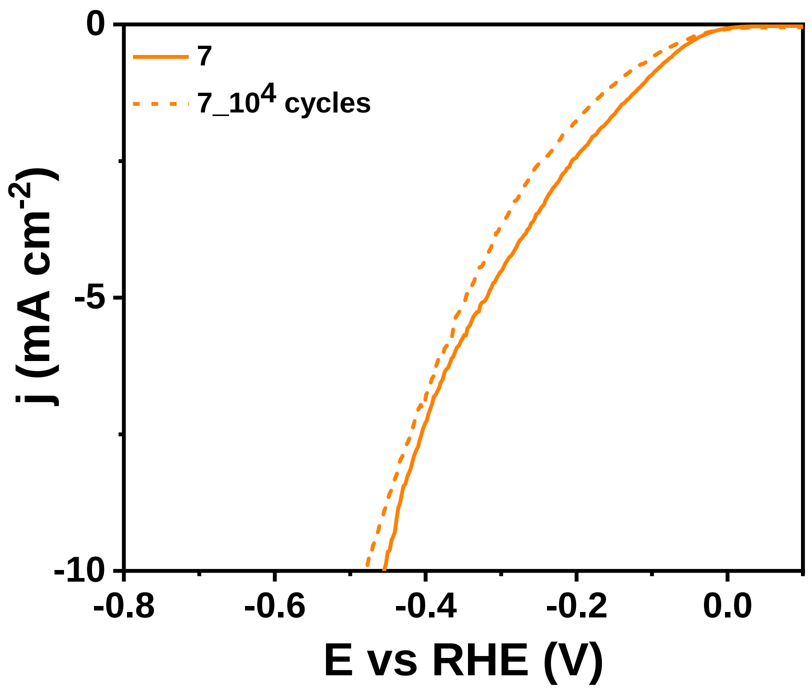

Figure S45. LSV polarization curves of **7** (solid line) and **7** after 10^4 cycles (dashed line) obtained at 1600 rpm rotation speed and 5 mV s^{-1} scan rate.

Table S6. Comparison table of the electrochemical performance of low-loading Pt-based electrocatalysts, including electrocatalyst 8 and control 7 from this work.

Electrocatalyst / Pt loading info	η_{10} (mV)	η_{100} (mV)	Mass Activity (A mg^{-1}) @η	Specific Activity (mA/cm^2) @η	Tafel slope (mV.dec^{-1})	Reference
**8** / 0.0106 mg	77	217	8.6@$_{200\ mV}$ 1.8@$_{100\ mV}$	53@$_{200\ mV}$ 15@$_{100\ mV}$	50	**This work**
**7** / 0.0039 mg	453	870	0.5@$_{100\ mV}$	0.8@$_{100\ mV}$	191	**This work**
**Pt1/NMHCS** / -	40	-	2.07 @$_{50\ mV}$	-	56	**https://doi.org/10.1002/adma.202008599**
**Pt@PCM** / 0.53 wt.%	105	-	1.6@$_{500\ mV}$	-	65	**10.1126/sciadv.aao6657**
**Pt/Co3O4** / 12.85 wt.%, 0.037 mg/cm^2	70	170 @η_{50}	1.08@$_{150\ mV}$	-	34	**https://doi.org/10.1021/acsaem.9b00787**
**Pt@PDG4** / 6.0 wt% Pt	70	-	6.0@$_{200\ mV}$	4.4@$_{200\ mV}$	27	**https://doi.org/10.1039/D1NJ04030H**
**Pt@MoS$_2$**	88	~170@ η_{35}	-	-	56	**10.1016/j.nanoen.2021.105898**

**Pt/SnS2** / 0.37 wt %	117	~330@ η_{80}	-	-	69	https://doi.org/10.1021/acsami.7b11413
**$PdSe_2$ Nanosheets**	138	-	-	-	100	https://doi.org/10.1002/adfm.202102321
**$NiCo_2P_x$/CF**	104	~200	-	-	60	https://doi.org/10.1002/adma.201605502
**Pt1@Fe-N-C** / 2.1 wt.% Pt	60	~100@ η_{30}	-	-	42	https://doi.org/10.1002/aenm.201701345
**PtNi/rGO(N)**	98	-	-	-	43	https://doi.org/10.1039/C9CY01182J
**Pt/VS_2/CP**	77	-	-	-	40	https://doi.org/10.1021/acsnano.9b10048
**Pt-graphene/Ru (0001)** d_{PtNPs} = 1.9 nm	-	-	0.11	0.42@$_{10\ mV}$	-	https://doi.org/10.1039/D0CP02793F

3. References

S1. *Org. Biomol. Chem.* **2013**, 11, 938-954.

S2. *RSC Adv.* **2017**, 7 (53), 33248–33256.

S3. *Eur. J. Org. Chem.* **2017**, 2017 (28), 4074–4084.

S4. *Nat. Commun.* **2015**, 6, 6616.

S5. *J. Am. Chem. Soc.* **2013**, 135 (45), 16977–16987.

S6. *Chem. Rev.* **2017**, 117 (10), 7053–7112.

S7. *Chem. Commun.* ***2022**, 58, 13188-13197.*
